# Climate change, malaria and neglected tropical diseases: a scoping review

**DOI:** 10.1093/trstmh/trae026

**Published:** 2024-05-10

**Authors:** Petra Klepac, Jennifer L Hsieh, Camilla L Ducker, Mohamad Assoum, Mark Booth, Isabel Byrne, Sarity Dodson, Diana L Martin, C Michael R Turner, Kim R van Daalen, Bernadette Abela, Jennifer Akamboe, Fabiana Alves, Simon J Brooker, Karen Ciceri-Reynolds, Jeremy Cole, Aidan Desjardins, Chris Drakeley, Dileepa S Ediriweera, Neil M Ferguson, Albis Francesco Gabrielli, Joshua Gahir, Saurabh Jain, Mbaraka R John, Elizabeth Juma, Priya Kanayson, Kebede Deribe, Jonathan D King, Andrea M Kipingu, Samson Kiware, Jan Kolaczinski, Winnie J Kulei, Tajiri L Laizer, Vivek Lal, Rachel Lowe, Janice S Maige, Sam Mayer, Lachlan McIver, Jonathan F Mosser, Ruben Santiago Nicholls, Cláudio Nunes-Alves, Junaid Panjwani, Nishanth Parameswaran, Karen Polson, Hale-Seda Radoykova, Aditya Ramani, Lisa J Reimer, Zachary M Reynolds, Isabela Ribeiro, Alastair Robb, Kazim Hizbullah Sanikullah, David R M Smith, GloriaSalome G Shirima, Joseph P Shott, Rachel Tidman, Louisa Tribe, Jaspreet Turner, Susana Vaz Nery, Raman Velayudhan, Supriya Warusavithana, Holly S Wheeler, Aya Yajima, Ahmed Robleh Abdilleh, Benjamin Hounkpatin, Dechen Wangmo, Christopher J M Whitty, Diarmid Campbell-Lendrum, T Déirdre Hollingsworth, Anthony W Solomon, Ibrahima Socé Fall

**Affiliations:** Big Data Institute, Oxford University, Oxford, UK; Department of Applied Mathematics and Theoretical Physics, University of Cambridge, Cambridge, UK; Division of Parasitic Diseases and Malaria, Centers for Disease Control and Prevention, Atlanta, GA, USA; Global Neglected Tropical Diseases Programme, World Health Organization, Geneva, Switzerland; The Kirby Institute, University of New South Wales, Sydney, Australia; School of Natural and Environmental Sciences, Newcastle University, Newcastle upon Tyne, UK; Department of Infection Biology, London School of Hygiene & Tropical Medicine, London, UK; The Fred Hollows Foundation, Sydney, Australia; Division of Parasitic Diseases and Malaria, Centers for Disease Control and Prevention, Atlanta, GA, USA; Global Neglected Tropical Diseases Programme, World Health Organization, Geneva, Switzerland; Division of Infection and Immunity, University of Glasgow, Glasgow, UK; Barcelona Supercomputing Center (BSC), Barcelona, Spain; British Heart Foundation Cardiovascular Epidemiology Unit, Department of Public Health and Primary Care, University of Cambridge, Cambridge, UK; Victor Phillip Dahdaleh Heart and Lung Research Institute, University of Cambridge, Cambridge, UK; Global Neglected Tropical Diseases Programme, World Health Organization, Geneva, Switzerland; Division of Parasitic Diseases and Malaria, Centers for Disease Control and Prevention, Atlanta, GA, USA; Drugs for Neglected Diseases initiative, Geneva, Switzerland; Neglected Tropical Diseases, Bill & Melinda Gates Foundation, Seattle, WA, USA; Department of Global Health, University of Washington, Seattle, WA, USA; Global Neglected Tropical Diseases Programme, World Health Organization, Geneva, Switzerland; The Fred Hollows Foundation, Suva, Fiji; Faculty of Infectious and Tropical Diseases, London School of Hygiene & Tropical Medicine, London, UK; Department of Infection Biology, London School of Hygiene & Tropical Medicine, London, UK; CHICAS, Lancaster University, Lancaster, UK; Faculty of Medicine, University of Kelaniya, Kelaniya, Sri Lanka; School of Public Health, Imperial College London, London, UK; Global Neglected Tropical Diseases Programme, World Health Organization, Geneva, Switzerland; Queen Elizabeth Hospital, Lewisham and Greenwich NHS Trust, London, UK; Global Neglected Tropical Diseases Programme, World Health Organization, Geneva, Switzerland; Environmental Health and Ecological Sciences Department, Ifakara Health Institute, Dar es Salaam, United Republic of Tanzania; Expanded Special Project for Elimination of Neglected Tropical Diseases, Regional Office for Africa, World Health Organization, Brazzaville, Republic of Congo; Global Institute for Disease Elimination, Abu Dhabi, United Arab Emirates; Department of Neglected Tropical Diseases, Children's Investment Fund Foundation, Addis Ababa, Ethiopia; Global Neglected Tropical Diseases Programme, World Health Organization, Geneva, Switzerland; Environmental Health and Ecological Sciences Department, Ifakara Health Institute, Dar es Salaam, United Republic of Tanzania; Environmental Health and Ecological Sciences Department, Ifakara Health Institute, Dar es Salaam, United Republic of Tanzania; Research and Knowledge Management, Pan-African Mosquito Control Association, Nairobi, Kenya; Global Malaria Programme, World Health Organization, Geneva, Switzerland; Pure and Applied Mathematics, Jomo Kenyatta University of Agriculture and Technology, Nairobi, Kenya; Mathematics, Statistics and Actuarial Science, Karatina University, Karatina, Kenya; Environmental Health and Ecological Sciences Department, Ifakara Health Institute, Dar es Salaam, United Republic of Tanzania; Global Leprosy Programme, World Health Organization, New Delhi, India; Barcelona Supercomputing Center (BSC), Barcelona, Spain; Catalan Institution for Research and Advanced Studies (ICREA), Barcelona, Spain; Centre on Climate Change & Planetary Health and Centre for Mathematical Modelling of Infectious Diseases, London School of Hygiene & Tropical Medicine, London, UK; Environmental Health and Ecological Sciences Department, Ifakara Health Institute, Dar es Salaam, United Republic of Tanzania; Global Strategic Partnerships, The END Fund, New York, NY, USA; Médecins Sans Frontières, Operational Centre Geneva, Geneva, Switzerland; Institute for Health Metrics and Evaluation, University of Washington, Seattle WA, USA; Department of Communicable Diseases Prevention, Control and Elimination, Pan American Health Organization, Washington DC, USA; Big Data Institute, Oxford University, Oxford, UK; BeVera Solutions LLC, Riverdale GA, USA; Division of Parasitic Diseases and Malaria, Centers for Disease Control and Prevention, Atlanta, GA, USA; Department of Social and Environmental Determinants of Health Equity, Pan American Health Organization, Washington DC, USA; Big Data Institute, Oxford University, Oxford, UK; Department of Pathobiology and Population Sciences, Royal Veterinary College, Hatfield, UK; Division of Parasitic Diseases and Malaria, Centers for Disease Control and Prevention, Atlanta, GA, USA; Synergy America Inc, Atlanta GA, USA; Drugs for Neglected Diseases initiative, Geneva, Switzerland; Global Malaria Programme, World Health Organization, Geneva, Switzerland; Integrated Communicable Disease Unit, Regional Office for the Western Pacific, World Health Organization, Manilla, Philippines; Health Economics Research Centre, Nuffield Department of Population Health, University of Oxford, UK; Environmental Health and Ecological Sciences Department, Ifakara Health Institute, Dar es Salaam, United Republic of Tanzania; School of Computational and Communication Science and Engineering, The Nelson Mandela African Institution of Science and Technology, Arusha, United Republic of Tanzania; Department of Epidemiology and Public Health, Swiss Tropical and Public Health Institute, Basel, Switzerland; Division of Neglected Tropical Diseases, Global Health Bureau, United States Agency for International Development, Washington DC, USA; Science Department, World Organisation for Animal Health, Paris, France; Department of Communications, Uniting to Combat Neglected Tropical Diseases, London, UK; Wellcome, London, UK; The Kirby Institute, University of New South Wales, Sydney, Australia; Global Neglected Tropical Diseases Programme, World Health Organization, Geneva, Switzerland; Neglected Tropical Disease Control, Regional Office for the Eastern Mediterranean, World Health Organization, Cairo, Egypt; Office of Development Affairs, Presidential Court, Abu Dhabi, United Arab Emirates; Vector-Borne and Neglected Tropical Diseases Control, Regional Office for South-East Asia, World Health Organization, New Delhi, India; Ministère de la Santé, Djibouti City, Djibouti; Ministère de la Santé, Cotonou, Bénin; Ministry of Health, Thimphu, Bhutan; Office of the Chief Medical Advisor, Government of the United Kingdom, London, UK; Climate Change and Health, World Health Organization, Geneva, Switzerland; Big Data Institute, Oxford University, Oxford, UK; Global Neglected Tropical Diseases Programme, World Health Organization, Geneva, Switzerland; Global Neglected Tropical Diseases Programme, World Health Organization, Geneva, Switzerland

## Abstract

To explore the effects of climate change on malaria and 20 neglected tropical diseases (NTDs), and potential effect amelioration through mitigation and adaptation, we searched for papers published from January 2010 to October 2023. We descriptively synthesised extracted data. We analysed numbers of papers meeting our inclusion criteria by country and national disease burden, healthcare access and quality index (HAQI), as well as by climate vulnerability score. From 42 693 retrieved records, 1543 full-text papers were assessed. Of 511 papers meeting the inclusion criteria, 185 studied malaria, 181 dengue and chikungunya and 53 leishmaniasis; other NTDs were relatively understudied. Mitigation was considered in 174 papers (34%) and adaption strategies in 24 (5%). Amplitude and direction of effects of climate change on malaria and NTDs are likely to vary by disease and location, be non-linear and evolve over time. Available analyses do not allow confident prediction of the overall global impact of climate change on these diseases. For dengue and chikungunya and the group of non-vector-borne NTDs, the literature privileged consideration of current low-burden countries with a high HAQI. No leishmaniasis papers considered outcomes in East Africa. Comprehensive, collaborative and standardised modelling efforts are needed to better understand how climate change will directly and indirectly affect malaria and NTDs.

## Introduction

Human activity is driving incremental changes in climate patterns globally. The burning of coal, oil and natural gas; clearing land by cutting down forests; and intensified agriculture all release greenhouse gases, primarily CO_2_, methane and N_2_O. Deforestation also reduces capacity for CO_2_ absorption. Increases in atmospheric concentrations of greenhouse gases are driving mean global temperatures upwards, with consequent effects including rising sea levels, changes in rainfall and increases in the frequency and intensity of extreme weather events.^1^

Climate change will perturb human health in profound and long-lasting ways, both directly and indirectly.^2^ Multiple direct and indirect mechanisms will contribute. Human physiology can be affected directly by changes in air temperature, humidity, stress and diet. Human behaviour can be altered by changing weather, changing economic circumstances, migration, natural disasters and access to or quality of healthcare. Environmental conditions also influence pathogen transmission, reproduction, development and genetics; the reproduction, development, genetics, behaviour, range, longevity and predation of vectors, intermediate hosts and reservoir hosts; and the feasibility and effectiveness of interventions. In fact, there is already empirical evidence of climate change having amplified more than one-half of all known human infectious diseases.^3^

The impact of climate change is likely to be disproportionately borne by the poorest people, who are also disproportionately affected by malaria and neglected tropical diseases (NTDs; Table 1). In part because of this association with poverty, malaria and NTDs are often co-endemic. Many of these diseases are currently suitable for coordinated control via integrated programmes.^4–7^

**Table 1.** Diseases and organisms in scope for this review. Included diseases and associated aetiological agents, vectors, reservoir and intermediate hosts and routes of transmission are those listed in the NTD road map 2021–2030,^13^ plus malaria. Noma was added to the NTD list in December 2023,^61^ and therefore is not included here.

Disease(s)	Pathogen type	Aetiological agent(s)	Vector(s)	Reservoir/intermediate host(s)	Route(s) of transmission to humans
Buruli ulcer	Bacterium	*Mycobacterium ulcerans*		Common ringtail possums, common brushtail possums (southeast Australia)	Not known
Chagas disease	Protozoan	*Trypanosoma cruzi*	Triatomine bugs (particularly *Triatoma infestans, Rhodnius prolixus, Triatoma dimidiata, Panstrongylus megistus*)	Wild mammals	In the Americas: (i) from the faeces and urine of triatomine bugs; and everywhere: (ii) oral/foodborne, (iii) congenital, (iv) in blood products and transplanted organs, and (v) laboratory accidents
Dengue, chikungunya	Virus	Dengue virus, chikungunya virus	*Aedes* mosquitos (particularly *Ae. aegypti, Ae. albopictus, Ae. polynesiensis, Ae. scutellaris*)	Non-human primates (southeast Asia and West Africa)	Being bitten by an infected female *Aedes* spp. mosquito
Dracunculiasis (Guinea-worm disease)	Helminth	*Dracunculus medinensis*	*Cyclops* spp. (water fleas)	Fish, dogs, cats, baboons	Drinking water containing infected *Cyclops* spp.
Echinococcosis	Helminth	*Echinococcus granulosus, E. multilocularis, E. oligarthrus, E. vogeli*		Dogs and other carnivores; sheep, goats, cows, pigs, wild herbivores, rodents	Ingestion of food or water containing eggs released in the stool of an infected carnivore, or close contact with an infected carnivore
Foodborne trematodiases (clonorchiasis, fascioliasis, opisthorchiasis, paragonimiasis)	Helminth	*Clonorchis sinensis, Fasciola gigantica, F. hepatica, Opisthorchis felineus, O. viverrini, Paragonimus* spp.		Freshwater snails, freshwater crustaceans, fish, herbivorous mammals (for *Fasciola* spp.), piscivorous mammals	Ingestion of raw or undercooked food (freshwater fish, aquatic vegetables, crabs, crayfish) infected with larvae
Human African trypanosomiasis	Protozoan	*Trypanosoma brucei gambiense, T. b. rhodesiense*	Tsetse flies (*Glossina* spp.)	Pigs and other animals (*T. b. gambiense*); cattle and wild herbivores (*T. b. rhodesiense*)	Being bitten by an infected tsetse fly; mother-to-child transmission
Leishmaniasis (visceral, cutaneous or mucocutaneous, post-kala azar dermal leishmaniasis)	Protozoan	*Leishmania aethiopica, L. amazonensis, L. braziliensis, L. chagasi, L. colombiensis, L. donovani, L. garnhami, L. guyanensis, L. infantum, L. killicki, L. lainsoni, L. lindenbergi, L. major, L. mexicana, L. naiffi, L. panamensis, L. peruviana, L. pifanoi, L. shawi, L. tropica, L. venezuelensis*	Phlebotomine sandflies	Wild and domestic mammals (e.g. dogs)	Being bitten by an infected female phlebotomine sandfly
Leprosy (Hansen's disease)	Bacterium	*Mycobacterium leprae, M. lepromatosis*		Nine-banded armadillos, red squirrels	Likely droplet spread during prolonged close contact with an untreated patient
Lymphatic filariasis	Helminth	*Wuchereria bancrofti, Brugia malayi, B. timori*	*Culex, Anopheles, Mansonia, Aedes* mosquitos		Being bitten by an infected female mosquito
Malaria	Protozoan	*Plasmodium falciparum, P. vivax, P. ovale, P. malariae, P. knowlesi*	*Anopheles* mosquitos	Wild non-human primates (*P. knowlesi*)	Being bitten by an infected female *Anopheles* mosquito
Mycetoma, chromoblastomycosis, paracoccidioidomycosis, sporotrichosis	Fungus/bacterium	Various fungi and bacteria, notably *Paracoccidioides lutzii, Fonsecaea pedrosoi, Cladophialophora carrionii, Phialophora verrucose, Madurella mycetomatis, Nocardia brasiliensis, Actinomadura madurae, Streptomyces somaliensis, Actinomadura pelletieri, Sporothrix schenckii*			Not well understood (mycetoma); traumatic inoculation of relevant pathogens through broken skin (chromoblastomycosis, paracoccidioidomycosis, sporotrichosis)
Onchocerciasis	Helminth	*Onchocerca volvulus*	*Simulium* blackflies		Being bitten by infected *Simulium* blackflies
Rabies	Virus	Rabies virus		Dogs, bats, other wild mammals	Being bitten by infected domestic dogs (up to 99% of infections)
Scabies, tungiasis	Ectoparasite	*Sarcoptes scabiei, Tunga penetrans*		Dogs, cats, pigs, synanthropic rodents, donkeys, monkeys, birds, elephants, sheep, cows (tungiasis)	Close contact with the skin of an infected human (scabies); contact with infected sand (tungiasis)
Schistosomiasis	Helminth	*Schistosoma guineensis, S. haematobium, S. intercalatum, S. japonicum, S. mansoni, S. mekongi*	Aquatic snails, particularly of the genera *Bulinus, Biomphalaria, Oncomelania, Robertsiella* and *Neotricula*	Cows, dogs, goats, pigs, rats, water buffalo (*S. japonicum*); dogs (*S. mekongi*)	Contact with water (bathing, swimming, washing clothes, fishing) infested with cercariae
Soil-transmitted helminthiases	Helminth	*Ascaris lumbricoides, Ancylostoma duodenale, Necator americanus, Strongyloides stercoralis, Trichuris trichiura^a^*			Ingestion of eggs from the faeces of infected humans (*A. lumbricoides, T. trichiura*); larval penetration of intact skin (*A. duodenale, N. americanus, S. stercoralis*)
Snakebite envenoming	Non- infectious	Toxins in the bite of venomous snakes			Being bitten by a venomous snake
Taeniasis and cysticercosis	Helminth	*Taenia solium*		Pigs	Ingestion of undercooked pork (taeniasis); ingestion of eggs from the faeces of an infected human (cysticercosis)
Trachoma	Bacterium	*Chlamydia trachomatis*	*Musca sorbens* flies		Mechanical transfer of infected eye or nose secretions (via flies, fomites or fingers) to the eyes
Yaws	Bacterium	*Treponema pallidum pertenue*			Skin to skin contact via scrapes or cuts

^a^ *Ancylostoma ceylanicum* is a soil-transmitted helminth species previously believed to only infect dogs but now recognised as a zoonosis that causes symptomatic infections in humans; it was not included in the NTD road map 2021–2030^13^ and therefore was not included in this review.

Developing an understanding of the likely effects of climate change on the epidemiology of malaria and NTDs is critical to minimising their health implications. This scoping review explores current predictions of the effects of historical and future climate change on malaria and NTDs, and the potential amelioration of these effects through climate change mitigation and adaptation strategies.

## Methods

A comprehensive scoping review was conducted. To complete the review efficiently but with high fidelity, we used automation and artificial intelligence-based tools to help define search terms, translate them across different platforms, de-duplicate search results and conduct title-and-abstract screening.^8^ We did not otherwise use artificial intelligence assistance. This report adheres to the Preferred Reporting Items for Systematic Reviews and Meta-Analyses extension for Scoping Reviews guidance^9^ (Supplementary Table 1).

### Search strategy

Searches were conducted in five electronic databases that index the published peer-reviewed and grey literature: PubMed, Scopus, Embase via Ovid, Global Index Medicus and the WHO's Institutional Repository for Information Sharing.

Databases were searched for papers published from 1 January 2010 to 12 October 2023. No language or other restrictions were applied. Using a combination of Medical Subject Heading (MeSH) and free-text terms, search vocabulary related to ‘climate change’, ‘malaria’ and ‘neglected tropical diseases’ was employed. Searches were designed and conducted in English and included scientific and lay expressions for diseases, pathogens, vectors, reservoir hosts and intermediate hosts; the search strategy was first constructed for PubMed, and the Systematic Review Accelerator Polyglot tool (https://sr-accelerator.com/#/polyglot)^10^ was then used to translate strings into syntax appropriate for other databases. (Please see the Supplementary material for full search strings.) Using the citationchaser Shiny app (https://www.eshackathon.org/software/citationchaser.html),^11^ forward and backward screening of relevant seed papers (e.g. reviews, commentaries) was undertaken in December 2023 to identify additional records. The review protocol was not published in advance.

### Criteria for paper selection

Inclusion criteria applied to screening are shown in Table 2. Papers not meeting these criteria were excluded.

**Table 2.** Characteristics of included papers.

	Climate change analysis	Climate change mitigation and adaptation analysis
Population	Humans, any gender, any age: could be limited to one or more specific human population groups; OR Any life cycle stage of a vector or of an infectious agent causing malaria and/or one or more NTDs; OR Reservoir or intermediate host of an infectious agent causing one or more NTDs	
Intervention/exposure	*Alterations in temperature, humidity, rainfall, flooding, wildfires, storms, sea level, land cover or extreme weather related to anthropogenic climate change	Measures aiming to minimise climate change, or measures aiming to improve interventions or the policies around interventions in the face of climate change
Comparator/context	Reference or baseline (study-defined)	Exposure to climate change variables (*asterisked cell above) without climate change mitigation or adaptation strategies
Outcome	Prevalence, incidence, burden, hazard, vulnerability, risk, exposure, range, distribution, suitability, transmission, density	
Type of study	Modelling and statistical studies, cohort studies, laboratory studies, field studies, ecological case reports	

Papers were eligible for inclusion if they explicitly reported on the observed or modelled effects of historical or future climate change on outcomes framing the distribution, dynamics or transmission of malaria or NTDs; or the range, abundance or transmission potential of their vectors, reservoir hosts or intermediate hosts. Papers were also included if they explicitly reported on the observed or modelled effects of climate change mitigation and adaptation strategies on these outcomes. (‘Climate change mitigation’ was defined as actions intended to reduce the magnitude of climate change, and ‘adaptation’ as actions intended to reduce the vulnerability of human and natural systems to the effects of climate change or to cope with past or future climate change.)

Masters and PhD theses were eligible for inclusion if the analyses were not also presented elsewhere, but we did not specifically search thesis repositories.

Papers were excluded if (i) they did not include original analyses; (ii) they presented methods or protocols without results; (iii) they were clinical case reports; (iv) they were conference proceedings; (v) they were pre-prints (of papers being prepared for, or in the process of being considered by, peer-reviewed journals); or (vi) the full text could not be obtained. In contrast to the exclusion of clinical case reports, we included ecological case reports as potentially important indicators of pathogen or vector range expansion.

### Screening and extraction procedures

The Covidence software package (https://www.covidence.org/) was used to support record indexing, removal of duplicate records, title-and-abstract screening, full-text screening and compilation of extracted data.

After de-duplication, titles and abstracts of each record were independently screened by two researchers, with conflicts resolved by a third independent reviewer. Non-English records were translated or reviewed by a fluent speaker of that language.

The Covidence title-and-abstract screening module incorporates a machine-learning algorithm that ranks papers that are yet to be screened according to their acceptance likelihood: based on previous screening decisions, it pushes papers that are more likely to be accepted towards the top of the pile, for all reviewers. This accelerates the screening process by removing the need to screen all search results. The predetermined stopping rule was to discontinue title-and-abstract screening after three criteria were met: (i) more than twice the estimated fraction of relevant records from a previously published systematic review of climate change and NTDs^12^ had been screened; (ii) all papers previously determined as relevant (‘seed’ papers) were identified; and (iii) two screeners had each rejected 50 papers in a row. The results of the backward and forward citation searches of seed papers were screened separately with the assistance of the same (trained) machine-learning algorithm, with screening of these records discontinued after a single screener had rejected 100 papers in a row.

Full texts were blind double-screened, with discrepant results arbitrated by an independent third reviewer.

Data from papers selected in the full-text review were independently extracted by one investigator using a pre-piloted standardised form, according to self-identified individual expertise, and checked by a second investigator. Data extraction was limited to the information provided in the published work; authors were not contacted for further clarification or provision of missing information. Extracted information included: first author, year, study title, diseases addressed in the publication, study design (cohort, field, model, laboratory research, ecological case report), whether the paper explicitly discussed adaptation or mitigation strategies, the time frame of the study (cross-sectional, prospective longitudinal, retrospective longitudinal; the length of the time series), location data, and the population, intervention/exposure, comparator and outcome (Table 2).

Structured assessment of the methodological limitations and risk of bias of included individual papers was not undertaken.

### Data analyses and synthesis

Because of the heterogeneity of included studies in terms of designs, exposures, outcomes and quality, no meta-analyses were attempted. Data were narratively synthesised and thematically categorised to facilitate analysis.

**Figure 1.**
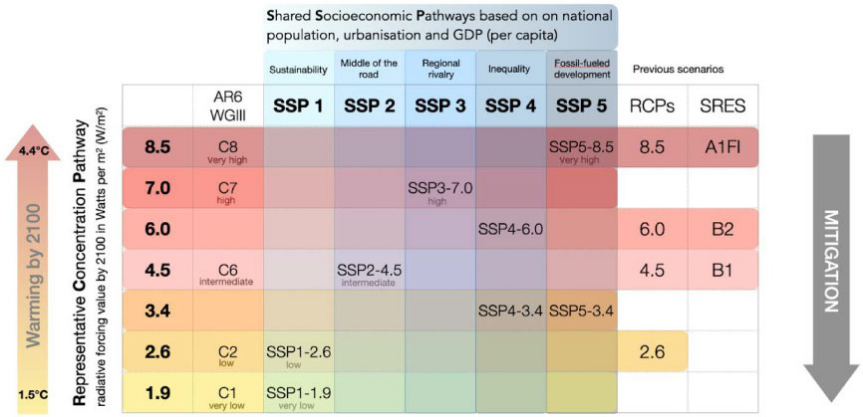
Comparison of Intergovernmental Panel on Climate Change (IPCC) scenarios used in papers that met this review's inclusion criteria. The different categories of scenario (special report on emission scenarios [SRES; released in 2000]; representative concentration pathways [RCPs; 2014]; shared socioeconomic pathways [SSP; 2021]; scenarios from the IPCC Sixth Assessment Report Working Group III [AR6 WGIII; 2022]) are laid out in columns against the equivalent scenarios within the full RCP scheme, which are laid out in rows; some of the RCP scenarios (RCP1.9, RCP3.4 and RCP7) were added after the original 2014 publication of the RCP system. (The RCPs were the most commonly applied scenarios used in papers that met this review's inclusion criteria.) Further explanations of all of these scenario categories are included in Box 1.^81–83^

Papers were grouped and synthesised by whether they (i) explored the effect of climate change on malaria and NTDs; or (ii) examined potential amelioration of these effects through climate change mitigation and adaptation. Studies that compared several future climate scenarios of different severities (different representative concentration pathways [RCPs], or different combinations of RCPs and shared socioeconomic pathways, for example; please see Box 1 and Figure 1) were defined as projections of the effect of mitigation.

Box 1. Climate change scenarios.

The Intergovernmental Panel on Climate Change (IPCC) has periodically published assessment reports on the scientific understanding of climate change, its projected effects and potential response strategies. As a part of these reports, the IPCC outlines future climate scenarios or pathways based on projections from Global Climate Models, for use in modelling. Papers meeting the inclusion criteria for this review used a variety of different future scenarios, so their outcomes and conclusions generally cannot be directly compared. Here, we summarise the history of IPCC climate scenarios, explain their interpretation and note how they relate to each other (Figure 1).

**IP92**–the first global climate change scenarios, published by IPCC in 1992. These focused on projected future emissions of greenhouse gases (GHGs).^78^

**Special Report on Emission Scenarios** (SRES)—released in 2000 and used in the IPCC's Third Assessment Report (TAR) and Fourth Assessment Report (AR4). The SRES covers a wide range of the main drivers of climate change, from demographic to technological and economic developments, and includes all relevant GHGs and sulphur, and their driving forces.^79^ Four main narrative storylines with different contributions of drivers cover a range of possible outcomes:

A1: Rapid economic growth; population peaks mid-century then declines.

A2: Regionalised world; high population growth.

B1: Global sustainability focus; population peaks mid-century then declines.

B2: Local solutions emphasised; slow population growth.

**Representative Concentration Pathways** (RCPs)—Developed to replace the SRES, for use in climate models as part of the Fifth Assessment Report (AR5) of the IPCC in 2014. Rather than being based on storylines, RCPs describe possible GHG emissions and concentration levels in the atmosphere by 2100, ranging from very low (RCP2.6, radiative forcing peaks at 3 Wm^−2^ and then declines to 2.6 Wm^−2^ by 2100, also known as RCP3PD) to intermediate (RCP4.5 and RCP6.0, radiative forcing is stabilised at approximately 4.5 or 6.0 Wm^−2^), to very high (RCP8.5, radiative forcing continues to increase after 2100). RCPs use time series of emissions and concentrations of the full suite of GHGs, aerosols and chemically active gases, as well as projected land use/land cover and population growth. Compared with the SRES, RCP2.6 has no equivalent; RCP4.5 is similar to SRES B1 but median temperatures rise more quickly in RCP4.5 than in B1 in the first half of the twenty-first century, and then more slowly in the second half; RCP6.0 is similar to SRES B2, with median temperatures rising more quickly in RCP6.0 than in B2 from 2060–2090, but otherwise more slowly; and RCP8.5 is similar to SRES A1FI (A1 fossil-fuel intensive scenario), with median temperatures rising more slowly in RCP8.5 than in A1FI during 2035–2080, but more quickly during other periods.^80,81^

**Shared Socioeconomic Pathways** (SSPs)—These scenarios were introduced in the IPCC's Sixth Assessment Report (AR6) published in 2021. SSPs are integrated scenarios that combine socioeconomic narratives with emission pathways, describing different plausible futures of societal development based on varying assumptions about demographic, economic, social and technological factors. Briefly, these include:

SSP1 (Sustainability): represents a future in which there is rapid economic growth, low population growth and widespread adoption of environmentally friendly technologies. It is also called ‘Taking the Green Road’ and emphasises sustainable development, international cooperation and a focus on environmental stewardship.

SSP2 (Middle of the Road): represents a future in which trends continue along historic trajectories with moderate economic growth, intermediate population growth and technological progress occurring at a moderate pace. It assumes some improvements in living standards and governance but also incorporates challenges and disparities.

SSP3 (Regional Rivalry): represents a future characterised by high population growth, slow economic development and fragmented governance. Also termed ‘A Rocky Road’, it envisions regions prioritising national interests over global cooperation, resulting in regional rivalries, conflicts and limited efforts to address climate change.

SSP4 (Inequality): represents a future marked by high population growth, slow economic development and high income inequality. It assumes limited international cooperation and emphasises national interests and security concerns over environmental issues. Also termed ‘A Road Divided’, this scenario suggests challenges in achieving sustainable development and addressing climate change due to social disparities.

SSP5 (Fossil-Fuelled Development): represents a future with high population growth, rapid economic development and reliance on fossil fuels. Also termed ‘Taking the Highway’, it envisions limited environmental regulations and technological innovation, leading to high GHG emissions and significant impact from climate change.^82^

**AR6 WGIII**—scenario categories C1-C8 (published in 2022 by the IPCC's Sixth Assessment Report AR6 Working Group III) refer to potential mitigation pathways used to reduce GHG emissions and limit global warming. These scenarios relate to warming levels in the twenty-first century:

C1 (1.5°C, no/limited overshoot). Limits warming to 1.5°C by 2100 (with >50% likelihood). Roughly corresponds to SSP1-1.9 (very low).

C2 (1.5°C, high overshoot). After a high overshoot, warming returns to 1.5°C by 2100 (with >50% likelihood). Roughly corresponds to SSP1-2.6 (low).

C3 (Likely below 2°C). Limits warming to 2°C by 2100 (with >67% likelihood).

C4 (Below 2°C). Limits warming to 2°C by 2100 (with >50% likelihood).

C5 (Below 2.5°C). Limits warming to 2.5°C by 2100 (with >50% likelihood).

C6 (Below 3°C). Limits warming to 3°C by 2100 (with >50% likelihood). Roughly corresponds to SSP2-4.5 (intermediate).

C7 (Below 4°C). Limits warming to 4°C by 2100 (with >50% likelihood). Roughly corresponds to SSP3-7.0 (high).

C8 (Above 4°C). Exceeds warming of 4°C by 2100 (with >50% likelihood). Roughly corresponds to SSP5-8.5 (very high).^83^

Based on the volume of literature meeting the inclusion criteria, papers were further grouped by disease into malaria, dengue and chikungunya, other vector-borne NTDs and other non-vector-borne NTDs. Dengue and chikungunya are bracketed as an NTD group in the 2021–2030 NTD road map.^13^ We categorised as ‘vector-borne NTDs’ those for which vector control was a recommended control strategy in the 2021–2030 NTD road map.^13^

We obtained data on the burden of malaria and NTDs by country, quantified as disability-adjusted life years (DALYs, the cumulative number of years lost by a defined population due to illness, disability and death from a particular cause or group of causes), from the 2019 Global Burden of Disease study.^14^ To account for different national levels of health service delivery, we used the healthcare access and quality index (HAQI). This employs data on 32 causes of death from which mortality would be avoidable in the presence of effective healthcare; a high index is a marker of better healthcare. HAQIs were obtained from the 2019 Global Burden of Disease data.^15^ To account for countries’ exposure, sensitivity and ability to adapt to the negative impacts of climate change, we used the 2021 vulnerability score from the Notre Dame Global Adaptation Initiative (ND-GAIN; available at: https://gain-new.crc.nd.edu/ranking/vulnerability). The ND-GAIN measures vulnerability by considering six life-supporting sectors: food, water, health, ecosystem service, human habitat and infrastructure. Countries ranked higher in the index are those that are less vulnerable. After removing papers that examined outcomes at the global level, local polynomial regression was undertaken to predict the number of disease group-specific publications relevant to each country as a function of the country's (i) DALYs for that group of diseases, (ii) HAQI and (iii) climate vulnerability score. As there were <1000 datapoints available for each regression, we used locally estimated scatterplot smoothing (LOESS) for these analyses.

## Results

**Figure 2.**
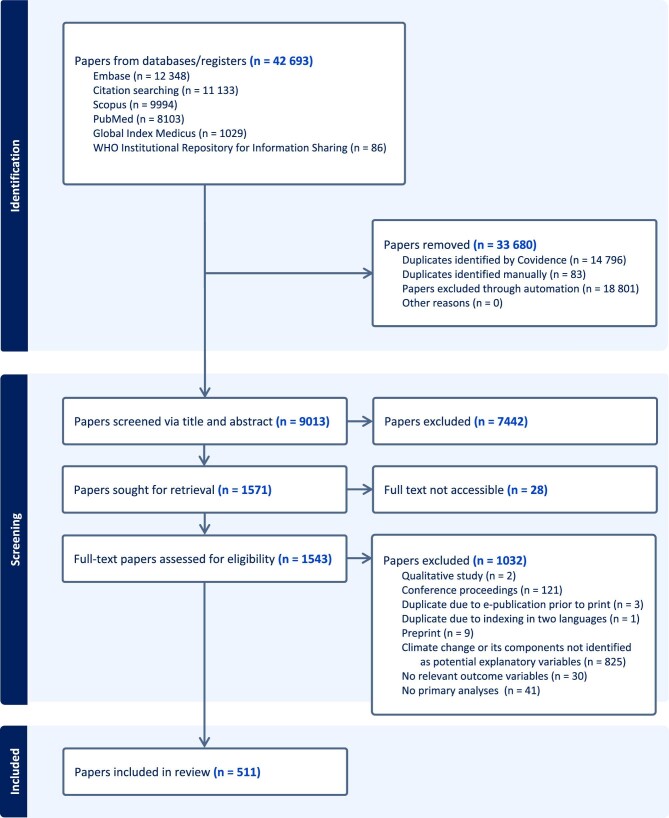
Records included and excluded at each review stage.

Database searches yielded 31 560 records. Forward and backward citation searching yielded an additional 11 133 (of which 8587 were within the specified publication range), producing a combined total of 42 693 records (40 147 within the specified range). After removal of 14 879 duplicates, 27 814 records were available for title-and-abstract screening. The stopping rules for title-and-abstract screening were met after 9013 records had been examined; of these 9013, 7442 (83%) were excluded on the basis that their titles and abstracts indicated a failure to meet the inclusion criteria. Full-text papers were therefore sought for 1571 records (17% of 9013), of which 1543 (98%) were able to be retrieved. Following full-text review, 511 papers (33% of 1543) were included for data extraction; these are summarised in Supplementary Table 2. Extracted data are presented in full in Supplementary Table 3. A flow diagram is included as Figure 2.

**Figure 3.**
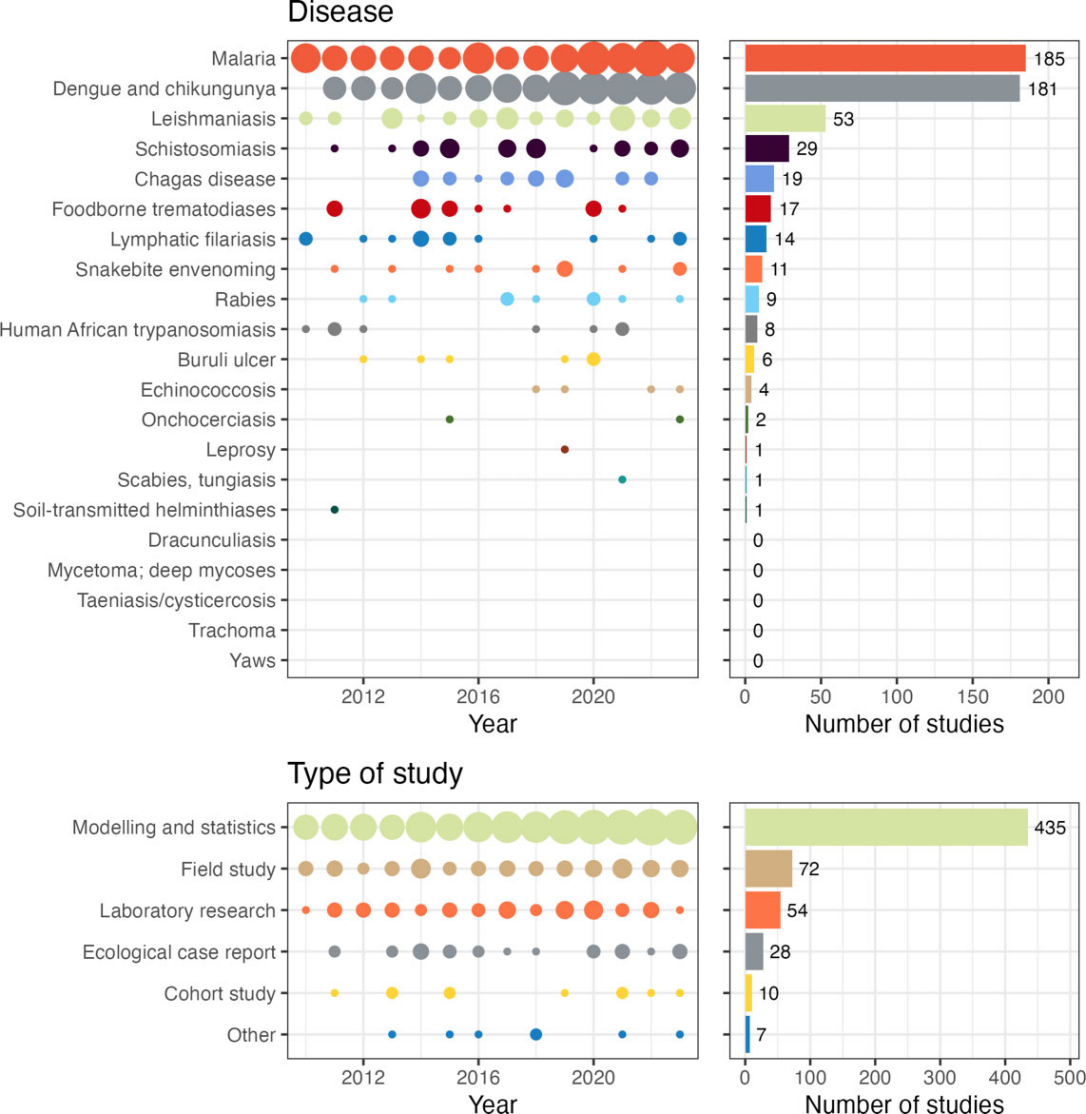
Number of papers by year and total number of papers meeting the inclusion criteria for each (A) disease or disease group, and (B) type of study.

Papers that met the inclusion criteria considered outcomes relevant to malaria (185 papers), dengue and chikungunya (181), the leishmaniases (53), schistosomiasis (29), Chagas disease (19), foodborne trematodiases (17), lymphatic filariasis (14), snakebite envenoming (11), rabies (9), human African trypanosomiasis (8), Buruli ulcer (6), echinococcosis (4), onchocerciasis (2), leprosy (1), scabies (1) and soil-transmitted helminthiases (1). No papers meeting the inclusion criteria considered outcomes relevant to dracunculiasis; mycetoma, chromoblastomycosis or other deep mycoses; taeniasis/cysticercosis; trachoma; or yaws (Figure 3A).

The vast majority of papers used modelling to study the effect of climate change (435 papers). There were 72 field study papers, 54 laboratory research reports, 28 ecological case reports, 10 cohort studies and seven other types of study (Figure 3B; multiple study types were possible in a single paper). There was little variation in the numbers of papers from year to year (Figure 3A and B, left plots).

Among papers meeting the inclusion criteria, a total of 174 (35%) considered the possible ameliorating effect of climate change mitigation for any outcome, 24 (5%) considered adaption strategies and two considered both. Sixty-nine papers considered mitigation in relation to malaria outcomes. Nine explicitly considered adaptation strategies for malaria outcomes. Fifty-three papers addressed climate change mitigation and nine considered adaptation strategies in relation to dengue and chikungunya; four of the papers, including mitigation analyses for dengue and chikungunya, also considered malaria outcomes in the light of climate change mitigation. Sixty other papers addressed climate change mitigation or adaptation with respect to NTD outcomes.

**Figure 4.**
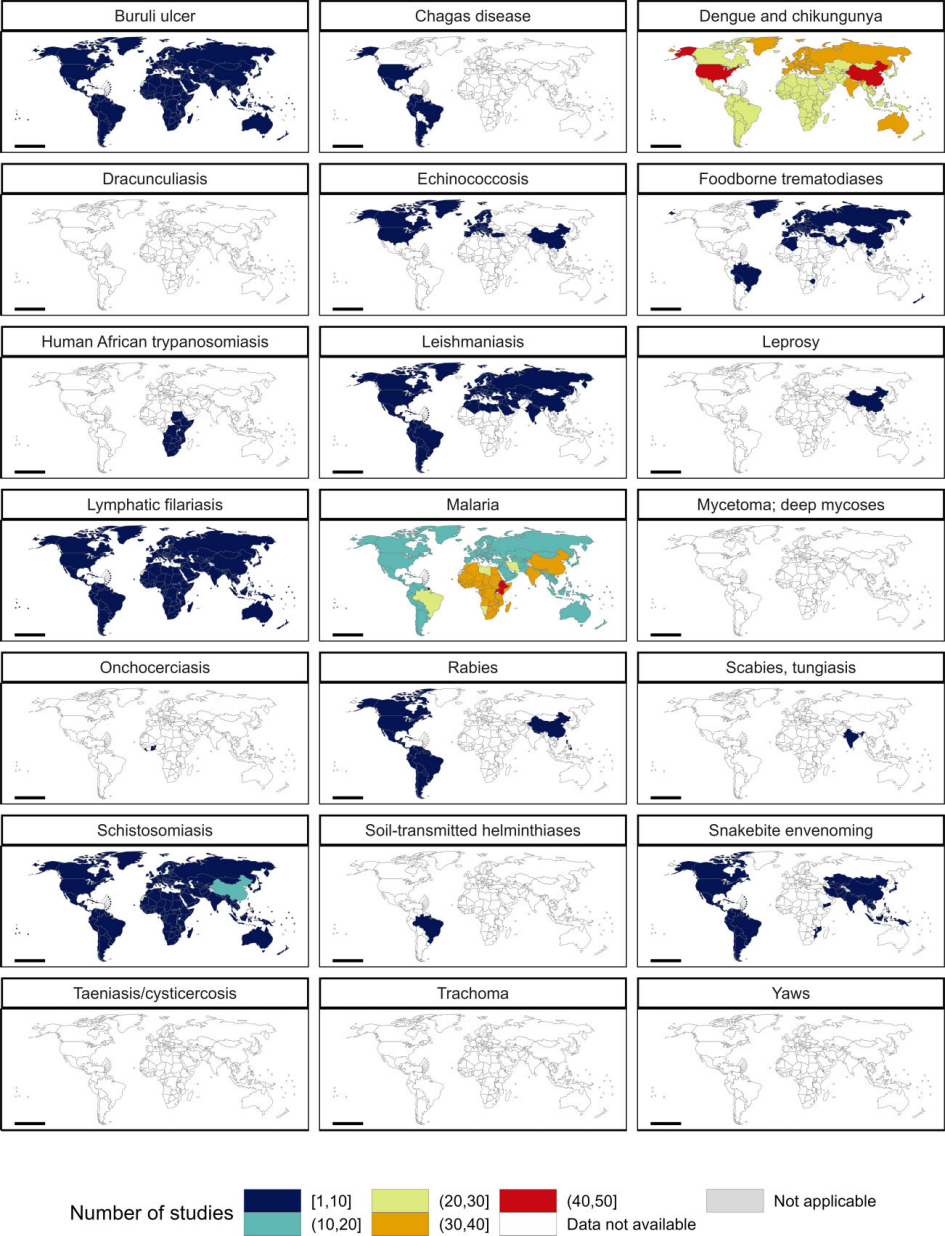
Geographical coverage of papers by disease. Colours represent the total number of papers that met the inclusion criteria, per disease, across countries.

There was wide variation in the geographical coverage of papers by disease (Figure 4). Apart from malaria, dengue and chikungunya, and schistosomiasis, there were <10 papers per disease per country for every country, with many endemic countries not featured in any papers for several NTDs. We also observed distinct distribution patterns for malaria, dengue and chikungunya, and schistosomiasis. Most papers examining the impact of climate change on malaria focused on countries in Africa, Brazil, China or India. Dengue and chikungunya papers were focused on Australia, China, India, countries in Europe and the USA, many of which are countries where these diseases may spread in future years. Several schistosomiasis papers focused on China. For leishmaniasis, papers meeting our inclusion criteria considered countries that were widely distributed around the globe, with the exception of countries in East Africa.

The observed variation in geographical coverage of papers across diseases prompted us to further investigate the links between study location, number of papers, disease burden and country vulnerability to climate change. Given the discrepancy in the number of papers between diseases, we grouped these into four categories: (i) malaria; (ii) dengue and chikungunya; (iii) other vector-borne NTDs (Chagas disease, dracunculiasis, human African trypanosomiasis, leishmaniasis, lymphatic filariasis, onchocerciasis, schistosomiasis, trachoma); and (iv) non-vector-borne NTDs (Buruli ulcer, echinococcosis, foodborne trematodiases, leprosy, rabies, scabies/tungiasis, soil-transmitted helminthiases, snakebite envenoming, taeniasis and cysticercosis).

**Figure 5.**
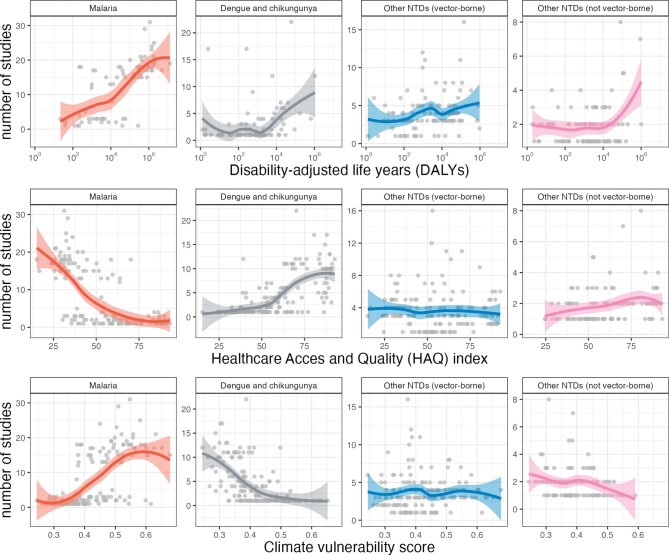
Numbers of papers meeting the inclusion criteria by disease or disease group, compared with country-level (A) DALYs for the disease or disease group; (B) health access and quality index; and (C) climate vulnerability score. Studies with outcomes reported at global level (for all countries) were removed for these analyses. Each circle represents one country; superimposition of multiple circles makes some look darker than others. Lines show locally estimated scatterplot smoothing (LOESS)-generated local polynomial regression.

Our analysis showed different patterns across groups of diseases. For malaria there were clear trends towards more papers covering countries with a high malaria DALY burden, low HAQI and high vulnerability to climate change (Figure 5, first column). For dengue and chikungunya, there was a trend towards increasing numbers of papers covering areas with high burden, but at low DALY burden there was an increase in papers due to the relatively large number of studies looking at potential expansion of these diseases into new areas (Figure 5, second column). This also meant that there was a relative abundance of papers studying these diseases in areas where there is good access to healthcare, and where the climate vulnerability score is low. For the remaining vector-borne NTDs (Figure 5, third column), there was a suggestion of an increasing numbers of papers addressing countries with increasing burden and decreasing HAQI, but no suggestion that analyses were more commonly focused on areas with high climate vulnerability. For the non-vector-borne NTD group, papers more frequently considered countries with high DALY burden for that disease, high HAQI and low climate vulnerability (Figure 5, fourth column).

Detailed analysis of the full text of papers meeting the inclusion criteria revealed additional insights. For malaria, the consensus among papers that met the inclusion criteria was that global warming will extend the area in which transmission is possible to some areas where it was previously too cold for vector or parasite development, while conditions in some currently endemic areas may become too severe to maintain transmission. Zones suitable for transmission may shift both poleward and upwards in altitude. Future expansion could be balanced by more frequent droughts, making environmental conditions unsuitable for transmission in some previously endemic areas, including parts of the Sahel.^16^ Other papers concluded that malaria transmission seasons will last longer, as more months of the year will have a suitable climate.^17^ While these changes could place a greater proportion of the global population at risk,^18^ the potential net impact of climate change on the global burden of malaria remains unclear;^19–22^ papers that met the inclusion criteria were vastly different and difficult to compare with each other. Making predictions for regional and global populations was previously difficult because of a general lack of high-resolution monthly incidence data.^23^ There is likely to be small-scale heterogeneity in effects on the ground.

One aspect of climate change that is already impacting malaria transmission is the increasing frequency of extreme weather events. Severe flooding in Pakistan in 2022 and cyclones in Mozambique and Madagascar in 2023 were accompanied by local spikes in malaria cases, driven by breeding of *Anopheles* mosquitos in flood waters.^24^

For dengue and chikungunya, a significant proportion of papers that met our inclusion criteria described subnational studies with subdecadal time frames. Models with broader geographic and temporal boundaries predict dramatic expansion in the future range of relevant *Aedes* vector species,^25–27^ in line with trends that are already being observed.^28–30^ Some local retreat from geographies currently occupied by *Aedes* spp. is also predicted.^31^ Alongside the overall increase in vector range, considerable increases are projected in the number of future dengue cases under more adverse climate change scenarios (Box 1) in some models.^30,32^ Other models predict a plateauing of dengue in highly endemic regions by 2050.^28^ There is undoubtedly uncertainty about the magnitude and direction of future geographic expansion.

For the leishmaniases, 53 papers that met the inclusion criteria considered relevant outcomes. However, there are many different pathogens and vector species contributing to this complex of diseases (Table 1), making a reliable, complete picture very difficult to draw. Several large-scale models predict changes in the range of relevant sandfly species around the Mediterranean and in the Americas, with transmission in the latter extending as far north as southern Canada in some future climate scenarios.^33–37^ The number of people in North America living in areas in which leishmaniasis is transmitted could double from 2010 to 2080 under SRES B2.^37^ The paucity of information on the potential impact of climate change on leishmaniasis in Africa (Figure 4) was striking.

For schistosomiasis, one model predicted increased prevalence and intensity of infection in some areas of East Africa over the next few decades, particularly in Rwanda, Burundi, south-west Kenya and eastern Zambia, with concurrent substantial decreases in risk in parts of Kenya, southern South Sudan and eastern Democratic Republic of the Congo.^38^ Decreases in transmission are predicted for China,^39^ although the possibility was also invoked that disease will re-emerge in parts of mainland China where it was previously eliminated.^40^ This concern may account for the relative concentration of publications that focused on China (Figure 4).

There were few papers on the remaining NTDs, with a bias towards vector-borne diseases, possibly due to the existence of established methodologies for examining vector suitability and its links to climate. Climate impacts on these other NTDs, whether through environmental changes or societal ones, such as changes in time to diagnosis due to changes in access to health systems,^41^ remain largely unexplored.

## Discussion

For thousands of years, societies have been shaped and reshaped by epidemics, with Ebola virus disease, COVID-19 and mpox presenting only the most recent striking examples. A distinct feature of the climate crisis is the pace of change in underlying global ecosystems. This generates uncertainty about the future epidemiology of multiple diseases: not only those that have historically manifested as epidemics, but also those formerly considered as stable and endemic, and those being driven towards elimination or eradication. Climate change will simultaneously reshape the epidemiology of many non-infectious diseases, threaten health infrastructure, affect the health workforce and alter other foundational determinants of human health. These parallel effects will exacerbate the challenge presented by the evolving epidemiology of infectious diseases.^42,43^

Malaria and many NTDs have relatively complex life cycles, involving overlapping webs of interactions between humans and vertebrate and invertebrate animals. Multiple points of exposure to ecological, biological and social systems increase the probability that climate change will alter disease incidence or prevalence.^43,44^ This environmental sensitivity makes prediction of future scenarios both difficult and important. The perceived difficulty is borne out in the literature identified here: for most of the outcomes in scope, projections of the effects of future climate change at large scale are scarce. Projections that do exist incorporate considerable uncertainty.

Of all potential outcomes framed for this review, those related to malaria, dengue and chikungunya, and the leishmaniases, were the most studied. Yet even for malaria, long-term projections of future transmission scenarios remain inadequate for robust planning. Beyond the expected short-term effects of extreme weather events on local disease incidence,^45^ it is difficult to be definitive^46^–global incidence and attributable deaths may go up, down or stay about the same, depending on multiple factors, including the success or otherwise of nascent vaccination programmes.^6^ The arboviral NTDs, dengue and chikungunya, on the other hand, are generally predicted to continue their current surge.^28,29^ Global expansion in the population at risk of the leishmaniases seems likely. The predicted effect of climate change on these diseases suggests that effects on vectors of other vector-borne NTDs will be similarly important to understand.

For many of the NTDs that we grouped as ‘non-vector-borne’, however, limitations in our capacity for prediction stem in part from gaping deficiencies in our understanding of disease transmission at steady state. Data published only after the searches were conducted for this review pin responsibility for *Mycobacterium ulcerans* transmission in southeastern Australia on the mosquito *Aedes notoscriptus*,^47^ whereas transmission by mosquitos was previously hypothesised but unproven, and analogous work has not yet been published for Buruli ulcer-endemic areas of the Americas, Japan, Papua New Guinea or West Africa. Postulated mechanisms for the transmission of leprosy are still based on circumstantial evidence.^13^ The nematode worm *Dracunculus medinensis*, the target of a global eradication programme that began in 1980, was only relatively recently discovered to infect paratenic and definitive hosts other than humans.^48^ These gaps in our knowledge have lingered because funding for research on NTDs is thin^49^ and spread across a very large number of pathogens (Table 1), some of which are probably relatively rare.^50,51^

Multiplying that disease-specific uncertainty are uncertainties surrounding future climate scenarios and their secondary impacts (including, for example, on conflict, migration and demography), which are further clouded by our joint hope that individual and collective behaviours will change sufficiently to allow greenhouse gas concentrations to fall and thereby effectively mitigate climate change. This uncertainty for virtually every parameter of NTD transmission models^52^ makes decadal projections of future NTD prevalence or incidence feel ill-advised. Unfortunately, without those projections, NTDs are likely to continue to be given very limited attention in climate-related discussions, including, for example, in the Assessment Reports of the Intergovernmental Panel on Climate Change.^53^ Better understanding of NTD transmission dynamics, and estimates—even if heavily caveated—of the potential impacts of climate change, are precisely what is needed now.

The current work builds on previously published reviews,^12,43,54^ but continues to have some limitations. First, although we undertook screening of titles and abstracts by two independent observers, our use of tags to highlight certain characteristics of papers within the Covidence platform may have unmasked some second screeners to the first screener's decision.

Second, screening for mitigation and adaptation strategies was challenging; the process required considerable judgement. It is possible that relevant sources were overlooked in the >42 000 records that our searches identified.

Third, we did not critically quality-appraise the methodology used for each included paper (e.g. number, size and origin of datasets; appropriateness of climate models, such as use of downscaling; and inclusion of all potentially relevant variables). Many studies had low power, did not consider all potential explanatory variables or confounders, or were otherwise methodologically weak. To be useful as a summary of coverage within the published literature, our visualisations imply that all studies contribute equally to the evidence base, whereas they do not.

Fourth, under-ascertainment is an issue for many diseases, but is particularly problematic for malaria and NTDs; their concentration in impoverished populations means that patients with these diseases have HAQIs that are far from ideal. Under-ascertainment is a specifically identified issue for dengue, in which second infections may be considerably more likely to produce disease that leads to clinical presentation and therefore registration.^55^

Fifth, to be included in this review, a paper had to explicitly juxtapose climate change and relevant outcomes. Some authors may have made tenuous arguments linking weather-related variables (such as temperature or rainfall) to climate change, resulting in inclusion; others may not have been explicit in framing climate change implications when doing so would have been justifiable.

Sixth, it is likely that source data were used more than once in groups of papers with the same or related outcome measures for overlapping geographies and overlapping timespans.

Seventh, we did not specifically look for the impact of climate change mediated through internal displacement and migration of people;^56^ changes in institutional capacity and service provision;^57,58^ vector microbiome, genetics or gene expression; or pathogen genetics or gene expression.^59,60^ All of these mechanisms may be important.

Eighth, as for any review, our searches had a fixed date range and were not exhaustive within that range. Not all possible intermediate and reservoir hosts (e.g. for rabies) were specifically included. Insect species with postulated but unproven vectorial capacity were excluded; *Culex pipiens* is a known vector of lymphatic filariasis in Egypt but papers considering it in other contexts were set aside. Studies in press were excluded by design; we were aware of forthcoming work on the impact of climate change on several diseases that had been submitted for peer review but were not yet published when our searches closed. The December 2023 addition of noma to the WHO's list of NTDs^61^ occurred too late for noma to be included in our searches.

Ninth, the high degree of heterogeneity (in questions examined, methods used and so on) precluded quantitative synthesis.

Tenth, projecting disease burdens forward over long timescales means that future changes in treatment and control strategies would ideally also be taken into account. This is difficult to do. Authors of studies from the early part of our 2010–2023 publication window may not have foreseen the scale-up in intervention coverage that has occurred for many diseases in the past 10–15 y.

Despite uncertainty around data, some general conclusions and recommendations are proposed here. It can be inferred from existing data, first, that climate change is likely to have profound direct and indirect implications for malaria, dengue and chikungunya, leishmaniasis and at least several other vector-borne NTDs, even if the amplitude and direction of the effects will probably vary by disease and location, be non-linear^22,62–64^ and evolve with time. Changes of two kinds will be apparent: diseases will move around, and where endemicity is constant, there will be local increases or decreases in incidence or prevalence. There is a pressing need to safeguard previous global health gains by scaling up proven interventions and achieving impact before future changes render those interventions ineffective.^65^ Second, the lack of predictability, even over relatively short timescales, calls for existing surveillance and intervention systems to be reinforced and regularly reviewed. Integrated surveillance and intervention systems, covering multiple diseases^66^ and taking a One Health approach,^67^ could offer efficiencies. Third, communities should be consulted and involved in these reviews of surveillance and intervention systems, and in research undertaken at the interface of infectious diseases and climate change, to maximise the relevance of such efforts despite changing human populations. Fourth, integrating climate resilience into health systems is critical. This should encompass investing in health infrastructure, fostering cross-sector collaboration, adapting to the needs of displaced populations, improving access to health products and accelerating research and development to fill known gaps.^68^ A particular requirement is access to existing and new countermeasures to limit future expansions in disease burden. Fifth, we do not know enough.

Box 2. Recommendations for future research.

Research to fill current knowledge gaps on the likely impacts of climate change, mitigation and adaptation strategies on malaria and NTDs should:

1. where projections are modelled, be based on clearly defined climate scenarios, and include multiple scenarios, ideally using the most recent categories defined by the IPCC, in order to facilitate comparisons across studies, diseases and geographies.
2. where projections are modelled, incorporate not only climate scenarios but also sociodemographic and population density projections. This may require developments in methodology to ensure that demographic transitions underpinning the epidemiological models are in line with those assumed in the projections.
3. where projections are modelled, ideally incorporate detailed analyses of the likely impact of climate change mitigation and adaptation strategies, which are currently rare, and the modelled effectiveness of existing and new interventions (vector control, vaccines, treatments) under multiple climate scenarios.
4. explore the potential impacts of climate change on a broader set of NTDs and geographies, prioritising places with the highest disease burdens and the people most vulnerable to the future impacts of climate change.
5. recognise that, because of the paucity of data, it will remain challenging to estimate the impact of climate change and other secular trends on NTDs, and to anticipate potential interactions between climate change and the impact of interventions. Therefore, new methodologies are needed, based on plausible biological assumptions. This will require distinct study types to prepare for modelling, including prospective population-based investigations, laboratory studies, biological experiments (e.g. mosquito or egg survival) and social science that can be performed in suitable locations to inform projections and provide a data source for future estimates of change. Investigating methods to extrapolate laboratory findings to field settings would be beneficial.
6. prioritise standardisation and collaboration, including across disciplines. Specifically for modelling, we propose the development and adoption of standardised frameworks for future projections, using, where possible, standardised survey or case data, and an open collaborative model in which source data and contributions to code can be tracked, to speed up and unify research while protecting the rights of countries that generate primary data and acknowledging all collaborators.
7. facilitate leadership of scientists in affected areas to undertake and communicate research and its implications. An understanding of local context and closer relationships with stakeholders will lead to higher quality analyses with increased uptake in local decision-making processes.
8. where projections are developed, provide actionable data for policymaking at national and subnational levels. For example, studies focusing on climate-driven dengue expansion to new locations (both in high-income and low- and middle-income countries) should investigate appropriate methods of surveillance, which could be targeted at high-risk areas in a cost-efficient manner. (Risk here could be interpreted in multiple ways: relating to the potential for increases in vector abundance, infection, severe disease or outcomes such as lost gross domestic product (GDP), for example.)

Based on our analysis, we also propose several key recommendations to guide future studies (Box 2). The most important of these is for standardisation and collaboration. Our scope included 21 diseases and disease groups, at least 76 distinct pathogens, 373 venomous snakes and humans everywhere; there is insufficient modelling capacity globally for this to be investigated on a competitive basis, particularly when the appropriate modelling methodologies change with the level of endemicity and results are needed at multiple scales. Indeed, our analysis suggests that existing studies may not be sufficiently focused on areas where the need to plan for adaptation may be greatest. We recommend holistic approaches to risk assessment, incorporating more of the available data and recruiting more of the available brainpower to undertake ensemble analyses with agreed best-practice methodology. Modelling efforts should incorporate consideration of humans, pathogens, vectors, intermediate and reservoir hosts and the effect of relevant interventions, as appropriate, to generate predictions over decadal time frames—and not ignore populations where these diseases are currently endemic. Collectively, these measures should reduce potential duplication and hopefully produce more complete and more accurate estimates of future vulnerability, exposure and impact. Open-source collaborative modelling platforms^17,23^ could facilitate contributions from as many relevant stakeholders as are willing to engage, and allow tailoring of consistently high-quality outputs for specific audiences. Collaboration could include involvement of affected communities through citizen science: in vector surveillance, for example. Accessible global databases on disease and vector occurrence^69–75^ should be harnessed and adapted to cover additional diseases. Broader use of remotely sensed climate data should be explored, particularly where locally acquired data are unavailable or microclimates are of relevance. Long-term time-series data should be pooled and re-analysed to tease out the relative contributions of deliberate interventions, secular trends, seasonality and climate change. Production of detailed risk and distribution maps should be facilitated to help plan local control and elimination efforts. The fact that many NTDs are targeted for eradication, interruption of transmission or elimination as a public health problem by 2030 should not dissuade us from taking a long-term view of this work;^43^ global health ambitions are not always realised, and the best possible current understanding of counterfactual scenarios should help decision-makers to target resources and chart the most appropriate course.

### Conclusions

It is difficult to have immersed ourselves in this literature as we have without acquiring a deepened sense of foreboding over the adverse influence that we as a species are visiting on our planet and its most vulnerable people. Adverse changes have already occurred in the incidence or prevalence of infectious diseases that cause death or profound morbidity. Women, children, older people, indigenous groups and ethnic minorities, migrants and the very poor have contributed least but are likely to experience most of the effects of the climate crisis,^76^ notably including through any increase in the burden of malaria or NTDs. An emerging opportunity to correct this inequity arises through financial commitments to NTD control and elimination made at the 28th United Nations Climate Change Conference in December 2023.^77^ Allocation of these resources should be guided by informed scenario analyses of current and future disease burden. The work described in this review is a start; convening stakeholders globally to advance the research agenda must be our next collective move.

## Supplementary Material

trae026_Supplemental_Files

## Data Availability

All data relevant to the study are available in Supplementary Table 2 and Supplementary Table 3. Code available upon request.

## Disclaimers

The authors alone are responsible for the views expressed in this article and they do not necessarily represent the views, decisions or policies of the institutions with which they are affiliated. The boundaries and names shown and the designations used on the maps in this article do not imply the expression of any opinion whatsoever on the part of the authors, or the institutions with which they are affiliated, concerning the legal status of any country, territory, city or area or of its authorities, or concerning the delimitation of its frontiers or boundaries.
