# Supplementary material for: Climate change, malaria and neglected tropical diseases: a scoping review
Source: Trans R Soc Trop Med Hyg. 2024 May 10;118(9):561–79. doi: 10.1093/trstmh/trae026 (PMC11367761; doi:10.1093/trstmh/trae026)
Supplement: trae026_Supplemental_Files [file trae026_supplemental_files.zip › supplementary-material-search-strings.docx]

**Search strings**

**Embase Ovid, NTDs - searching database “1996 to 2023 week 40”** (8485 results)

(("Buruli Ulcer" or "ulcerans" or "possum" or "peregrinus" or "vulpecula" or "cunninghami" or "Chagas disease" or "cruzi" or "kissing bug*" or "Triatoma infestans" or "Rhodnius prolixus" or "Triatoma dimidiate" or "Panstrongylus megistus" or "triatom*" or dengue or chikungunya or "Aedes aegypti" or "Aedes albopictus" or "Aedes polynesiensis" or "Aedes scutellaris" or "Aedes furcifer-taylori" or "Aedes africanus" or "Aedes luteocephalus" or "Asian tiger mosquito" or dracunculiasis or "guinea-worm" or "Dracunculus medinensis" or "Cyclops" or "Cystic echinococcosis" or "hydatid" or "Alveolar echinococcosis" or "unicystic echinococcosis" or "polycystic echinococcosis" or "Echinococcus" or "Foodborne trematod*" or "trematodiasis" or "Clonorchis sinensis" or "Fasciola hepatica" or "Opisthorchis viverrini" or "Paragonimus westermani" or "opisthorchiasis" or "fascioliasis" or "paragonimiasis" or "clonorchiasis" or "Human African trypanosom*" or "sleeping sickness" or "gambiense" or "rhodesiense" or "tsetse" or "Leishmani*" or "aethiopica" or "amazonensis" or "braziliensis" or "Leishmania chagasi" or "L. chagasi" or "colombiensis" or "Leishmania donovani" or "L. donovani" or "garnhami" or "guyanensis" or "Leishmania infantum" or "L. infantum" or "killicki" or "lainsoni" or "lindenbergi" or "Leishmania major" or "L. major" or "mexicana" or "naiffi" or "panamensis" or "peruviana" or "pifanoi" or "Leishmania shawi" or "L. shawi" or "Leishmania tropica" or "L. tropica" or "venezuelensis" or "phlebotomine" or "sandfl*" or "sand fl*" or "Lutzomyia" or "Phlebotomus" or "Leprosy" or "Mycobacterium leprae" or "Mycobacterium lepromatosis" or "Lymphatic filariasis" or "Wuchereria bancrofti" or "Brugia malayi" or "Brugia timori" or Culex or Anopheles or "Mansonia" or Aedes or "Mycetoma" or "actinomycetoma" or "eumycetoma" or "chromoblastomycosis" or "paracoccidioidomycosis" or "sporotrichosis" or "deep mycos*" or "Paracoccidioides lutzii" or "Fonsecaea pedrosoi" or "Cladophialophora carrionii" or "Phialophora verrucose" or "Madurella mycetomatis" or "Nocardia brasiliensis" or "Actinomadura madurae" or "Streptomyces somaliensis" or "Actinomadura pelletieri" or "Sporothrix schenckii" or "Onchocerc*" or "river blindness" or blackfl* or "black fl*" or "Simulium" or "Rabies" or "scabies" or "Sarcoptes scabiei" or "jigger flea" or "tungiasis" or "Tunga penetrans" or "chigo*" or "schistosom*" or "bilharzia" or "Bulinus" or "Biomphalaria" or "Oncomelania" or "Robertsiella" or "Neotricula" or "soil transmitted helminth*" or "soil-transmitted helminth*" or "Ascaris lumbricoides" or "roundworm" or "Necator americanus" or "Ancylostoma duodenale" or "hookworm" or "Trichuris trichiura" or "whipworm" or Strongyloid* or "snakebite" or "snake bite" or ( "snake" and "envenom*" ) or "taenia*" or "cysticercosis" or "Taenia solium" "trachoma" or "Chlamydia trachomatis" or "trichiasis" or "Musca sorbens" or "yaws" or "endemic treponematos*" or "pinta" or "bejel" or "nonvenereal endemic syphilis" or "Treponema pallidum pertenue" or "Treponema carateum" or "Treponema pallidum endemicum" or "neglected tropical disease*" or "NTD" or "neglected disease*" or ( ( mosquito-borne or vector-borne ) and disease ) or "neglected disease*").ti,ab,kf. or neglected disease/) and (("global warming" or "temperature increase" or (increas* and temperature) or "temperature rise" or "temperature elevat*" or humidity or rainfall or drought or flood* or "circulation model*" or "climate change" or ( climate and chang* ) or "future climate" or "greenhouse effect" or ( meteorological and ( factor* or variable* )) or precipitation or heatwave or "heat wave" or wildfire or storm* or "sea level" or "land cover" or "extreme weather" or temperature).ti,ab,kf. or climate change/ or greenhouse effect/)

limit to yr="2010–Current"

**Scopus, NTDs** (4706 results)

TITLE-ABS-KEY ( "Buruli Ulcer" OR "ulcerans" OR "possum" OR "peregrinus" OR "vulpecula" OR "cunninghami" OR "Chagas disease" OR "cruzi" OR "kissing bug*" OR "Triatoma infestans" OR "Rhodnius prolixus" OR "Triatoma dimidiate" OR "Panstrongylus megistus" OR "triatom*" OR dengue OR chikungunya OR "Aedes aegypti" OR "Aedes albopictus" OR "Aedes polynesiensis" OR "Aedes scutellaris" OR "Aedes furcifer-taylori" OR "Aedes africanus" OR "Aedes luteocephalus" OR "Asian tiger mosquito" OR dracunculiasis OR "guinea-worm" OR "Dracunculus medinensis" OR "Cyclops" OR "Cystic echinococcosis" OR "hydatid" OR "Alveolar echinococcosis" OR "unicystic echinococcosis" OR "polycystic echinococcosis" OR "Echinococcus" OR "Foodborne trematod*" OR "trematodiasis" OR "Clonorchis sinensis" OR "Fasciola hepatica" OR "Opisthorchis viverrini" OR "Paragonimus westermani" OR "opisthorchiasis" OR "fascioliasis" OR "paragonimiasis" OR "clonorchiasis" OR "Human African trypanosom*" OR "sleeping sickness" OR "gambiense" OR "rhodesiense" OR "tsetse" OR "Leishmani*" OR "aethiopica" OR "amazonensis" OR "braziliensis" OR "Leishmania chagasi" OR "L. chagasi" OR "colombiensis" OR "Leishmania donovani" OR "L. donovani" OR "garnhami" OR "guyanensis" OR "Leishmania infantum" OR "L. infantum" OR "killicki" OR "lainsoni" OR "lindenbergi" OR "Leishmania major" OR "L. major" OR "mexicana" OR "naiffi" OR "panamensis" OR "peruviana" OR "pifanoi" OR "Leishmania shawi" OR "L. shawi" OR "Leishmania tropica" OR "L. tropica" OR "venezuelensis" OR "phlebotomine" OR "sandfl*"OR "sand fl*" OR "Lutzomyia" OR "Phlebotomus" OR "Leprosy" OR "Mycobacterium leprae" OR "Mycobacterium lepromatosis"OR "Lymphatic filariasis" OR "Wuchereria bancrofti" OR "Brugia malayi" OR "Brugia timori" OR culex OR anopheles OR "Mansonia" OR aedes OR "Mycetoma" OR "actinomycetoma" OR "eumycetoma" OR "chromoblastomycosis" OR "paracoccidioidomycosis" OR "sporotrichosis" OR "deep mycos*" OR "Paracoccidioides lutzii" OR "Fonsecaea pedrosoi" OR "Cladophialophora carrionii" OR "Phialophora verrucose" OR "Madurella mycetomatis" OR "Nocardia brasiliensis" OR "Actinomadura madurae" OR "Streptomyces somaliensis" OR "Actinomadura pelletieri" OR "Sporothrix schenckii" OR "Onchocerc*" OR "river blindness" OR blackfl* OR "black fl*" OR "Simulium" OR "Rabies" OR "scabies" OR "Sarcoptes scabiei"OR "jigger flea" OR "tungiasis" OR "Tunga penetrans" OR "chigo*" OR "schistosom*" OR "bilharzia" OR "Bulinus" OR "Biomphalaria" OR "Oncomelania" OR "Robertsiella" OR "Neotricula" OR "soil transmitted helminth*" OR "soil-transmitted helminth*" OR "Ascaris lumbricoides" OR "roundworm" OR "Necator americanus" OR "Ancylostoma duodenale" OR "hookworm"OR "Trichuris trichiura" OR "whipworm" OR strongyloid* OR "snakebite" OR "snake bite" OR ( "snake" AND "envenom*" ) OR "taenia*" OR "cysticercosis" OR "Taenia solium" "trachoma" OR "Chlamydia trachomatis" OR "trichiasis" OR "Musca sorbens" OR "yaws" OR "endemic treponematos*" OR "pinta" OR "bejel" OR "nonvenereal endemic syphilis" OR "Treponema pallidum pertenue" OR "Treponema carateum" OR "Treponema pallidum endemicum" OR "neglected tropical disease*" OR "NTD" OR "neglected disease*" OR ( ( mosquito-borne OR vector-borne ) AND disease ) ) AND ( "global warming" OR "temperature increase" OR ( increas* AND temperature ) OR "temperature rise" OR "temperature elevat*" OR humidity OR rainfall OR drought OR flood* OR "circulation model*" OR "climate change" OR ( climate AND chang* ) OR "future climate" OR "greenhouse effect" OR ( meteorological AND ( factor* OR variable* ) ) OR precipitation OR heatwave OR "heat wave" OR wildfireOR storm* OR "sea level" OR "land cover" OR "extreme weather" OR temperature ) AND PUBYEAR > 2009

**PubMed, NTDs**(5341 results)

("Buruli Ulcer"[tiab] OR "ulcerans"[tiab] OR "possum"[tiab] OR "peregrinus"[tiab] OR "vulpecula"[tiab] OR "cunninghami"[tiab] OR "Chagas disease"[tiab] OR "cruzi"[tiab] OR "kissing bug*"[tiab] OR "Triatoma infestans"[tiab] OR "Rhodnius prolixus"[tiab] OR "Triatoma dimidiate"[tiab] OR "Panstrongylus megistus"[tiab] OR "triatom*"[tiab] OR dengue[tiab] OR chikungunya[tiab] OR "Aedes aegypti"[tiab] OR "Aedes albopictus"[tiab] OR "Aedes polynesiensis"[tiab] OR "Aedes scutellaris"[tiab] OR "Aedes furcifer-taylori"[tiab] OR "Aedes africanus"[tiab] OR "Aedes luteocephalus"[tiab] OR "Asian tiger mosquito"[tiab] OR dracunculiasis[tiab] OR "guinea-worm"[tiab] OR "Dracunculus medinensis"[tiab] OR "Cyclops"[tiab] OR "Cystic echinococcosis"[tiab] OR "hydatid"[tiab] OR "Alveolar echinococcosis"[tiab] OR "unicystic echinococcosis"[tiab] OR "polycystic echinococcosis"[tiab] OR "Echinococcus" "Foodborne trematod*"[tiab] OR "trematodiasis"[tiab] OR "Clonorchis sinensis"[tiab] OR "Fasciola hepatica"[tiab] OR "Opisthorchis viverrini"[tiab] OR "Paragonimus westermani"[tiab] OR "opisthorchiasis"[tiab] OR "fascioliasis"[tiab] OR "paragonimiasis"[tiab] OR "clonorchiasis"[tiab] OR "Human African trypanosom*"[tiab] OR "sleeping sickness"[tiab] OR "gambiense"[tiab] OR "rhodesiense"[tiab] OR "tsetse"[tiab] OR "Leishmani*"[tiab] OR "aethiopica"[tiab] OR "amazonensis"[tiab] OR "braziliensis"[tiab] OR "Leishmania chagasi"[tiab] OR "L. chagasi"[tiab] OR "colombiensis"[tiab] OR "Leishmania donovani"[tiab] OR "L. donovani"[tiab] OR "garnhami"[tiab] OR "guyanensis"[tiab] OR "Leishmania infantum"[tiab] OR "L. infantum"[tiab] OR "killicki"[tiab] OR "lainsoni"[tiab] OR "lindenbergi"[tiab] OR "Leishmania major"[tiab] OR "L. major"[tiab] OR "mexicana"[tiab] OR "naiffi"[tiab] OR "panamensis"[tiab] OR "peruviana"[tiab] OR "pifanoi"[tiab] OR "Leishmania shawi"[tiab] OR "L. shawi"[tiab] OR "Leishmania tropica"[tiab] OR "L. tropica"[tiab] OR "venezuelensis"[tiab] OR "phlebotomine"[tiab] OR "sandfl*"[tiab] OR "sand fl*"[tiab] OR "Lutzomyia"[tiab] OR "Phlebotomus"[tiab] OR "Leprosy"[tiab] OR "Mycobacterium leprae"[tiab] OR "Mycobacterium lepromatosis"[tiab] OR "Lymphatic filariasis"[tiab] OR "Wuchereria bancrofti"[tiab] OR "Brugia malayi"[tiab] OR "Brugia timori"[tiab] OR Culex[tiab] OR Anopheles[tiab] OR "Mansonia"[tiab] OR "Aedes"[tiab] OR "Mycetoma"[tiab] OR "actinomycetoma"[tiab] OR "eumycetoma"[tiab] OR "chromoblastomycosis"[tiab] OR "paracoccidioidomycosis"[tiab] OR "sporotrichosis"[tiab] OR "deep mycos*"[tiab] OR "Paracoccidioides lutzii"[tiab] OR "Fonsecaea pedrosoi"[tiab] OR "Cladophialophora carrionii"[tiab] OR "Phialophora verrucose"[tiab] OR "Madurella mycetomatis"[tiab] OR "Nocardia brasiliensis"[tiab] OR "Actinomadura madurae"[tiab] OR "Streptomyces somaliensis"[tiab] OR "Actinomadura pelletieri"[tiab] OR "Sporothrix schenckii"[tiab] OR "Onchocerc*"[tiab] OR "river blindness"[tiab] OR blackfl* OR "black fl*"[tiab] OR "Simulium"[tiab] OR "Rabies"[tiab] OR "scabies"[tiab] OR "Sarcoptes scabiei"[tiab] OR "jigger flea"[tiab] OR "tungiasis"[tiab] OR "Tunga penetrans"[tiab] OR "chigo*"[tiab] OR "schistosom*"[tiab] OR "bilharzia"[tiab] OR "Bulinus"[tiab] OR "Biomphalaria"[tiab] OR "Oncomelania"[tiab] OR "Robertsiella"[tiab] OR "Neotricula"[tiab] OR "soil transmitted helminth*"[tiab] OR "soil-transmitted helminth*"[tiab] OR "Ascaris lumbricoides"[tiab] OR "roundworm"[tiab] OR "Necator americanus"[tiab] OR "Ancylostoma duodenale"[tiab] OR "hookworm"[tiab] OR "Trichuris trichiura"[tiab] OR "whipworm"[tiab] OR Strongyloid* OR "snakebite"[tiab] OR "snake bite"[tiab] OR ( "snake"[tiab] AND "envenom*"[tiab]) OR "taenia*"[tiab] OR "cysticercosis"[tiab] OR "Taenia solium"[tiab] OR "trachoma"[tiab] OR "Chlamydia trachomatis"[tiab] OR "trichiasis"[tiab] OR "Musca sorbens"[tiab] OR "yaws"[tiab] OR "endemic treponematos*"[tiab] OR "pinta"[tiab] OR "bejel"[tiab] OR "nonvenereal endemic syphilis"[tiab] OR "Treponema pallidum pertenue"[tiab] OR "Treponema carateum"[tiab] OR "Treponema pallidum endemicum"[tiab] OR "neglected tropical disease*"[tiab] OR "NTD"[tiab] OR (( mosquito-borne[tiab] OR vector-borne[tiab] ) AND disease[tiab] )OR neglected diseases[MeSH]) AND ( "global warming"[tiab] OR "temperature increase"[tiab] OR ( increas*[tiab] AND temperature[tiab] ) OR "temperature rise"[tiab] OR "temperature elevat*"[tiab] OR humidity[tiab] OR rainfall[tiab] OR drought[tiab] OR flood*[tiab] OR "circulation model*"[tiab] OR "climate change"[tiab] OR "climate change"[MeSH] OR ( climate[tiab] AND change[tiab] ) OR "future climate"[tiab] OR ( meteorological[tiab] AND ( factor*[tiab] OR variable*[tiab] ) ) OR precipitation[tiab] OR heatwave[tiab] OR "heat wave"[tiab] OR wildfire[tiab] OR storm*[tiab] OR "sea level"[tiab] OR "land cover"[tiab] OR "extreme weather"[tiab] OR temperature[tiab]) AND 2010/01/01:2024/10/06[dp]

**Embase Ovid, malaria** (3826 results)

((malaria* OR black water fever* or blackwater fever* or marsh fever* or falciparum or paludism or plasmodi* or remittent fever* or swamp fever* or vivax or malariae or ovale or knowlesi).ti,ab,kf. or malaria/ ) and (("global warming" or "temperature increase" or (increas* and temperature) or "temperature rise" or "temperature elevat*" or humidity or rainfall or drought or flood* or "circulation model*" or "climate change" or ( climate and chang* ) or "future climate" or "greenhouse effect" or ( meteorological and ( factor* or variable* )) or precipitation or heatwave or "heat wave" or wildfire or storm* or "sea level" or "land cover" or "extreme weather" or temperature).ti,ab,kf. or climate change/ or greenhouse effect/)

**PubMed, malaria** (2429 results)

(malaria*[tiab] OR “black water fever*”[tiab] OR “blackwater fever*”[tiab] OR “marsh fever*”[tiab] OR falciparum[tiab] OR paludism[tiab] OR plasmodi*[tiab] OR “remittent fever*”[tiab] OR “swamp fever*”[tiab] OR vivax[tiab] OR malariae[tiab] OR ovale[tiab] OR knowlesi) AND ( "global warming"[tiab] OR "temperature increase"[tiab] OR ( increas*[tiab] AND temperature[tiab] ) OR "temperature rise"[tiab] OR "temperature elevat*"[tiab] OR humidity[tiab] OR rainfall[tiab] OR drought[tiab] OR flood*[tiab] OR "circulation model*"[tiab] OR "climate change"[tiab] OR "climate change"[MeSH] OR ( climate[tiab] AND change[tiab] ) OR "future climate"[tiab] OR ( meteorological[tiab] AND ( factor*[tiab] OR variable*[tiab] ) ) OR precipitation[tiab] OR heatwave[tiab] OR "heat wave"[tiab] OR wildfire[tiab] OR storm*[tiab] OR "sea level"[tiab] OR "land cover"[tiab] OR "extreme weather"[tiab] OR temperature[tiab]) AND 2010/01/01:2024/10/06[dp]

**Scopus, malaria** (5257 results)

TITLE-ABS-KEY(malaria* OR black water fever* or blackwater fever* or marsh fever* or falciparum or paludism or plasmodi* or remittent fever* or swamp fever* or vivax or malariae or ovale or knowlesi) AND ( "global warming" OR "temperature increase" OR ( increas* AND temperature ) OR "temperature rise" OR "temperature elevat*" OR humidity OR rainfall OR drought OR flood* OR "circulation model*" OR "climate change" OR ( climate AND chang* ) OR "future climate" OR "greenhouse effect" OR ( meteorological AND ( factor* OR variable* ) ) OR precipitation OR heatwave OR "heat wave" OR wildfireOR storm* OR "sea level" OR "land cover" OR "extreme weather" OR temperature ) AND PUBYEAR > 2009

**WHO-IRIS, NTDs and malaria** (86 results)

dc.subject.mesh:( "Buruli Ulcer" or "ulcerans" or "possum" or "peregrinus" or "vulpecula" or "cunninghami" or "Chagas disease" or "cruzi" or "kissing bug*" or "Triatoma infestans" or "Rhodnius prolixus" or "Triatoma dimidiate" or "Panstrongylus megistus" or "triatom*" or dengue or chikungunya or "Aedes aegypti" or "Aedes albopictus" or "Aedes polynesiensis" or "Aedes scutellaris" or "Aedes furcifer-taylori" or "Aedes africanus" or "Aedes luteocephalus" or "Asian tiger mosquito" or dracunculiasis or "guinea-worm" or "Dracunculus medinensis" or "Cyclops" or "Cystic echinococcosis" or "hydatid" or "Alveolar echinococcosis" or "unicystic echinococcosis" or "polycystic echinococcosis" or "Echinococcus" or "Foodborne trematod*" or "trematodiasis" or "Clonorchis sinensis" or "Fasciola hepatica" or "Opisthorchis viverrini" or "Paragonimus westermani" or "opisthorchiasis" or "fascioliasis" or "paragonimiasis" or "clonorchiasis" or "Human African trypanosom*" or "sleeping sickness" or "gambiense" or "rhodesiense" or "tsetse" or "Leishmani*" or "aethiopica" or "amazonensis" or "braziliensis" or "Leishmania chagasi" or "L. chagasi" or "colombiensis" or "Leishmania donovani" or "L. donovani" or "garnhami" or "guyanensis" or "Leishmania infantum" or "L. infantum" or "killicki" or "lainsoni" or "lindenbergi" or "Leishmania major" or "L. major" or "mexicana" or "naiffi" or "panamensis" or "peruviana" or "pifanoi" or "Leishmania shawi" or "L. shawi" or "Leishmania tropica" or "L. tropica" or "venezuelensis" or "phlebotomine" or "sandfl*" or "sand fl*" or "Lutzomyia" or "Phlebotomus" or "Leprosy" or "Mycobacterium leprae" or "Mycobacterium lepromatosis" or "Lymphatic filariasis" or "Wuchereria bancrofti" or "Brugia malayi" or "Brugia timori" or Culex or Anopheles or "Mansonia" or Aedes or "Mycetoma" or "actinomycetoma" or "eumycetoma" or "chromoblastomycosis" or "paracoccidioidomycosis" or "sporotrichosis" or "deep mycos*" or "Paracoccidioides lutzii" or "Fonsecaea pedrosoi" or "Cladophialophora carrionii" or "Phialophora verrucose" or "Madurella mycetomatis" or "Nocardia brasiliensis" or "Actinomadura madurae" or "Streptomyces somaliensis" or "Actinomadura pelletieri" or "Sporothrix schenckii" or "Onchocerc*" or "river blindness" or blackfl* or "black fl*" or "Simulium" or "Rabies" or "scabies" or "Sarcoptes scabiei" or "jigger flea" or "tungiasis" or "Tunga penetrans" or "chigo*" or "schistosom*" or "bilharzia" or "Bulinus" or "Biomphalaria" or "Oncomelania" or "Robertsiella" or "Neotricula" or "soil transmitted helminth*" or "soil-transmitted helminth*" or "Ascaris lumbricoides" or "roundworm" or "Necator americanus" or "Ancylostoma duodenale" or "hookworm" or "Trichuris trichiura" or "whipworm" or Strongyloid* or "snakebite" or "snake bite" or ( "snake" and "envenom*" ) or "taenia*" or "cysticercosis" or "Taenia solium" "trachoma" or "Chlamydia trachomatis" or "trichiasis" or "Musca sorbens" or "yaws" or "endemic treponematos*" or "pinta" or "bejel" or "nonvenereal endemic syphilis" or "Treponema pallidum pertenue" or "Treponema carateum" or "Treponema pallidum endemicum" or malaria* OR black water fever* or blackwater fever* or marsh fever* or falciparum or paludism or plasmodi* or remittent fever* or swamp fever* or vivax or malariae or ovale or knowlesi or "neglected tropical disease*" or "NTD" or "neglected disease*" or ( ( mosquito-borne or vector-borne ) and disease ) or "neglected disease*")

and dc.subject.mesh:("global warming" or "temperature increase" or (increas* and temperature) or "temperature rise" or "temperature elevat*" or humidity or rainfall or drought or flood* or "circulation model*" or "climate change" or ( climate and chang* ) or "future climate" or "greenhouse effect" or ( meteorological and ( factor* or variable* )) or precipitation or heatwave or "heat wave" or wildfire or storm* or "sea level" or "land cover" or "extreme weather")

**Global Index Medicus, NTDs and malaria** (932 results)

(tw:((Buruli ulcer) OR (ulcerans) OR (possum) OR (peregrinus) OR (vulpecula) OR (cunninghamia) OR (chagas disease) OR (cruzi) OR (kissing bug*) OR (Triatoma infestans) OR (Rhodnius prolixus) OR (Triatoma dimidiate) OR (Panstrongylus megistus) OR (triatom*) OR (Dengue) OR (chikungunya) OR (Aedes aegypti) OR (Aedes albopictus) OR (Aedes polynesiensis) OR (Aedes scutellaris) OR (Aedes furcifer-taylori) OR (Aedes africanus) OR (Aedes luteocephalus) OR (Asian tiger mosquito) OR (Dracunculiasis) OR (guinea-worm) OR (Dracunculus medinensis) OR (Cyclops) OR (Cystic echinococcosis) OR (hydatid) OR (Alveolar echinococcosis) OR (unicystic echinococcosis) OR (polycystic echinococcosis) OR (Echinococcus granulosus) OR (Echinococcus multilocularis) OR (Echinococcus oligarthrus) OR (Echinococcus vogeli) OR (Foodborne trematod*) OR (trematodiases) OR (Clonorchis sinensis) OR (Fasciola hepatica) OR (Opisthorchis viverrini) OR (Paragonimus westermani) OR (opisthorchiasis) OR (fascioliasis) OR (paragonimiasis) OR (clonorchiasis) OR (Human African trypanosom*) OR (sleeping sickness) OR (gambiense) OR (rhodesiense) OR (tsetse) OR (Leishmani*) OR (aethiopica) OR (amazonensis) OR (braziliensis) OR (Leishmania chagasi) OR (L. chagasi) OR (columbiensis) OR (Leishmania donovani) OR (L. donovani) OR (garnham) OR (guyanensis) OR (Leishmania infantum) OR (L. infantum) OR (killickia) OR (lainson) OR (lindenbergia) OR (Leishmania major) OR (L. major) OR (mexicana) OR (naifeh) OR (panamensis) OR (peruviana) OR (pisanoi) OR (Leishmania shawi) OR (L. shawi) OR (Leishmania tropica) OR (L. tropica) OR (venezuelensis) OR (phlebotomine) OR (sandfl*) OR (sand fl*) OR (Lutzomyia) OR (Phlebotomus) OR (Leprosy) OR (Mycobacterium leprae) OR (Mycobacterium lepromatosis) OR (Lymphatic filariasis) OR (Wuchereria bancrofti) OR (Brugia malayi) OR (Brugia timori) OR (Culex) OR (Anopheles) OR (Mansonia) OR (Aedes) OR (Mycetoma) OR (actinomycetoma) OR (eumycetoma) OR (chromoblastomycosis) OR (paracoccidioidomycosis) OR (sporotrichosis) OR (deep mycos*) OR (Paracoccidioides lutzii) OR (Fonsecaea pedrosoi) OR (Cladophialophora carrionii) OR (Phialophora verrucose) OR (Madurella mycetomatis) OR (Nocardia brasiliensis) OR (Actinomadura madurae) OR (Streptomyces somaliensis) OR (Actinomadura pelletieri) OR (Sporothrix schenckii) OR (Onchocerc*) OR (river blindness) OR (blackfl*) OR (black fl*) OR (Simulium) OR (Rabies) OR (scabies) OR (ectoparasite*) OR (Sarcoptes scabiei) OR (jigger flea) OR (tungiasis) OR (Tunga penetrans) OR (chigo*) OR (schistosom*) OR (bilharzia) OR (Bulinus) OR (Biomphalaria) OR (Oncomelania) OR (robertsella) OR (neotricula) OR (soil transmitted helminth*) OR (soil-transmitted helminth*) OR (Ascaris lumbricoides) OR (roundworm) OR (Necator americanus) OR ( Ancylostoma duodenale) OR (hookworm) OR (Trichuris trichiura) OR (whipworm) OR (Strongyloid*) OR (snakebite*) OR (snake bite*) OR (taenia*) OR (cysticercosis) OR (Taenia solium) OR (trachoma) OR (Chlamydia trachomatis) OR (trichiasis) OR (Musca sorbens) OR (yaws) OR (endemic treponematos*) OR (pinta) OR (bejel) OR (nonvenereal endemic syphilis) OR (Treponema pallidum pertenue) OR (Treponema carateum) OR (Treponema pallidum endemicum) OR (neglected tropical disease*) OR (NTDs) OR (mosquito-borne) OR (vector-borne) OR

(malaria*) OR (black water fever*) or (blackwater fever*) or (marsh fever*) or (falciparum) or (paludism) or (plasmodi*) or (remittent fever*) or (swamp fever*) or (vivax) or (malariae) or (ovale) or (knowlesi)) OR (mj:(“Neglected diseases”)))

AND (tw:((global warming) OR (temperature increase) OR (temperature elevat*) OR (temperature rise) OR (humidity) OR (rainfall) OR (drought) OR (flood*) OR (circulation model*) OR (climate change ) OR (future climate ) OR (meteorological factor*) OR (meteorological variable*) OR (precipitation) OR (heatwave) OR (heat wave ) OR (wildfire) OR ( storm*) OR (sea level ) OR ( land cover ) OR (extreme weather)) OR (mj:(“Climate Change”)))

AND (year_cluster:[2010 TO 2023])
