# Supplementary material for: Climate change, malaria and neglected tropical diseases: a scoping review
Source: Trans R Soc Trop Med Hyg. 2024 May 10;118(9):561–79. doi: 10.1093/trstmh/trae026 (PMC11367761; doi:10.1093/trstmh/trae026)
Supplement: trae026_Supplemental_Files [file trae026_supplemental_files.zip › supplementary-table-2.pdf]

Summary table for the Climate Change, NTDs and Malaria scoping review

P Klepac & WHO Task Team

Studies by disease and study type, with references to publications.

Disease	Type of study	References
Buruli ulcer	Field study	[1]
Buruli ulcer	Laboratory research	[2]
Buruli ulcer	Modelling and statistics	[2–6]
Chagas disease	Cohort study	[7]
Chagas disease	Ecological case report	[8]
Chagas disease	Field study	[9]
Chagas disease	Laboratory research	[8,10–12]
Chagas disease	Modelling and statistics	[7–9,12–25]
dengue and chikungunya	Cohort study	[26–28]
dengue and chikungunya	Ecological case report	[29–38]
dengue and chikungunya	Field study	[29,31–33,39–66]
dengue and chikungunya	Laboratory research	[44,45,59,67–84]
dengue and chikungunya	Modelling and statistics	[3,19,26,27,29,31,32,39–43,46–48,50,54–56,61–66,76,77,82,85–153,153–175,175–203]
dengue and chikungunya	Other	[186,204]
echinococcosis	Modelling and statistics	[205–208]
foodborne trematodiases	Ecological case report	[209–212]
foodborne trematodiases	Field study	[209,213,214]
foodborne trematodiases	Laboratory research	[209,215,216]
foodborne trematodiases	Modelling and statistics	[213,217–225]
human African trypanosomiasis	Field study	[226]
human African trypanosomiasis	Laboratory research	[227]
human African trypanosomiasis	Modelling and statistics	[226–233]
leishmaniasis	Ecological case report	[234–242]
leishmaniasis	Field study	[234,236,238,240,241,243–247]
leishmaniasis	Laboratory research	[248,249]
leishmaniasis	Modelling and statistics	[19,25,238,243,247,248,250–276,276–284]
leishmaniasis	Other	[236,246]
leprosy	Modelling and statistics	[3]

Disease	Type of study	References
lymphatic filariasis	Ecological case report	[29,31]
lymphatic filariasis	Field study	[29,31,45,285,286]
lymphatic filariasis	Laboratory research	[45,74,287]
lymphatic filariasis	Modelling and statistics	[29,31,200,288–292]
lymphatic filariasis	Other	[204]
malaria	Cohort study	[26,293–296]
malaria	Ecological case report	[31,297]
malaria	Field study	[31,298–313]
malaria	Laboratory research	[69,80,300,314–326]
malaria	Modelling and statistics	[19,26,31,107,152,153,153,157,172,183,278,294,298–306,308,308–313,320,323,324,326–360,360–433,433–442,442–464]
malaria	Other	[204,307,373]
onchocerciasis	Ecological case report	[465]
onchocerciasis	Field study	[466]
onchocerciasis	Modelling and statistics	[466]
rabies	Ecological case report	[467]
rabies	Modelling and statistics	[172,468–474]
scabies, tungiasis	Cohort study	[26]
scabies, tungiasis	Modelling and statistics	[26]
schistosomiasis	Ecological case report	[475]
schistosomiasis	Field study	[213,476–479]
schistosomiasis	Laboratory research	[480–485]
schistosomiasis	Modelling and statistics	[172,213,221,363,476–479,483,485–499]
snakebite envenoming	Cohort study	[500,501]
snakebite envenoming	Modelling and statistics	[502–510]
snakebite envenoming	Other	[502]
soil-transmitted helminthiases	Field study	[511]
soil-transmitted helminthiases	Laboratory research	[511]

References

1. Morris A, Gozlan RE, Hassani H *et al.* Complex temporal climate signals drive the emergence of human water-borne disease. *Emerging Microbes and Infections* 2014;**3**:e56.

2. Banks SC, Lorin T, Shaw RE *et al.* Fine-scale refuges can buffer demographic and genetic processes against short-term climatic variation and disturbance: A 22-year case study of an arboreal marsupial. *Molecular ecology* 2015;**24**:3831–45.

3. Liu B, Jiao Z, Ma J *et al.* Modelling the potential distribution of arbovirus vector Aedes aegypti under current and future climate scenarios in Taiwan, China. *Pest Manag Sci* 2019;**75**:3076–83.

4. Anagonou GE, Johnson RC, Sopoh GE *et al.* Modeling the distribution of Mycobacterium ulcerans infection in Lalo district, Benin. *Environnement, Risques et Sante* 2020;**19**:20–8.

5. Drancourt M, Zingue D. Variations in temperatures and the incidence of Buruli ulcer in Africa. *Travel Medicine and Infectious Disease* 2020;**36**:101472.

6. Ravensway J van, Benbow ME, Tsonis AA *et al.* Climate and Landscape Factors Associated with Buruli

Ulcer Incidence in Victoria, Australia. *Plos One* 2012;**7**:e51074.
7. Ceccarelli S, Rabinovich JE. Global Climate Change Effects on Venezuela's Vulnerability to Chagas Disease is Linked to the Geographic Distribution of Five Triatomine Species. *Journal of Medical Entomology* 2015;**52**:1333–43.
8. Vianna EN, Souza EGRJP, Souza CR *et al.* Chagas disease ecoepidemiology and environmental changes in northern Minas Gerais state, Brazil. *Memorias Do Instituto Oswaldo Cruz* 2017;**112**:760–8.
9. Ribeiro AC, Sarquis O, Lima MM *et al.* Enduring extreme climate: Effects of severe drought on Triatoma brasiliensis populations in wild and man-made habitats of the Caatinga. *Plos Neglected Tropical Diseases* 2019;**13**:e0007766.
10. Gonzalez-Rete B, Salazar-Schettino PM, Bucio-Torres MI *et al.* Activity of the prophenoloxidase system and survival of triatomines infected with different Trypanosoma cruzi strains under different temperatures: Understanding Chagas disease in the face of climate change. *Parasit Vectors* 2019;**12**:219.
11. Tamayo LD, Guhl F, Vallejo GA *et al.* The effect of temperature increase on the development of Rhodnius prolixus and the course of Trypanosoma cruzi metacyclogenesis. *PLoS Negl Trop Dis* 2018;**12**:e0006735.
12. Clavijo-Baquet S, Cavieres G, González A *et al.* Thermal performance of the Chagas disease vector, Triatoma infestans, under thermal variability. *PLoS Negl Trop Dis* 2021;**15**:e0009148.
13. Carmona-Castro O, Moo-Llanes DA, Ramsey JM. Impact of climate change on vector transmission of Trypanosoma cruzi (Chagas, 1909) in North America. *Med Vet Entomol* 2018;**32**:84–101.
14. Cordovez JM, Rendon LM, Gonzalez C *et al.* Using the basic reproduction number to assess the effects of climate change in the risk of Chagas disease transmission in Colombia. *Acta Trop* 2014;**129**:74–82.
15. Garrido R, Bacigalupo A, Pena-Gomez F *et al.* Potential impact of climate change on the geographical distribution of two wild vectors of Chagas disease in Chile: Mepraia spinolai and Mepraia gajardoi. *Parasit Vectors* 2019;**12**:478.
16. Garza M, Feria Arroyo TP, Casillas EA *et al.* Projected future distributions of vectors of Trypanosoma cruzi in North America under climate change scenarios. *PLoS Negl Trop Dis* 2014;**8**:e2818.
17. Medone P, Ceccarelli S, Parham PE *et al.* The impact of climate change on the geographical distribution of two vectors of Chagas disease: Implications for the force of infection. *Philos Trans R Soc Lond B Biol Sci* 2015;**370**, DOI: 10.1098/rstb.2013.0560.
18. Tapia-Garay V, Figueroa DP, Maldonado A *et al.* Assessing the risk zones of chagas' disease in chile, in a world marked by global climatic change. *Mem Inst Oswaldo Cruz* 2018;**113**:24–9.
19. Escobar LE, Romero-Alvarez D, Leon R *et al.* Declining Prevalence of Disease Vectors Under Climate Change. *Sci Rep* 2016;**6**:39150.
20. González-Salazar C, Meneses-Mosquera AK, Aguirre-Peña A *et al.* Toward New Epidemiological Landscapes of Trypanosoma cruzi (Kinetoplastida, Trypanosomatidae) Transmission under Future Human-Modified Land Cover and Climatic Change in Mexico. *Tropical Medicine and Infectious Disease* 2022;**7**, DOI: 10.3390/tropicalmed7090221.
21. Flores-Lopez CA, Moo-Llanes DA, Romero-Figueroa G *et al.* Potential distributions of the parasite Trypanosoma cruzi and its vector Dipetalogaster maxima highlight areas at risk of Chagas disease transmission in Baja California Sur, Mexico, under climate change. *Medical and Veterinary Entomology* 2022;**36**:469–79.
22. Badel-Mogollon J, Rodriguez-Figueroa L, Parra-Henao G. Spatio-temporal analysis of the biophysical and ecological conditions of Triatoma dimidiata (Hemiptera: Reduviidae: Triatominae) in the northeast region of Colombia. *Biomedica : revista del Instituto Nacional de Salud* 2017;**37**:106–23.
23. Costa J, Dornak LL, Almeida CE *et al.* Distributional potential of the Triatoma brasiliensis species complex at present and under scenarios of future climate conditions. *Parasites and Vectors* 2014;**7**:238.
24. Ayala S, Alvarado S, Cáceres D *et al.* Estimando el efecto del cambio climático sobre el riesgo de la enfermedad de Chagas en Chile por medio del número reproductivo. *Rev méd Chile* 2019;**147**:683–92.
25. Llanes DAM, Baak-Baak CM, Cigarroa-Toledo N *et al.* Influences of climate change on the geographical distribution of three potential reservoirs of Chagas and Leishmaniasis from the Yucatan Peninsula. *NA* 2021;**11**:1–14.
26. Singh N, Mall RK, Banerjee T *et al.* Association between climate and infectious diseases among children in Varanasi city, India: A prospective cohort study. *Science of the Total Environment* 2021;**796**:148769.
27. Cheng J, Bambrick H, Frentiu FD *et al.* Extreme weather events and dengue outbreaks in Guangzhou, China: A time-series quasi-binomial distributed lag non-linear model. *International Journal of Biometeorology* 2021;**65**:1033–42.

28. Bich TH, Quang LN, Ha TT *et al.* Impacts of flood on health: Epidemiologic evidence from Hanoi, Vietnam. *Global health action* 2011;**4**:6356.
29. Dhimal M, Gautam I, Kress A *et al.* Spatio-temporal distribution of dengue and lymphatic filariasis vectors along an altitudinal transect in Central Nepal. *PLoS Negl Trop Dis* 2014;**8**:e3035.
30. Anyamba A, Small JL, Britch SC *et al.* Recent weather extremes and impacts on agricultural production and vector-borne disease outbreak patterns. *Plos One* 2014;**9**:e92538.
31. Dhimal M, Ahrens B, Kuch U. Species composition, seasonal occurrence, habitat preference and altitudinal distribution of malaria and other disease vectors in eastern Nepal. *Parasit Vectors* 2014;**7**:540.
32. Dhimal M, Gautam I, Joshi HD *et al.* Risk factors for the presence of chikungunya and dengue vectors (Aedes aegypti and Aedes albopictus), their altitudinal distribution and climatic determinants of their abundance in central Nepal. *PLoS Negl Trop Dis* 2015;**9**:e0003545.
33. Hall DR, Tokarz RE, Field EN *et al.* Surveillance and genetic data support the introduction and establishment of Aedes albopictus in Iowa, USA. *Sci Rep* 2022;**12**:2143.
34. Aranda C, Martínez MJ, Montalvo T *et al.* Arbovirus surveillance: First dengue virus detection in local aedes albopictus mosquitoes in Europe, Catalonia, Spain, 2015. *Eurosurveillance* 2018;**23**, DOI: 10.2807/1560-7917.ES.2018.23.47.1700837.
35. Lourenço J, Recker M. The 2012 Madeira Dengue Outbreak: Epidemiological Determinants and Future Epidemic Potential. *Plos Neglected Tropical Diseases* 2014;**8**:e3083.
36. Bannister-Tyrrell M, Williams C, Ritchie SA *et al.* Weather-driven variation in dengue activity in Australia examined using a process-based modeling approach. *American Journal of Tropical Medicine and Hygiene* 2013;**88**:65–72.
37. Ruiz-López F, González-Mazo A, Vélez-Mira A *et al.* Presencia de Aedes (Stegomyia) aegypti (Linnaeus, 1762) y su infección natural con el virus del dengue en alturas no registradas para Colombia. *Biomédica (Bogotá)* 2016;**36**:303–8.
38. Gjenero-Margan I, Aleraj B, Krajcar D *et al.* Autochthonous dengue fever in Croatia, August-September 2010. *Euro surveillance : bulletin Europeen sur les maladies transmissibles = European communicable disease bulletin* 2011;**16**:19805–NA.
39. Bouzid M, Colon-Gonzalez FJ, Lung T *et al.* Climate change and the emergence of vector-borne diseases in Europe: Case study of dengue fever. *Bmc Public Health* 2014;**14**:781.
40. Mweya CN, Kimera SI, Stanley G *et al.* Climate Change Influences Potential Distribution of Infected Aedes aegypti Co-Occurrence with Dengue Epidemics Risk Areas in Tanzania. *Plos One* 2016;**11**:e0162649.
41. Bonnin L, Tran A, Herbreteau V *et al.* Predicting the Effects of Climate Change on Dengue Vector Densities in Southeast Asia through Process-Based Modeling. *Environ Health Perspect* 2022;**130**:127002.
42. Cunze S, Kochmann J, Koch LK *et al.* Aedes albopictus and Its Environmental Limits in Europe. *Plos One* 2016;**11**:e0162116.
43. Del Lesto I, De Liberato C, Casini R *et al.* Is Asian tiger mosquito (Aedes albopictus) going to become homodynamic in Southern Europe in the next decades due to climate change? *R Soc Open Sci* 2022;**9**:220967.
44. Edillo F, Ymbong RR, Bolneo AA *et al.* Temperature, season, and latitude influence development-related phenotypes of Philippine Aedes aegypti (Linnaeus): Implications for dengue control amidst global warming. *Parasit Vectors* 2022;**15**:74.
45. Edillo F, Ymbong RR, Cabahug MM *et al.* Yearly variations of the genetic structure of Aedes aegypti (Linnaeus) (Diptera: Culicidae) in the Philippines (2017-2019). *Infect Genet Evol* 2022;**102**:105296.
46. Equihua M, Ibáñez-Bernal S, Benítez G *et al.* Establishment of Aedes aegypti (L.) in mountainous regions in Mexico: Increasing number of population at risk of mosquito-borne disease and future climate conditions. *Acta Trop* 2017;**166**:316–27.
47. Ivanescu LM, Bodale I, Grigore-Hristodorescu S *et al.* The Risk of Emerging of Dengue Fever in Romania, in the Context of Global Warming. *Trop Med Infect Dis* 2023;**8**, DOI: 10.3390/tropicalmed8010065.
48. Kraemer MUG, Reiner RC, Brady OJ *et al.* Past and future spread of the arbovirus vectors Aedes aegypti and Aedes albopictus. *Nat Microbiol* 2019;**4**:854–63.
49. Kumar G, Pande V, Pasi S *et al.* Air versus water temperature of aquatic habitats in Delhi: Implications for transmission dynamics of Aedes aegypti. *Geospat Health* 2018;**13**, DOI: 10.4081/gh.2018.707.
50. Lamy K, Tran A, Portafaix T *et al.* Impact of regional climate change on the mosquito vector Aedes albopictus in a tropical island environment: La Réunion. *Sci Total Environ* 2023;**875**:162484.
51. Mohammed A, Chadee DD. Effects of different temperature regimens on the development of Aedes

aegypti (L.) (Diptera: Culicidae) mosquitoes. *Acta Trop* 2011;**119**:38–43.
52. Montini P, De Majo MS, Fischer S. Delayed mortality effects of cold fronts during the winter season on Aedes aegypti in a temperate region. *J Therm Biol* 2021;**95**:102808.
53. Padmanabha H, Bolker B, Lord CC *et al.* Food availability alters the effects of larval temperature on Aedes aegypti growth. *J Med Entomol* 2011;**48**:974–84.
54. Pedrosa MC, Borges MAZ, Eiras Á E *et al.* Invasion of Tropical Montane Cities by Aedes aegypti and Aedes albopictus (Diptera: Culicidae) Depends on Continuous Warm Winters and Suitable Urban Biotopes. *J Med Entomol* 2021;**58**:333–42.
55. Rochlin I, Ninivaggi DV, Hutchinson ML *et al.* Climate change and range expansion of the Asian tiger mosquito (Aedes albopictus) in Northeastern USA: Implications for public health practitioners. *Plos One* 2013;**8**:e60874.
56. Roiz D, Neteler M, Castellani C *et al.* Climatic factors driving invasion of the tiger mosquito (Aedes albopictus) into new areas of Trentino, northern Italy. *Plos One* 2011;**6**:e14800.
57. Salinas WS, Feria-Arroyo TP, Vitek CJ. Temperatures Influence Susceptibility to Insecticides in Aedes aegypti and Aedes albopictus (Diptera: Culicidae) Mosquitoes. *Pathogens* 2021;**10**, DOI: 10.3390/pathogens10080992.
58. Sayono S, Nurullita U, Sumanto D *et al.* Altitudinal distribution of Aedes indices during dry season in the dengue endemic area of Central Java, Indonesia. *Ann Parasitol* 2017;**63**:213–21.
59. Urbanski J, Mogi M, O'Donnell D *et al.* Rapid adaptive evolution of photoperiodic response during invasion and range expansion across a climatic gradient. *Am Nat* 2012;**179**:490–500.
60. Xuan le TT, Van Hau P, Thu do T *et al.* Estimates of meteorological variability in association with dengue cases in a coastal city in northern Vietnam: An ecological study. *Glob Health Action* 2014;**7**:23119.
61. Cheng J, Bambrick H, Yakob L *et al.* Extreme weather conditions and dengue outbreak in Guangdong, China: Spatial heterogeneity based on climate variability. *Environmental Research* 2021;**196**:110900.
62. Udayanga L, Gunathilaka N, Iqbal MCM *et al.* Climate change induced vulnerability and adaption for dengue incidence in Colombo and Kandy districts: The detailed investigation in Sri Lanka. *Infectious Diseases of Poverty* 2020;**9**:102.
63. Diaz-Castro S, Moreno-Legorreta M, Ortega-Rubio A *et al.* Relation between dengue and climate trends in the Northwest of Mexico. *Tropical Biomedicine* 2017;**34**:157–65.
64. Mendez-Lazaro P, Muller-Karger FE, Otis D *et al.* Assessing climate variability effects on dengue incidence in San Juan, Puerto Rico. *International Journal of Environmental Research and Public Health* 2014;**11**:9409–28.
65. Petrić M, Lalic B, Pajovic I *et al.* Expected Changes of Montenegrin Climate, Impact on the Establishment and Spread of the Asian Tiger Mosquito (Aedes albopictus), and Validation of the Model and Model-Based Field Sampling. *Atmosphere* 2018;**9**:453–NA.
66. Lippi CA, Stewart-Ibarra AM, Loor MEFB *et al.* Geographic shifts in Aedes aegypti habitat suitability in Ecuador using larval surveillance data and ecological niche modeling: Implications of climate change for public health vector control. *NA* 2018;**NA**:404293–NA.
67. Alam MS, Tuno N. Reduction of Reproductive Capacity in Aedes albopictus (Diptera: Culicidae) in Hot, Dry Summer. *J Med Entomol* 2019;**56**:1729–33.
68. Bellone R, Lechat P, Mousson L *et al.* Climate change and vector-borne diseases: A multi-omics approach of temperature-induced changes in the mosquito. *J Travel Med* 2023;**30**, DOI: 10.1093/jtm/taad062.
69. Buxton M, Nyamukondiwa C, Dalu T *et al.* Implications of increasing temperature stress for predatory biocontrol of vector mosquitoes. *Parasit Vectors* 2020;**13**:604.
70. Christofferson RC, Mores CN. Potential for Extrinsic Incubation Temperature to Alter Interplay Between Transmission Potential and Mortality of Dengue-Infected Aedes aegypti. *Environ Health Insights* 2016;**10**:119–23.
71. Nascimento Neto JF do, Mota AJ da, Roque RA *et al.* Analysis of the transcription of genes encoding heat shock proteins (hsp) in Aedes aegypti Linnaeus, 1762 (Diptera: Culicidae), maintained under climatic conditions provided by the IPCC (Intergovernmental Panel On Climate Change) for the year 2100. *Infect Genet Evol* 2020;**86**:104626.
72. Ezeakacha NF, Yee DA. The role of temperature in affecting carry-over effects and larval competition in the globally invasive mosquito Aedes albopictus. *Parasit Vectors* 2019;**12**:123.
73. Kramer IM, Pfeiffer M, Steffens O *et al.* The ecophysiological plasticity of Aedes aegypti and Aedes

albopictus concerning overwintering in cooler ecoregions is driven by local climate and acclimation capacity. *Sci Total Environ* 2021;**778**:146128.
74. Leonel BF, Koroiva R, Hamada N *et al.* Potential Effects of Climate Change on Ecological Interaction Outcomes Between Two Disease-Vector Mosquitoes: A Mesocosm Experimental Study. *J Med Entomol* 2015;**52**:866–72.
75. Padmanabha H, Lord CC, Lounibos LP. Temperature induces trade-offs between development and starvation resistance in Aedes aegypti (L.) larvae. *Med Vet Entomol* 2011;**25**:445–53.
76. Pech-May A, Moo-Llanes DA, Puerto-Avila MB *et al.* Population genetics and ecological niche of invasive Aedes albopictus in Mexico. *Acta Trop* 2016;**157**:30–41.
77. Piovezan-Borges AC, Valente-Neto F, Tadei WP *et al.* Simulated climate change, but not predation risk, accelerates Aedes aegypti emergence in a microcosm experiment in western Amazonia. *Plos One* 2020;**15**:e0241070.
78. Sukiato F, Wasserman RJ, Foo SC *et al.* The effects of temperature and shading on mortality and development rates of Aedes aegypti (Diptera: Culicidae). *J Vector Ecol* 2019;**44**:264–70.
79. Tuno N, Phong TV, Takagi M. Climate Change May Restrict the Predation Efficiency of Mesocyclops aspericornis (Copepoda: Cyclopidae) on Aedes aegypti (Diptera: Culicidae) Larvae. *Insects* 2020;**11**, DOI: 10.3390/insects11050307.
80. Singh P, Pande V, Dhiman RC. Revisiting the Impact of Temperature on Survival of Anopheles stephensi and Aedes aegypti and Implications on Extrinsic Incubation Period. *Journal of Communicable Diseases* 2022;**54**:60–6.
81. Phanitchat T, Apiwathnasorn C, Sumroiphon S *et al.* The influence of temperature on the developmental rate and survival of Aedes albopictus in Thailand. *Southeast Asian Journal of Tropical Medicine and Public Health* 2017;**48**:799–808.
82. Thomas SM, Fischer D, Fleischmann S *et al.* Risk assessment of dengue virus amplification in Europe based on spatio-temporal high resolution climate change projections. *Erdkunde* 2011;**65**:137–50.
83. Smith C, Baldwin AH, Sullivan J *et al.* Effects of elevated atmospheric CO2 on competition between the mosquitoes aedes albopictus and ae. Triseriatus via changes in litter quality and production. *Journal of Medical Entomology* 2013;**50**:521–32.
84. Bader CA, Williams CR. Mating, ovariole number and sperm production of the dengue vector mosquito Aedes aegypti (L.) in Australia: Broad thermal optima provide the capacity for survival in a changing climate. *Physiological Entomology* 2012;**37**:136–44.
85. Banu S, Hu W, Guo Y *et al.* Projecting the impact of climate change on dengue transmission in Dhaka, Bangladesh. *Environ Int* 2014;**63**:137–42.
86. Butterworth MK, Morin CW, Comrie AC. An Analysis of the Potential Impact of Climate Change on Dengue Transmission in the Southeastern United States. *Environ Health Perspect* 2017;**125**:579–85.
87. Colon-Gonzalez FJ, Fezzi C, Lake IR *et al.* The effects of weather and climate change on dengue. *PLoS Negl Trop Dis* 2013;**7**:e2503.
88. Cunze S, Koch LK, Kochmann J *et al.* Aedes albopictus and Aedes japonicus - two invasive mosquito species with different temperature niches in Europe. *Parasites & Vectors* 2016;**9**:573.
89. Fischer D, Thomas SM, Suk JE *et al.* Climate change effects on Chikungunya transmission in Europe: Geospatial analysis of vector's climatic suitability and virus' temperature requirements. *Int J Health Geogr* 2013;**12**:51.
90. Jia P, Chen X, Chen J *et al.* How does the dengue vector mosquito Aedes albopictus respond to global warming? *Parasites & Vectors* 2017;**10**:140.
91. Lee H, Kim JE, Lee S *et al.* Potential effects of climate change on dengue transmission dynamics in Korea. *Plos One* 2018;**13**:e0199205.
92. Liu-Helmersson J, Brannstrom A, Sewe MO *et al.* Estimating Past, Present, and Future Trends in the Global Distribution and Abundance of the Arbovirus Vector Aedes aegypti Under Climate Change Scenarios. *Front Public Health* 2019;**7**:148.
93. Liu-Helmersson J, Stenlund H, Wilder-Smith A *et al.* Vectorial capacity of Aedes aegypti: Effects of temperature and implications for global dengue epidemic potential. *Plos One* 2014;**9**:e89783.
94. Messina JP, Brady OJ, Golding N *et al.* The current and future global distribution and population at risk of dengue. *Nature Microbiology* 2019;**4**:1508–15.
95. Metelmann S, Caminade C, Jones AE *et al.* The UK's suitability for Aedes albopictus in current and

future climates. *J R Soc Interface* 2019;**16**:20180761.
96. Ogden NH, Milka R, Caminade C *et al.* Recent and projected future climatic suitability of North America for the Asian tiger mosquito Aedes albopictus. *Parasit Vectors* 2014;**7**:532.
97. Proestos Y, Christophides GK, Erguler K *et al.* Present and future projections of habitat suitability of the Asian tiger mosquito, a vector of viral pathogens, from global climate simulation. *Philos Trans R Soc Lond B Biol Sci* 2015;**370**:20130554.
98. Robert MA, Christofferson RC, Weber PD *et al.* Temperature impacts on dengue emergence in the United States: Investigating the role of seasonality and climate change. *Epidemics* 2019;**28**:100344.
99. Tjaden NB, Suk JE, Fischer D *et al.* Modelling the effects of global climate change on Chikungunya transmission in the 21 st century. *Sci Rep* 2017;**7**:3813.
100. Williams CR, Mincham G, Ritchie SA *et al.* Bionomic response of Aedes aegypti to two future climate change scenarios in far north Queensland, Australia: Implications for dengue outbreaks. *Parasit Vectors* 2014;**7**:447.
101. Abdalgader T, Pedersen M, Ren D *et al.* Trade-off between climatic and human population impacts on Aedes aegypti life history shapes its geographic distribution. *J Theor Biol* 2022;**535**:110987.
102. Akter R, Hu W, Gatton M *et al.* Climate variability, socio-ecological factors and dengue transmission in tropical Queensland, Australia: A Bayesian spatial analysis. *Environ Res* 2021;**195**:110285.
103. Aström C, Rocklöv J, Hales S *et al.* Potential distribution of dengue fever under scenarios of climate change and economic development. *Ecohealth* 2012;**9**:448–54.
104. Campbell LP, Luther C, Moo-Llanes D *et al.* Climate change influences on global distributions of dengue and chikungunya virus vectors. *Philos Trans R Soc Lond B Biol Sci* 2015;**370**, DOI: 10.1098/rstb.2014.0135.
105. Capinha C, Rocha J, Sousa CA. Macroclimate determines the global range limit of Aedes aegypti. *Ecohealth* 2014;**11**:420–8.
106. Chen MJ, Lin CY, Wu YT *et al.* Effects of extreme precipitation to the distribution of infectious diseases in Taiwan, 1994-2008. *Plos One* 2012;**7**:e34651.
107. Colón-González FJ, Sewe MO, Tompkins AM *et al.* Projecting the risk of mosquito-borne diseases in a warmer and more populated world: A multi-model, multi-scenario intercomparison modelling study. *Lancet Planet Health* 2021;**5**:e404–14.
108. Davis C, Murphy AK, Bambrick H *et al.* A regional suitable conditions index to forecast the impact of climate change on dengue vectorial capacity. *Environ Res* 2021;**195**:110849.
109. Descloux E, Mangeas M, Menkes CE *et al.* Climate-based models for understanding and forecasting dengue epidemics. *PLoS Negl Trop Dis* 2012;**6**:e1470.
110. Dostal T, Meisner J, Munayco C *et al.* The effect of weather and climate on dengue outbreak risk in Peru, 2000-2018: A time-series analysis. *PLoS Negl Trop Dis* 2022;**16**:e0010479.
111. Echeverry-Cárdenas E, López-Castañeda C, Carvajal-Castro JD *et al.* Potential geographic distribution of the tiger mosquito Aedes albopictus (Skuse, 1894) (Diptera: Culicidae) in current and future conditions for Colombia. *PLoS Negl Trop Dis* 2021;**15**:e0008212.
112. Fukui S, Kuwano Y, Ueno K *et al.* Modeling the effect of rainfall changes to predict population dynamics of the Asian tiger mosquito Aedes albopictus under future climate conditions. *Plos One* 2022;**17**:e0268211.
113. Georgiades P, Proestos Y, Lelieveld J *et al.* Machine Learning Modeling of Aedes albopictus Habitat Suitability in the 21st Century. *Insects* 2023;**14**, DOI: 10.3390/insects14050447.
114. Hayashi K, Fujimoto M, Nishiura H. Quantifying the future risk of dengue under climate change in Japan. *Front Public Health* 2022;**10**:959312.
115. Hussain SSA, Dhiman RC. Distribution Expansion of Dengue Vectors and Climate Change in India. *Geohealth* 2022;**6**:e2021GH000477.
116. Iwamura T, Guzman-Holst A, Murray KA. Accelerating invasion potential of disease vector Aedes aegypti under climate change. *Nat Commun* 2020;**11**:2130.
117. Kakarla SG, Bhimala KR, Kadiri MR *et al.* Dengue situation in India: Suitability and transmission potential model for present and projected climate change scenarios. *Sci Total Environ* 2020;**739**:140336.
118. Kamal M, Kenawy MA, Rady MH *et al.* Mapping the global potential distributions of two arboviral vectors Aedes aegypti and Ae. Albopictus under changing climate. *Plos One* 2018;**13**:e0210122.
119. Kamiya T, Greischar MA, Wadhawan K *et al.* Temperature-dependent variation in the extrinsic incubation period elevates the risk of vector-borne disease emergence. *Epidemics* 2019;**30**:100382.
120. Khan SU, Ogden NH, Fazil AA *et al.* Current and Projected Distributions of Aedes aegypti and Ae.

Albopictus in Canada and the U.S. *Environ Health Perspect* 2020;**128**:57007.
121. Koch LK, Cunze S, Werblow A *et al.* Modeling the habitat suitability for the arbovirus vector Aedes albopictus (Diptera: Culicidae) in Germany. *Parasitol Res* 2016;**115**:957–64.
122. Laporta GZ, Potter AM, Oliveira JFA *et al.* Global Distribution of Aedes aegypti and Aedes albopictus in a Climate Change Scenario of Regional Rivalry. *Insects* 2023;**14**, DOI: 10.3390/insects14010049.
123. Li C, Wang X, Wu X *et al.* Modeling and projection of dengue fever cases in Guangzhou based on variation of weather factors. *Sci Total Environ* 2017;**605-606**:867–73.
124. Li R, Xu L, Bjørnstad ON *et al.* Climate-driven variation in mosquito density predicts the spatiotemporal dynamics of dengue. *Proc Natl Acad Sci U S A* 2019;**116**:3624–9.
125. Liu B, Gao X, Ma J *et al.* Modeling the present and future distribution of arbovirus vectors Aedes aegypti and Aedes albopictus under climate change scenarios in Mainland China. *Sci Total Environ* 2019;**664**:203–14.
126. Liu H, Huang X, Guo X *et al.* Climate change and Aedes albopictus risks in China: Current impact and future projection. *Infect Dis Poverty* 2023;**12**:26.
127. Liu Q, Zhang HD, Xing D *et al.* The predicted potential distribution of Aedes albopictus in China under the shared socioeconomic pathway (SSP)1-2.6. *Acta Trop* 2023;**248**:107001.
128. Liu-Helmersson J, Quam M, Wilder-Smith A *et al.* Climate Change and Aedes Vectors: 21st Century Projections for Dengue Transmission in Europe. *EBioMedicine* 2016;**7**:267–77.
129. Liu-Helmersson J, Rocklöv J, Sewe M *et al.* Climate change may enable Aedes aegypti infestation in major European cities by 2100. *Environ Res* 2019;**172**:693–9.
130. Mogi M, Tuno N. Impact of climate change on the distribution of Aedes albopictus (Diptera: Culicidae) in northern Japan: Retrospective analyses. *J Med Entomol* 2014;**51**:572–9.
131. Ng V, Fazil A, Gachon P *et al.* Assessment of the Probability of Autochthonous Transmission of Chikungunya Virus in Canada under Recent and Projected Climate Change. *Environ Health Perspect* 2017;**125**:067001.
132. Obenauer JF, Andrew Joyner T, Harris JB. The importance of human population characteristics in modeling Aedes aegypti distributions and assessing risk of mosquito-borne infectious diseases. *Trop Med Health* 2017;**45**:38.
133. Oliveira S, Rocha J, Sousa CA *et al.* Wide and increasing suitability for Aedes albopictus in Europe is congruent across distribution models. *Sci Rep* 2021;**11**:9916.
134. Ordoñez-Sierra R, Mastachi-Loza CA, Díaz-Delgado C *et al.* Spatial Risk Distribution of Dengue Based on the Ecological Niche Model of Aedes aegypti (Diptera: Culicidae) in the Central Mexican Highlands. *J Med Entomol* 2020;**57**:728–37.
135. Portilla Cabrera CV, Selvaraj JJ. Geographic shifts in the bioclimatic suitability for Aedes aegypti under climate change scenarios in Colombia. *Heliyon* 2020;**6**:e03101.
136. Santos JM, Capinha C, Rocha J *et al.* The current and future distribution of the yellow fever mosquito (Aedes aegypti) on Madeira Island. *PLoS Negl Trop Dis* 2022;**16**:e0010715.
137. Shabani F, Shafapour Tehrany M, Solhjouy-Fard S *et al.* A comparative modeling study on non-climatic and climatic risk assessment on Asian Tiger Mosquito (Aedes albopictus). *PeerJ* 2018;**6**:e4474.
138. Shabbir W, Pilz J, Naeem A. A spatial-temporal study for the spread of dengue depending on climate factors in Pakistan (2006-2017). *Bmc Public Health* 2020;**20**:995.
139. Singh PS, Chaturvedi HK. A retrospective study of environmental predictors of dengue in Delhi from 2015 to 2018 using the generalized linear model. *Sci Rep* 2022;**12**:8109.
140. Stratton MD, Ehrlich HY, Mor SM *et al.* A comparative analysis of three vector-borne diseases across Australia using seasonal and meteorological models. *Sci Rep* 2017;**7**:40186.
141. Struchiner CJ, Rocklöv J, Wilder-Smith A *et al.* Increasing Dengue Incidence in Singapore over the Past 40 Years: Population Growth, Climate and Mobility. *Plos One* 2015;**10**:e0136286.
142. Suresh S, Meraj G, Kumar P *et al.* Interactions of urbanisation, climate variability, and infectious disease dynamics: Insights from the Coimbatore district of Tamil Nadu. *Environ Monit Assess* 2023;**195**:1226.
143. Thomas SM, Tjaden NB, Frank C *et al.* Areas with High Hazard Potential for Autochthonous Transmission of Aedes albopictus-Associated Arboviruses in Germany. *Int J Environ Res Public Health* 2018;**15**, DOI: 10.3390/ijerph15061270.
144. Thomas SM, Tjaden NB, Bos S van den *et al.* Implementing cargo movement into climate based risk assessment of vector-borne diseases. *Int J Environ Res Public Health* 2014;**11**:3360–74.

145. Trájer AJ. Aedes aegypti in the Mediterranean container ports at the time of climate change: A time bomb on the mosquito vector map of Europe. *Heliyon* 2021;**7**:e07981.
146. Tran BL, Tseng WC, Chen CC *et al.* Estimating the Threshold Effects of Climate on Dengue: A Case Study of Taiwan. *Int J Environ Res Public Health* 2020;**17**, DOI: 10.3390/ijerph17041392.
147. Vincenti-Gonzalez MF, Tami A, Lizarazo EF *et al.* ENSO-driven climate variability promotes periodic major outbreaks of dengue in Venezuela. *Sci Rep* 2018;**8**:5727.
148. Wiese D, Escalante AA, Murphy H *et al.* Integrating environmental and neighborhood factors in MaxEnt modeling to predict species distributions: A case study of Aedes albopictus in southeastern Pennsylvania. *Plos One* 2019;**14**:e0223821.
149. Williams CR, Mincham G, Faddy H *et al.* Projections of increased and decreased dengue incidence under climate change. *Epidemiol Infect* 2016;**144**:3091–100.
150. Saeed A, Ali S, Khan F *et al.* Modelling the impact of climate change on dengue outbreaks and future spatiotemporal shift in Pakistan. *Environmental Geochemistry and Health* 2023;**45**:3489–505.
151. Wint W, Jones P, Kraemer M *et al.* Past, present and future distribution of the yellow fever mosquito Aedes aegypti: The European paradox. *Science of the Total Environment* 2022;**847**:157566.
152. Sargent K, Mollard J, Henley SF *et al.* Predicting Transmission Suitability of Mosquito-Borne Diseases under Climate Change to Underpin Decision Making. *International Journal of Environmental Research and Public Health* 2022;**19**, DOI: 10.3390/ijerph192013656.
153. Mondal J, Das A, Khatun R. Predicting climate change and its impact on future occurrences of vector-borne diseases in West Bengal, India. *Environment, Development and Sustainability* 2022;**24**:11871–94.
154. Zhao J, He G, Xiao J *et al.* Mechanism of temperature on dengue fever transmission and impact of future temperature change on its transmission risk. *Journal of Environmental and Occupational Medicine* 2022;**39**:309–14.
155. Paul KK, Macadam I, Green D *et al.* Dengue transmission risk in a changing climate: Bangladesh is likely to experience a longer dengue fever season in the future. *Environmental Research Letters* 2021;**16**, DOI: 10.1088/1748-9326/ac2b60.
156. Tjaden NB, Cheng Y, Beierkuhnlein C *et al.* Chikungunya beyond the tropics: Where and when do we expect disease transmission in Europe? *Viruses* 2021;**13**, DOI: 10.3390/v13061024.
157. Karuppusamy B, Sarma DK, Lalmalsawma P *et al.* Effect of climate change and deforestation on vector borne diseases in the North-Eastern Indian State of Mizoram bordering Myanmar. *Journal of Climate Change and Health* 2021;**2**, DOI: 10.1016/j.joclim.2021.100015.
158. Bal S, Sodoudi S. Modeling and prediction of dengue occurrences in Kolkata, India, based on climate factors. *International Journal of Biometeorology* 2020;**64**:1379–91.
159. Rohat G, Monaghan A, Hayden MH *et al.* Intersecting vulnerabilities: Climatic and demographic contributions to future population exposure to Aedes-borne viruses in the United States. *Environmental Research Letters* 2020;**15**, DOI: 10.1088/1748-9326/ab9141.
160. Sintayehu DW, Tassie N, De Boer WF. Present and future climatic suitability for dengue fever in Africa. *Infection Ecology and Epidemiology* 2020;**10**:1782042.
161. Oidtman RJ, Lai S, Huang Z *et al.* Inter-annual variation in seasonal dengue epidemics driven by multiple interacting factors in Guangzhou, China. *Nature Communications* 2019;**10**:1148.
162. Jácome G, Vilela P, Yoo C. Present and future incidence of dengue fever in Ecuador nationwide and coast region scale using species distribution modeling for climate variability's effect. *Ecological Modelling* 2019;**400**:60–72.
163. Chandran R, Azeez PA. Outbreak of dengue in Tamil Nadu, India. *Current Science* 2015;**109**:171–6.
164. Trájer A, Bede-Fazekas Á, Bobvos J *et al.* Seasonality and geographical occurrence of West Nile fever and distribution of Asian tiger mosquito. *Idojaras* 2014;**118**:19–40.
165. Chaves LF, Morrison AC, Kitron UD *et al.* Nonlinear impacts of climatic variability on the density-dependent regulation of an insect vector of disease. *Global Change Biology* 2012;**18**:457–68.
166. Erickson RA, Hayhoe K, Presley SM *et al.* Potential impacts of climate change on the ecology of dengue and its mosquito vector the Asian tiger mosquito (Aedes albopictus). *Environmental Research Letters* 2012;**7**, DOI: 10.1088/1748-9326/7/3/034003.
167. Fischer D, Thomas SM, Niemitz F *et al.* Projection of climatic suitability for Aedes albopictus Skuse (Culicidae) in Europe under climate change conditions. *Global and Planetary Change* 2011;**78**:54–64.
168. Colon-Gonzalez FJ, Gibb R, Khan K *et al.* Projecting the future incidence and burden of dengue in

Southeast Asia. *Nature Communications* 2023;**14**:5439.
169. Li C, Liu Z, Li W *et al.* Projecting future risk of dengue related to hydrometeorological conditions in mainland China under climate change scenarios: A modelling study. *The Lancet Planetary Health* 2023;**7**:e397–406.
170. Lopez MS, Gomez AA, Muller GV *et al.* Relationship between Climate Variables and Dengue Incidence in Argentina. *Environmental Health Perspectives* 2023;**131**:057008.
171. Wang Y, Zhao S, Wei Y *et al.* Impact of climate change on dengue fever epidemics in South and Southeast Asian settings: A modelling study. *Infectious Disease Modelling* 2023;**8**:645–55.
172. Cao B, Bai C, Wu K *et al.* Tracing the future of epidemics: Coincident niche distribution of host animals and disease incidence revealed climate-correlated risk shifts of main zoonotic diseases in China. *Global Change Biology* 2023;**29**:3723–46.
173. Islam MA, Hasan MN, Tiwari A *et al.* Correlation of Dengue and Meteorological Factors in Bangladesh: A Public Health Concern. *International Journal of Environmental Research and Public Health* 2023;**20**:5152.
174. Sedaghat M, Omid F, Karimi M *et al.* Modelling the probability of presence of Aedes aegypti and Aedes albopictus in Iran until 2070. *Asian Pacific Journal of Tropical Medicine* 2023;**16**:16–25.
175. Sarma DK, Kumar M, Nina PB *et al.* An assessment of remotely sensed environmental variables on Dengue epidemiology in Central India. *Plos Neglected Tropical Diseases* 2022;**16**:e0010859.
176. Ochida N, Mangeas M, Dupont-Rouzeyrol M *et al.* Modeling present and future climate risk of dengue outbreak, a case study in New Caledonia. *Environmental Health: A Global Access Science Source* 2022;**21**:20.
177. Wu W, Ren H, Lu L. Increasingly expanded future risk of dengue fever in the Pearl River Delta, China. *Plos Neglected Tropical Diseases* 2021;**15**:e0009745.
178. Lowe R, Lee SA, O'Reilly KM *et al.* Combined effects of hydrometeorological hazards and urbanisation on dengue risk in Brazil: A spatiotemporal modelling study. *The Lancet Planetary Health* 2021;**5**:e209–19.
179. Harapan H, Yufika A, Anwar S *et al.* Effects of El Nino Southern Oscillation and Dipole mode index on Chikungunya infection in Indonesia. *Tropical Medicine and Infectious Disease* 2020;**5**:119.
180. Cheng J, Bambrick H, Yakob L *et al.* Heatwaves and dengue outbreaks in Hanoi, Vietnam: New evidence on early warning. *Plos Neglected Tropical Diseases* 2020;**14**:1–15.
181. Henry S, Mendonca FA. Past, present, and future vulnerability to dengue in jamaica: A spatial analysis of monthly variations. *International Journal of Environmental Research and Public Health* 2020;**17**:3156.
182. Tuladhar R, Singh A, Varma A *et al.* Climatic factors influencing dengue incidence in an epidemic area of Nepal. *BMC research notes* 2019;**12**:131.
183. Tang L, Furushima Y, Honda Y *et al.* Estimating human health damage factors related to CO2 emissions by considering updated climate-related relative risks. *International Journal of Life Cycle Assessment* 2019;**24**:1118–28.
184. Acharya BK, Cao C, Xu M *et al.* Present and future of dengue fever in nepal: Mapping climatic suitability by ecological niche model. *International Journal of Environmental Research and Public Health* 2018;**15**:187.
185. Colon-Gonzalez FJ, Harris I, Osborn TJ *et al.* Limiting global-mean temperature increase to 1.5-2 degreeC could reduce the incidence and spatial spread of dengue fever in Latin America. *Proceedings of the National Academy of Sciences of the United States of America* 2018;**115**:6243–8.
186. Wu X, Lang L, Ma W *et al.* Non-linear effects of mean temperature and relative humidity on dengue incidence in Guangzhou, China. *Science of the Total Environment* 2018;**628-629**:766–71.
187. Xiang J, Hansen A, Liu Q *et al.* Association between dengue fever incidence and meteorological factors in Guangzhou, China, 2005-2014. *Environmental Research* 2017;**153**:17–26.
188. Siraj AS, Oidtman RJ, Huber JH *et al.* Temperature modulates dengue virus epidemic growth rates through its effects on reproduction numbers and generation intervals. *Plos Neglected Tropical Diseases* 2017;**11**:e0005797.
189. Teurlai M, Menkes CE, Cavarero V *et al.* Socio-economic and Climate Factors Associated with Dengue Fever Spatial Heterogeneity: A Worked Example in New Caledonia. *Plos Neglected Tropical Diseases* 2015;**9**:e0004211.
190. Williams CR, Gill BS, Mincham G *et al.* Testing the impact of virus importation rates and future climate change on dengue activity in Malaysia using a mechanistic entomology and disease model. *Epidemiology and Infection* 2015;**143**:2856–64.
191. Neteler M, Metz M, Rocchini D *et al.* Is Switzerland suitable for the invasion of Aedes albopictus? *Plos*

One 2013;**8**:e82090.
192. Huang X, Williams G, Clements ACA *et al.* Imported dengue cases, weather variation and autochthonous dengue incidence in Cairns, Australia. *Plos One* 2013;**8**:e81887.
193. Hu W, Clements A, Tong S *et al.* Spatial patterns and socioecological drivers of dengue fever transmission in queensland, Australia. *Environmental Health Perspectives* 2012;**120**:260–6.
194. Carbajo AE, Cardo MV, Vezzani D. Is temperature the main cause of dengue rise in non-endemic countries? The case of Argentina. *International Journal of Health Geographics* 2012;**11**:26.
195. Maria-Ruth BP-C. Modeling and predicting dengue fever cases in key regions of the Philippines using remote sensing data. *Asian Pacific Journal of Tropical Medicine* 2019;**12**:60–6.
196. Khormi HM, Kumar L. Climate change and the potential global distribution of Aedes aegypti: Spatial modelling using geographical information system and CLIMEX. *Geospatial Health* 2014;**8**:405–15.
197. Monaghan AJ, Sampson K, Steinhoff DF *et al.* The potential impacts of 21st century climatic and population changes on human exposure to the virus vector mosquito Aedes aegypti. *Climatic Change* 2016;**146**:487–500.
198. Fan J-C, Liu Q-Y. Potential impacts of climate change on dengue fever distribution using RCP scenarios in China. *Advances in Climate Change Research* 2019;**10**:1–8.
199. Ryan SJ, Carlson CJ, Mordecai EA *et al.* Global expansion and redistribution of Aedes-borne virus transmission risk with climate change. *Plos Neglected Tropical Diseases* 2019;**13**:e0007213–NA.
200. Ragab SH, Tyshenko MG. Predicting the potential worldwide distribution of Aedes aegypti under climate change scenarios. *International Journal of Scientific Reports* 2023;**9**:344–52.
201. Franchito SH, Rao VB, Fernandez JPR *et al.* Future Changes in Climatic Variables Due to Greenhouse Warming Increases Dengue Incidence in the Region of the Tucurui Hydroelectric Dam in the Amazon. *Pure and Applied Geophysics* 2021;**178**:1–15.
202. Khan M, Pedersen M, Zhu M *et al.* Dengue transmission under future climate and human population changes in mainland China. *Applied Mathematical Modelling* 2023;**114**:785–98.
203. Petrić M, Lalic B, Ducheyne E *et al.* Modelling the regional impact of climate change on the suitability of the establishment of the Asian tiger mosquito (Aedes albopictus) in Serbia. *Climatic Change* 2017;**142**:361–74.
204. Lachlan M, Masahiro H, Ho K *et al.* Assessment of climate-sensitive infectious diseases in the Federated States of Micronesia. *Tropical Medicine and Health* 2014.
205. Cenni L, Simoncini A, Massetti L *et al.* Current and future distribution of a parasite with complex life cycle under global change scenarios: Echinococcus multilocularis in Europe. *Global Change Biology* 2023;**29**:2436–49.
206. Li S, Ma B, Di X *et al.* How climate, landscape, and economic changes increase the exposure of Echinococcus Spp. *Bmc Public Health* 2022;**22**:2315.
207. Restrepo AMC, Yang YR, McManus DP *et al.* Spatiotemporal patterns and environmental drivers of human echinococcoses over a twenty-year period in Ningxia Hui Autonomous Region, China. *Parasites and Vectors* 2018;**11**:108.
208. Botero-Cañola S, Dursahinhan AT, Rácz SE *et al.* The ecological niche of Echinococcus multilocularis in North America: Understanding biotic and abiotic determinants of parasite distribution with new records in New Mexico and Maryland, United States. *Therya* 2019;**10**:91–102.
209. Bargues MD, Artigas P, Angles R *et al.* Genetic uniformity, geographical spread and anthropogenic habitat modifications of lymnaeid vectors found in a One Health initiative in the highest human fascioliasis hyperendemic of the Bolivian Altiplano. *Parasit Vectors* 2020;**13**:171.
210. Bosco A, Rinaldi L, Musella V *et al.* Outbreak of acute fasciolosis in sheep farms in a Mediterranean area arising as a possible consequence of climate change. *Geospat Health* 2015;**9**:319–24.
211. Roldán C, Begovoeva M, López-Olvera JR *et al.* Endemic occurrence of Fasciola hepatica in an alpine ecosystem, Pyrenees, Northeastern Spain. *Transbound Emerg Dis* 2021;**68**:2589–94.
212. Salahi-Moghaddam A, Habibi-Nokhandam M, Fuentes MV. Low-altitude outbreaks of human fascioliasis related with summer rainfall in Gilan province, Iran. *Geospat Health* 2011;**6**:133–6.
213. Pedersen UB, Midzi N, Mduluza T *et al.* Modelling spatial distribution of snails transmitting parasitic worms with importance to human and animal health and analysis of distributional changes in relation to climate. *Geospat Health* 2014;**8**:335–43.
214. Relf V, Good B, Hanrahan JP *et al.* Temporal studies on Fasciola hepatica in Galba truncatula in the

west of Ireland. *Vet Parasitol* 2011;**175**:287–92.
215. Prasopdee S, Kulsantiwong J, Piratae S *et al.* Temperature dependence of Opisthorchis viverrini infection in first intermediate host snail, Bithynia siamensis goniomphalos. *Acta Trop* 2015;**141**:112–7.
216. Prasopdee S, Kulsantiwong J, Sathavornmanee T *et al.* The effects of temperature and salinity on the longevity of Opisthorchis viverrini cercariae: A climate change concern. *J Helminthol* 2020;**94**:e165.
217. Afshan K, Fortes-Lima CA, Artigas P *et al.* Impact of climate change and man-made irrigation systems on the transmission risk, long-term trend and seasonality of human and animal fascioliasis in Pakistan. *Geospat Health* 2014;**8**:317–34.
218. Caminade C, Dijk J van, Baylis M *et al.* Modelling recent and future climatic suitability for fasciolosis in Europe. *Geospat Health* 2015;**9**:301–8.
219. Fox NJ, White PC, McClean CJ *et al.* Predicting impacts of climate change on Fasciola hepatica risk. *Plos One* 2011;**6**:e16126.
220. Haydock LAJ, Pomroy WE, Stevenson MA *et al.* A growing degree-day model for determination of Fasciola hepatica infection risk in New Zealand with future predictions using climate change models. *Vet Parasitol* 2016;**228**:52–9.
221. Pedersen UB, Stendel M, Midzi N *et al.* Modelling climate change impact on the spatial distribution of fresh water snails hosting trematodes in Zimbabwe. *Parasit Vectors* 2014;**7**:536.
222. Suwannatrai A, Pratumchart K, Suwannatrai K *et al.* Modeling impacts of climate change on the potential distribution of the carcinogenic liver fluke, Opisthorchis viverrini, in Thailand. *Parasitol Res* 2017;**116**:243–50.
223. Li T, Yang Z, Wang M. Correlation between clonorchiasis incidences and climatic factors in Guangzhou, China. *Parasit Vectors* 2014;**7**:29.
224. Shrestha S, Barratt A, Fox NJ *et al.* Financial Impacts of Liver Fluke on Livestock Farms Under Climate Change-A Farm Level Assessment. *Front Vet Sci* 2020;**7**:564795.
225. Vilhena I, Martins F, Amaral GM *et al.* Climate change on the forecasted risk of bovine fasciolosis in Espírito Santo state, Brazil Mudanças climáticas e a previsão de risco futuro para fasciolose bovina no estado do Espírito Santo, Brasil. *NA* 2014;**NA**:NA–.
226. Nnko HJ, Gwakisa PS, Ngonyoka A *et al.* Potential impacts of climate change on geographical distribution of three primary vectors of African Trypanosomiasis in Tanzania's Maasai Steppe: G. M. Morsitans, G. Pallidipes and G. swynnertoni. *PLoS Negl Trop Dis* 2021;**15**:e0009081.
227. Kleynhans E, Terblanche JS. Complex Interactions between Temperature and Relative Humidity on Water Balance of Adult Tsetse (Glossinidae, Diptera): Implications for Climate Change. *Front Physiol* 2011;**2**:74.
228. Lord JS, Hargrove JW, Torr SJ *et al.* Climate change and African trypanosomiasis vector populations in Zimbabwe's Zambezi Valley: A mathematical modelling study. *PLoS Med* 2018;**15**:e1002675.
229. Messina JP, Moore NJ, DeVisser MH *et al.* Climate Change and Risk Projection: Dynamic Spatial Models of Tsetse and African Trypanosomiasis in Kenya. *Ann Assoc Am Geogr* 2012;**102**:1038–48.
230. Bishop AP, Amatulli G, Hyseni C *et al.* A machine learning approach to integrating genetic and ecological data in tsetse flies (Glossina pallidipes) for spatially explicit vector control planning. *Evol Appl* 2021;**14**:1762–77.
231. Longbottom J, Caminade C, Gibson HS *et al.* Modelling the impact of climate change on the distribution and abundance of tsetse in Northern Zimbabwe. *Parasit Vectors* 2020;**13**:526.
232. Moore N, Messina J. A landscape and climate data logistic model of tsetse distribution in Kenya. *Plos One* 2010;**5**:e11809.
233. Moore SM, Shrestha S, Tomlinson KW *et al.* Predicting the effect of climate change on African trypanosomiasis: Integrating epidemiology with parasite and vector biology. *Journal of the Royal Society, Interface* 2011;**9**:817–30.
234. Acosta LA, Mondragón-Shem K, Vergara D *et al.* [Expansion of the distribution of Lutzomyia longipalpis (Lutz & Neiva, 1912) (Diptera: Psychodidae) in the department of Caldas: Increased risk of visceral leishmaniasis]. *Biomedica* 2013;**33**:319–25.
235. Bastola A, Shrestha M, Lamsal M *et al.* A case of high altitude cutaneous leishmaniasis in a non-endemic region in Nepal. *Parasitol Int* 2020;**74**:101991.
236. Michelutti A, Toniolo F, Bertola M *et al.* Occurrence of Phlebotomine sand flies (Diptera: Psychodidae) in the northeastern plain of Italy. *Parasit Vectors* 2021;**14**:164.

237. Saadene Y, Salhi A, Mliki F *et al.* Climate change and cutaneous leishmaniasis in the province of Ghardaïa in Algeria: A model-based approach to predict disease outbreaks. *Ann Saudi Med* 2023;**43**:263–76.
238. Thomaz-Soccol V, Gonçalves AL, Baggio RA *et al.* One piece of the puzzle: Modeling vector presence and environment reveals seasonality, distribution, and prevalence of sandflies and Leishmania in an expansion area. *One Health* 2023;**17**:100581.
239. Castillo-Castañeda A, Herrera G, Ayala MS *et al.* Spatial and temporal variability of visceral leishmaniasis in Colombia, 2007 to 2018. *American Journal of Tropical Medicine and Hygiene* 2021;**105**:144–55.
240. Schaffner F, Silaghi C, Verhulst NO *et al.* The Phlebotomine sand fly fauna of Switzerland revisited. *Medical and Veterinary Entomology* 2023;**38**:13–22.
241. Kniha E, Dvorak V, Milchram M *et al.* Phlebotomus (Adlerius) simici NITZULESCU, 1931: First record in Austria and phylogenetic relationship with other Adlerius species. *Parasites and Vectors* 2021;**14**:20.
242. Raina S, Raina RK, Sharma R *et al.* Expansion of visceral leishmaniasis to northwest sub-himalayan region of India: A case series. *Journal of Vector Borne Diseases* 2016;**53**:188–91.
243. Gonzalez C, Paz A, Ferro C. Predicted altitudinal shifts and reduced spatial distribution of Leishmania infantum vector species under climate change scenarios in Colombia. *Acta Trop* 2014;**129**:83–90.
244. Cazan CD, Horváth C, Panait LC *et al.* Seasonal dynamics of Phlebotomus neglectus (Diptera: Psychodidae) in cave microhabitats in Romania and the rediscovery of Sergentomyia minuta (Rondani, 1843) after 50 years. *Parasit Vectors* 2021;**14**:476.
245. Díaz-Sáez V, Corpas-López V, Merino-Espinosa G *et al.* Seasonal dynamics of phlebotomine sand flies and autochthonous transmission of Leishmania infantum in high-altitude ecosystems in southern Spain. *Acta Trop* 2021;**213**:105749.
246. Ghatee MA, Sharifi I, Haghdoost AA *et al.* Spatial correlations of population and ecological factors with distribution of visceral leishmaniasis cases in southwestern Iran. *J Vector Borne Dis* 2013;**50**:179–87.
247. Kavur H. Modeling the Ecological Niche: A Case Study on Bioclimatic Factors Related to the Distribution of Phlebotomus tobbi Adler & Theodor (Diptera: Psychodidae) in Two Endemic Foci of Adana. *J Med Entomol* 2019;**56**:690–6.
248. Bounoua L, Kahime K, Houti L *et al.* Linking climate to incidence of zoonotic cutaneous leishmaniasis (L. Major) in pre-Saharan North Africa. *Int J Environ Res Public Health* 2013;**10**:3172–91.
249. Hlavacova J, Votypka J, Volf P. The effect of temperature on Leishmania (Kinetoplastida: Trypanosomatidae) development in sand flies. *J Med Entomol* 2013;**50**:955–8.
250. Carvalho BM, Rangel EF, Ready PD *et al.* Ecological Niche Modelling Predicts Southward Expansion of Lutzomyia (Nyssomyia) flaviscutellata (Diptera: Psychodidae: Phlebotominae), Vector of Leishmania (Leishmania) amazonensis in South America, under Climate Change. *Plos One* 2015;**10**:e0143282.
251. Chalghaf B, Chemkhi J, Mayala B *et al.* Ecological niche modeling predicting the potential distribution of Leishmania vectors in the Mediterranean basin: Impact of climate change. *Parasit Vectors* 2018;**11**:461.
252. Gonzalez C, Wang O, Strutz SE *et al.* Climate change and risk of leishmaniasis in north america: Predictions from ecological niche models of vector and reservoir species. *PLoS Negl Trop Dis* 2010;**4**:e585.
253. Purse BV, Masante D, Golding N *et al.* How will climate change pathways and mitigation options alter incidence of vector-borne diseases? A framework for leishmaniasis in South and Meso-America. *Plos One* 2017;**12**:e0183583.
254. Amro A, Moskalenko O, Hamarsheh O *et al.* Spatiotemporal analysis of cutaneous leishmaniasis in Palestine and foresight study by projections modelling until 2060 based on climate change prediction. *Plos One* 2022;**17**:e0268264.
255. Bede-Fazekas Á, Trájer A. Potential urban distribution of Phlebotomus mascittii Grassi and Phlebotomus neglectus Tonn. (Diptera: Psychodidae) in 2021-50 in Budapest, Hungary. *J Vector Borne Dis* 2015;**52**:213–8.
256. Bozorg-Omid F, Kafash A, Jafari R *et al.* Predicting current and future high-risk areas for vectors and reservoirs of cutaneous leishmaniasis in Iran. *Sci Rep* 2023;**13**:11546.
257. Daoudi M, Outammassine A, Amane M *et al.* Climate Change Influences on the Potential Distribution of the Sand Fly Phlebotomus sergenti, Vector of Leishmania tropica in Morocco. *Acta Parasitol* 2022;**67**:858–66.
258. Fischer D, Moeller P, Thomas SM *et al.* Combining climatic projections and dispersal ability: A method for estimating the responses of sandfly vector species to climate change. *PLoS Negl Trop Dis* 2011;**5**:e1407.

259. Fischer D, Thomas SM, Beierkuhnlein C. Temperature-derived potential for the establishment of phlebotomine sandflies and visceral leishmaniasis in Germany. *Geospat Health* 2010;**5**:59–69.
260. Gálvez R, Descalzo MA, Guerrero I *et al.* Mapping the current distribution and predicted spread of the leishmaniosis sand fly vector in the madrid region (Spain) based on environmental variables and expected climate change. *Vector Borne Zoonotic Dis* 2011;**11**:799–806.
261. Koch LK, Kochmann J, Klimpel S *et al.* Modeling the climatic suitability of leishmaniasis vector species in Europe. *Sci Rep* 2017;**7**:13325.
262. McIntyre S, Rangel EF, Ready PD *et al.* Species-specific ecological niche modelling predicts different range contractions for Lutzomyia intermedia and a related vector of Leishmania braziliensis following climate change in South America. *Parasit Vectors* 2017;**10**:157.
263. Medenica S, Miladinović-Tasić N, Stojanović NM *et al.* Climate Variables Related to the Incidence of Human Leishmaniosis in Montenegro in Southeastern Europe during Seven Decades (1945-2014). *Int J Environ Res Public Health* 2023;**20**, DOI: 10.3390/ijerph20031656.
264. Mendes CS, Coelho AB, Féres JG *et al.* [The impact of climate change on leishmaniasis in Brazil]. *Cien Saude Colet* 2016;**21**:263–72.
265. Moirano G, Ellena M, Mercogliano P *et al.* Spatio-Temporal Pattern and Meteo-Climatic Determinants of Visceral Leishmaniasis in Italy. *Trop Med Infect Dis* 2022;**7**, DOI: 10.3390/tropicalmed7110337.
266. Moo-Llanes D, Ibarra-Cerdeña CN, Rebollar-Téllez EA *et al.* Current and future niche of North and Central American sand flies (Diptera: Psychodidae) in climate change scenarios. *PLoS Negl Trop Dis* 2013;**7**:e2421.
267. Moo-Llanes DA. [Current and future ecological niche of Leishmaniasis (Kinetoplastida: Trypanosomatidae) in the Neotropical region]. *Rev Biol Trop* 2016;**64**:1237–45.
268. Moo-Llanes DA, Arque-Chunga W, Carmona-Castro O *et al.* Shifts in the ecological niche of Lutzomyia peruensis under climate change scenarios in Peru. *Med Vet Entomol* 2017;**31**:123–31.
269. Moo-Llanes DA, Pech-May A, Ibarra-Cerdeña CN *et al.* Inferring distributional shifts of epidemiologically important North and Central American sandflies from Pleistocene to future scenarios. *Med Vet Entomol* 2019;**33**:31–43.
270. Peterson AT, Campbell LP, Moo-Llanes DA *et al.* Influences of climate change on the potential distribution of Lutzomyia longipalpis sensu lato (Psychodidae: Phlebotominae). *Int J Parasitol* 2017;**47**:667–74.
271. Ramezankhani R, Hosseini A, Sajjadi N *et al.* Environmental risk factors for the incidence of cutaneous leishmaniasis in an endemic area of Iran: A GIS-based approach. *Spat Spatiotemporal Epidemiol* 2017;**21**:57–66.
272. Ramezankhani R, Sajjadi N, Nezakati Esmaeilzadeh R *et al.* Climate and environmental factors affecting the incidence of cutaneous leishmaniasis in Isfahan, Iran. *Environ Sci Pollut Res Int* 2018;**25**:11516–26.
273. Sevá AD, Mao L, Galvis-Ovallos F *et al.* Risk analysis and prediction of visceral leishmaniasis dispersion in São Paulo State, Brazil. *PLoS Negl Trop Dis* 2017;**11**:e0005353.
274. Trajer AJ. The potential future change of the suitability patterns of six leishmaniasis vectors in Iran. *J Vector Borne Dis* 2021;**58**:335–45.
275. Trájer AJ. The alteration of the suitability patterns of Leishmania infantum due to climate change in Iran. *Int J Environ Health Res* 2022;**32**:1567–80.
276. Wijerathna T, Gunathilaka N. Time series analysis of leishmaniasis incidence in Sri Lanka: Evidence for humidity-associated fluctuations. *Int J Biometeorol* 2023;**67**:275–84.
277. Zhao Y, Jiang D, Ding F *et al.* Recurrence and Driving Factors of Visceral Leishmaniasis in Central China. *Int J Environ Res Public Health* 2021;**18**, DOI: 10.3390/ijerph18189535.
278. Carvalho BM de, Perez LP, Oliveira BFA de *et al.* Vector-borne diseases in Brazil: Climate change and future global warming scenarios. *Sustentabilidade em Debate* 2020;**11**:361–82.
279. Shiravand B, Hanafi-Bojd AA, Tafti AAD *et al.* Climate change and potential distribution of zoonotic cutaneous leishmaniasis in Central Iran: Horizon 2030 and 2050. *Asian Pacific Journal of Tropical Medicine* 2019;**12**:204–15.
280. Trájer AJ, Bede-Fazekas Á, Hufnagel L *et al.* The effect of climate change on the potential distribution of the European Phlebotomus species. *Applied Ecology and Environmental Research* 2013;**11**:189–208.
281. Charrahy Z, Yaghoobi-Ershadi MR, Shirzadi MR *et al.* Climate change and its effect on the vulnerability to zoonotic cutaneous leishmaniasis in Iran. *Transboundary and Emerging Diseases* 2021;**69**:1506–20.

282. Al-Obaidi MJ, Ali HB. Effect of Climate Change on the Distribution of Zoonotic Cutaneous Leishmaniasis in Iraq. *Journal of Physics: Conference Series* 2021;**1818**:012052–NA.
283. Trájer AJ. The potential impact of climate change on the seasonality of Phlebotomus neglectus, the vector of visceral leishmaniasis in the East Mediterranean region. *International Journal of Environmental Health Research* 2019;**31**:1–19.
284. Trájer AJ, Grmasha RA. The potential effects of climate change on the climatic suitability patterns of the Western Asian vectors and parasites of cutaneous leishmaniasis in the mid- and late twenty-first century. *Theoretical and Applied Climatology* 2023;**NA**:NA–.
285. Boerlijst SP, Johnston ES, Ummels A *et al.* Biting the hand that feeds: Anthropogenic drivers interactively make mosquitoes thrive. *Sci Total Environ* 2023;**858**:159716.
286. Reisen WK, Thiemann T, Barker CM *et al.* Effects of warm winter temperature on the abundance and gonotrophic activity of Culex (Diptera: Culicidae) in California. *J Med Entomol* 2010;**47**:230–7.
287. Arjunan N, Kadarkari M, Pari M *et al.* Impact of climate change on filarial vector, Culex quinquefasciatus and control using bacterial pesticide, spinosad. *Asian Pacific Journal of Tropical Disease* 2014;**4**:S87–91.
288. Samy AM, Elaagip AH, Kenawy MA *et al.* Climate Change Influences on the Global Potential Distribution of the Mosquito Culex quinquefasciatus, Vector of West Nile Virus and Lymphatic Filariasis. *Plos One* 2016;**11**:e0163863.
289. Slater H, Michael E. Predicting the current and future potential distributions of lymphatic filariasis in Africa using maximum entropy ecological niche modelling. *Plos One* 2012;**7**:e32202.
290. Slater H, Michael E. Mapping, bayesian geostatistical analysis and spatial prediction of lymphatic filariasis prevalence in Africa. *Plos One* 2013;**8**:e71574.
291. Liu B, Gao X, Zheng K *et al.* The potential distribution and dynamics of important vectors Culex pipiens pallens and Culex pipiens quinquefasciatus in China under climate change scenarios: An ecological niche modelling approach. *Pest Manag Sci* 2020;**76**:3096–107.
292. Morin CW, Comrie AC. Modeled response of the West Nile virus vector Culex quinquefasciatus to changing climate using the dynamic mosquito simulation model. *Int J Biometeorol* 2010;**54**:517–29.
293. Balikuddembe JK, Reinhardt JD, Zeng W *et al.* Public health priorities for Sino-Africa cooperation in Eastern Africa in context of flooding and malaria burden in Children: A tridecadal retrospective analysis. *Bmc Public Health* 2023;**23**:1331.
294. Wang Z, Liu Y, Wang G *et al.* The relationship between rising temperatures and malaria incidence in Hainan, China, from 1984 to 2010: A longitudinal cohort study. *The Lancet Planetary Health* 2022;**6**:e350–8.
295. Wolfarth BR, Filizola N, Tadei WP *et al.* Epidemiological analysis of malaria and its relationships with hydrological variables in four municipalities of the State of Amazonas, Brazil. *Hydrological Sciences Journal* 2013;**58**:1495–504.
296. Bhandari GP, Dhimal M, Gurung S *et al.* Climate change and malaria in Jhapa district of Nepal: Emerging evidences from Nepal. *Journal of Health Management* 2013;**15**:141–50.
297. Gone T, Balkew M, Gebre-Michael T. Comparative entomological study on ecology and behaviour of Anopheles mosquitoes in highland and lowland localities of Derashe District, southern Ethiopia. *Parasit Vectors* 2014;**7**:483.
298. Dantur Juri MJ, Claps GL, Santana M *et al.* Abundance patterns of Anopheles pseudopunctipennis and Anopheles argyritarsis in northwestern Argentina. *Acta Trop* 2010;**115**:234–41.
299. Kulkarni MA, Desrochers RE, Kajeguka DC *et al.* 10 Years of Environmental Change on the Slopes of Mount Kilimanjaro and Its Associated Shift in Malaria Vector Distributions. *Front Public Health* 2016;**4**:281.
300. Lindsay SW, Jawara M, Mwesigwa J *et al.* Reduced mosquito survival in metal-roof houses may contribute to a decline in malaria transmission in sub-Saharan Africa. *Sci Rep* 2019;**9**:7770.
301. Mihailović DT, Petrić D, Petrović T *et al.* Assessment of climate change impact on the malaria vector Anopheles hyrcanus, West Nile disease, and incidence of melanoma in the Vojvodina Province (Serbia) using data from a regional climate model. *Plos One* 2020;**15**:e0227679.
302. Paaijmans KP, Imbahale SS, Thomas MB *et al.* Relevant microclimate for determining the development rate of malaria mosquitoes and possible implications of climate change. *Malar J* 2010;**9**:196.
303. Roiz D, Ruiz S, Soriguer R *et al.* Climatic effects on mosquito abundance in Mediterranean wetlands. *Parasit Vectors* 2014;**7**:333.
304. Yamana TK, Eltahir EA. Incorporating the effects of humidity in a mechanistic model of Anopheles gambiae mosquito population dynamics in the Sahel region of Africa. *Parasit Vectors* 2013;**6**:235.

305. Karypidou MC, Almpanidou V, Tompkins AM *et al.* Projected shifts in the distribution of malaria vectors due to climate change. *Climatic Change* 2020;**163**:2117–33.
306. Chuang TW, Soble A, Ntshalintshali N *et al.* Assessment of climate-driven variations in malaria incidence in Swaziland: Toward malaria elimination. *Malaria Journal* 2017;**16**:232.
307. Boyce R, Reyes R, Matte M *et al.* Severe Flooding and Malaria Transmission in the Western Ugandan Highlands: Implications for Disease Control in an Era of Global Climate Change. *Journal of Infectious Diseases* 2016;**214**:1403–10.
308. Zhang Y, Liu QY, Luan RS *et al.* Spatial-temporal analysis of malaria and the effect of environmental factors on its incidence in Yongcheng, China, 2006-2010. *Bmc Public Health* 2012;**12**:544.
309. Fletcher IK, Stewart-Ibarra AM, Sippy R *et al.* The Relative Role of Climate Variation and Control Interventions on Malaria Elimination Efforts in El Oro, Ecuador: A Modeling Study. *Frontiers in Environmental Science* 2020;**8**, DOI: 10.3389/fenvs.2020.00135.
310. Mattah PAD, Futagbi G, Amekudzi LK *et al.* Climate variations, urban solid waste management and possible implications for anopheles mosquito breeding in selected cities of coastal ghana. *West African Journal of Applied Ecology* 2020;**28**:21–34.
311. Abrha H, Hagos H, Brhane E *et al.* Spatio-temporal dynamics of malaria expansion under climate change in semi-arid areas of Ethiopia. *Environmental Hazards* 2019;**18**:400–13.
312. Adu-Prah S, Kofi Tetteh E. Spatiotemporal analysis of climate variability impacts on malaria prevalence in Ghana. *Applied Geography* 2015;**60**:266–73.
313. Caminade C, Ndione J-A, Kebe CMF *et al.* Mapping Rift Valley fever and malaria risk over West Africa using climatic indicators. *Atmospheric Science Letters* 2010;**12**:96–103.
314. Agyekum TP, Arko-Mensah J, Botwe PK *et al.* Effects of elevated temperatures on the development of immature stages of Anopheles gambiae (s.l.) mosquitoes. *Trop Med Int Health* 2022;**27**:338–46.
315. Chu VM, Sallum MAM, Moore TE *et al.* Evidence for family-level variation of phenotypic traits in response to temperature of Brazilian Nyssorhynchus darlingi. *Parasit Vectors* 2020;**13**:55.
316. Glunt KD, Oliver SV, Hunt RH *et al.* The impact of temperature on insecticide toxicity against the malaria vectors Anopheles arabiensis and Anopheles funestus. *Malar J* 2018;**17**:131.
317. Lyons CL, Coetzee M, Chown SL. Stable and fluctuating temperature effects on the development rate and survival of two malaria vectors, Anopheles arabiensis and Anopheles funestus. *Parasit Vectors* 2013;**6**:104.
318. Lyons CL, Coetzee M, Terblanche JS *et al.* Thermal limits of wild and laboratory strains of two African malaria vector species, Anopheles arabiensis and Anopheles funestus. *Malar J* 2012;**11**:226.
319. Murdock CC, Blanford S, Hughes GL *et al.* Temperature alters Plasmodium blocking by Wolbachia. *Sci Rep* 2014;**4**:3932.
320. Murdock CC, Sternberg ED, Thomas MB. Malaria transmission potential could be reduced with current and future climate change. *Sci Rep* 2016;**6**:27771.
321. Nwaefuna EK, Bagshaw, Gbogbo F *et al.* Oviposition and Development of Anopheles coluzzii coetzee and Wilkerson in Salt Water. *Malar Res Treat* 2019;**2019**:9523962.
322. Oliver SV, Brooke BD. The effect of elevated temperatures on the life history and insecticide resistance phenotype of the major malaria vector Anopheles arabiensis (Diptera: Culicidae). *Malar J* 2017;**16**:73.
323. Owusu HF, Chitnis N, Müller P. Insecticide susceptibility of Anopheles mosquitoes changes in response to variations in the larval environment. *Sci Rep* 2017;**7**:3667.
324. Waite JL, Suh E, Lynch PA *et al.* Exploring the lower thermal limits for development of the human malaria parasite, Plasmodium falciparum. *Biol Lett* 2019;**15**:20190275.
325. Paaijmans KP, Blanford S, Bell AS *et al.* Influence of climate on malaria transmission depends on daily temperature variation. *Proceedings of the National Academy of Sciences of the United States of America* 2010;**107**:15135–9.
326. Paaijmans KP, Blanford S, Chan BHK *et al.* Warmer temperatures reduce the vectorial capacity of malaria mosquitoes. *Biology Letters* 2012;**8**:465–8.
327. Akpan GE, Adepoju KA, Oladosu OR. Potential distribution of dominant malaria vector species in tropical region under climate change scenarios. *Plos One* 2019;**14**:e0218523.
328. Alimi TO, Fuller DO, Qualls WA *et al.* Predicting potential ranges of primary malaria vectors and malaria in northern South America based on projected changes in climate, land cover and human population. *Parasit Vectors* 2015;**8**:431.

329. Beck-Johnson LM, Nelson WA, Paaijmans KP *et al.* The effect of temperature on Anopheles mosquito population dynamics and the potential for malaria transmission. *Plos One* 2013;**8**:e79276.
330. Beck-Johnson LM, Nelson WA, Paaijmans KP *et al.* The importance of temperature fluctuations in understanding mosquito population dynamics and malaria risk. *R Soc Open Sci* 2017;**4**:160969.
331. Bhattarai S, Blackburn JK, Ryan SJ. Malaria transmission in Nepal under climate change: Anticipated shifts in extent and season, and comparison with risk definitions for intervention. *Malar J* 2022;**21**:390.
332. Carlson CJ, Bannon E, Mendenhall E *et al.* Rapid range shifts in African Anopheles mosquitoes over the last century. *Biol Lett* 2023;**19**:20220365.
333. Chaturvedi S, Dwivedi S. Estimating the malaria transmission over the Indian subcontinent in a warming environment using a dynamical malaria model. *J Water Health* 2020;**18**:358–74.
334. Chaturvedi S, Dwivedi S. Understanding the effect of climate change in the distribution and intensity of malaria transmission over India using a dynamical malaria model. *Int J Biometeorol* 2021;**65**:1161–75.
335. Chen TT, Ljungqvist FC, Castenbrandt H *et al.* The spatiotemporal distribution of historical malaria cases in Sweden: A climatic perspective. *Malar J* 2021;**20**:212.
336. Drake JM, Beier JC. Ecological niche and potential distribution of Anopheles arabiensis in Africa in 2050. *Malar J* 2014;**13**:213.
337. Edlund S, Davis M, Douglas JV *et al.* A global model of malaria climate sensitivity: Comparing malaria response to historic climate data based on simulation and officially reported malaria incidence. *Malar J* 2012;**11**:331.
338. Fall P, Diouf I, Deme A *et al.* Bias-Corrected CMIP5 Projections for Climate Change and Assessments of Impact on Malaria in Senegal under the VECTRI Model. *Trop Med Infect Dis* 2023;**8**, DOI: 10.3390/tropicalmed8060310.
339. Hanafi-Bojd AA, Vatandoost H, Yaghoobi-Ershadi MR. Climate Change and the Risk of Malaria Transmission in Iran. *J Med Entomol* 2020;**57**:50–64.
340. Hertig E. Distribution of Anopheles vectors and potential malaria transmission stability in Europe and the Mediterranean area under future climate change. *Parasit Vectors* 2019;**12**:18.
341. Holy M, Schmidt G, Schröder W. Potential malaria outbreak in Germany due to climate warming: Risk modelling based on temperature measurements and regional climate models. *Environ Sci Pollut Res Int* 2011;**18**:428–35.
342. Hurtado LA, Calzada JE, Rigg CA *et al.* Climatic fluctuations and malaria transmission dynamics, prior to elimination, in Guna Yala, República de Panamá. *Malar J* 2018;**17**:85.
343. Khormi HM, Kumar L. Future malaria spatial pattern based on the potential global warming impact in South and Southeast Asia. *Geospat Health* 2016;**11**:416.
344. Kumar P, Vatsa R, Sarthi PP *et al.* Modeling an association between malaria cases and climate variables for Keonjhar district of Odisha, India: A Bayesian approach. *J Parasit Dis* 2020;**44**:319–31.
345. Laporta GZ, Linton YM, Wilkerson RC *et al.* Malaria vectors in South America: Current and future scenarios. *Parasit Vectors* 2015;**8**:426.
346. Le PVV, Kumar P, Ruiz MO *et al.* Predicting the direct and indirect impacts of climate change on malaria in coastal Kenya. *Plos One* 2019;**14**:e0211258.
347. Li C, Gao Y, Chang N *et al.* Risk Assessment of Anopheles philippinensis and Anopheles nivipes (Diptera: Culicidae) Invading China under Climate Change. *Biology (Basel)* 2021;**10**, DOI: 10.3390/biology10100998.
348. Lindsay SW, Hole DG, Hutchinson RA *et al.* Assessing the future threat from vivax malaria in the United Kingdom using two markedly different modelling approaches. *Malar J* 2010;**9**:70.
349. Marques R, Krüger RF, Cunha SK *et al.* Climate change impacts on Anopheles (K.) cruzii in urban areas of Atlantic Forest of Brazil: Challenges for malaria diseases. *Acta Trop* 2021;**224**:106123.
350. Moukam Kakmeni FM, Guimapi RYA, Ndjomatchoua FT *et al.* Spatial panorama of malaria prevalence in Africa under climate change and interventions scenarios. *Int J Health Geogr* 2018;**17**:2.
351. Nejati J, Vatandoost H, Baygi MZ *et al.* Predicting the Potential Distribution of Major Malaria Vectors Based on Climate Changes in Sistan and Baluchistan Province, Southeastern Iran. *J Arthropod Borne Dis* 2021;**15**:300–13.
352. Ngarakana-Gwasira ET, Bhunu CP, Masocha M *et al.* Assessing the Role of Climate Change in Malaria Transmission in Africa. *Malar Res Treat* 2016;**2016**:7104291.
353. Nili S, Asadgol Z, Dalaei H *et al.* The effect of climate change on malaria transmission in the southeast

of Iran. *Int J Biometeorol* 2022;**66**:1613–26.
354. Ryan SJ, Lippi CA, Villena OC *et al.* Mapping current and future thermal limits to suitability for malaria transmission by the invasive mosquito Anopheles stephensi. *Malar J* 2023;**22**:104.
355. Ryan SJ, Lippi CA, Zermoglio F. Shifting transmission risk for malaria in Africa with climate change: A framework for planning and intervention. *Malar J* 2020;**19**:170.
356. Ryan SJ, McNally A, Johnson LR *et al.* Mapping Physiological Suitability Limits for Malaria in Africa Under Climate Change. *Vector Borne Zoonotic Dis* 2015;**15**:718–25.
357. Saberi N, Raeisi A, Gorouhi MA *et al.* Modeling the Effect of Climate Change on the Distribution of Main Malaria Vectors in an Endemic Area, Southeastern Iran. *Iran J Public Health* 2023;**52**:1061–70.
358. Salahi-Moghaddam A, Khoshdel A, Dalaei H *et al.* Spatial changes in the distribution of malaria vectors during the past 5 decades in Iran. *Acta Trop* 2017;**166**:45–53.
359. Santos-Vega M, Martinez PP, Vaishnav KG *et al.* The neglected role of relative humidity in the interannual variability of urban malaria in Indian cities. *Nat Commun* 2022;**13**:533.
360. Siya A, Kalule BJ, Ssentongo B *et al.* Malaria patterns across altitudinal zones of Mount Elgon following intensified control and prevention programs in Uganda. *BMC Infect Dis* 2020;**20**:425.
361. Tonnang HE, Tchouassi DP, Juarez HS *et al.* Zoom in at African country level: Potential climate induced changes in areas of suitability for survival of malaria vectors. *Int J Health Geogr* 2014;**13**:12.
362. Valderrama L, Ayala S, Reyes C *et al.* Modeling the Potential Distribution of the Malaria Vector Anopheles (Ano.) pseudopunctipennis Theobald (Diptera: Culicidae) in Arid Regions of Northern Chile. *Front Public Health* 2021;**9**:611152.
363. Wang Y, Rao Y, Wu X *et al.* A method for screening climate change-sensitive infectious diseases. *Int J Environ Res Public Health* 2015;**12**:767–83.
364. Wangdi K, Singhasivanon P, Silawan T *et al.* Development of temporal modelling for forecasting and prediction of malaria infections using time-series and ARIMAX analyses: A case study in endemic districts of Bhutan. *Malar J* 2010;**9**:251.
365. Tonnang HEZ, Kangalawe RYM, Yanda PZ. Review Predicting and mapping malaria under climate change scenarios: The potential redistribution of malaria vectors in Africa. *Malaria Journal* 2010;**9**:111.
366. Schröder W, Holy M, Pesch R *et al.* Climate change and future potential temperature dependent malaria transmission gates. *Umweltwissenschaften und Schadstoff-Forschung* 2010;**22**:177–87.
367. Bomblies A, Eltahir EAB. Assessment of the Impact of Climate Shifts on Malaria Transmission in the Sahel. *Ecohealth* 2010;**6**:1–12.
368. Li C, Gao Y, Zhao Z *et al.* Potential geographical distribution of Anopheles gambiae worldwide under climate change. *Journal of Biosafety and Biosecurity* 2021;**3**:125–30.
369. Mordecai EA, Paaijmans KP, Johnson LR *et al.* Optimal temperature for malaria transmission is dramatically lower than previously predicted. *Ecology Letters* 2013;**16**:22–30.
370. Chua TH. Modelling the effect of temperature change on the extrinsic incubation period and reproductive number of Plasmodium falciparum in Malaysia. *Tropical Biomedicine* 2012;**29**:121–8.
371. Gouda KC, Pernaje N, Benke M. Climate parameter and malaria association in north-east India. *Journal of Parasitic Diseases* 2023;**47**:501–12.
372. Woyessa A, Siebert A, Owusu A *et al.* El Nino and other climatic drivers of epidemic malaria in Ethiopia: New tools for national health adaptation plans. *Malaria Journal* 2023;**22**:195.
373. Asadgol Z, Badirzadeh A, Mirahmadi H *et al.* Simulation of the potential impact of climate change on malaria incidence using artificial neural networks (ANNs). *Environmental science and pollution research international* 2023;**30**:75349–68.
374. Sarkar S, Gangare V, Singh P *et al.* Shift in potential malaria transmission areas in india, using the fuzzy-based climate suitability malaria transmission (FCSMT) model under changing climatic conditions. *International Journal of Environmental Research and Public Health* 2019;**16**:3474.
375. Yaladanda N, Mopuri R, Vavilala H *et al.* The synergistic effect of climatic factors on malaria transmission: A predictive approach for northeastern states of India. *Environmental science and pollution research international* 2023;**30**:59194–211.
376. Nyawanda BO, Beloconi A, Khagayi S *et al.* The relative effect of climate variability on malaria incidence after scale-up of interventions in western Kenya: A time-series analysis of monthly incidence data from 2008 to 2019. *Parasite Epidemiology and Control* 2023;**21**:e00297.
377. Diouf I, Ndione JA, Gaye AT. Malaria in Senegal: Recent and Future Changes Based on Bias-Corrected

CMIP6 Simulations. *Tropical Medicine and Infectious Disease* 2022;**7**:345.
378. Mbouna AD, Tamoffo AT, Asare EO *et al.* Malaria metrics distribution under global warming: Assessment of the VECTRI malaria model over Cameroon. *International Journal of Biometeorology* 2023;**67**:93–105.
379. Mafwele BJ, Lee JW. Relationships between transmission of malaria in Africa and climate factors. *Scientific Reports* 2022;**12**:14392.
380. Wangdi K, Wetzler E, Cox H *et al.* Spatial patterns and climate drivers of malaria in three border areas of Brazil, Venezuela and Guyana, 2016-2018. *Scientific Reports* 2022;**12**:10995.
381. Parihar RS, Bal PK, Saini A *et al.* Potential future malaria transmission in Odisha due to climate change. *Scientific Reports* 2022;**12**:9048.
382. Li C, Managi S. Global malaria infection risk from climate change. *Environmental Research* 2022;**214**:114028.
383. Panzi EK, Kandala NII, Kafinga EL *et al.* Forecasting Malaria Morbidity to 2036 Based on Geo-Climatic Factors in the Democratic Republic of Congo. *International Journal of Environmental Research and Public Health* 2022;**19**:12271.
384. Panzi EK, Okenge LN, Kabali EH *et al.* Geo-Climatic Factors of Malaria Morbidity in the Democratic Republic of Congo from 2001 to 2019. *International Journal of Environmental Research and Public Health* 2022;**19**:3811.
385. Krsulovic FAM, Moulton TP, Lima M *et al.* Epidemic malaria dynamics in Ethiopia: The role of self-limiting, poverty, HIV, climate change and human population growth. *Malaria Journal* 2022;**21**:135.
386. Carlson CJ, Colwell R, Hossain MS *et al.* Solar geoengineering could redistribute malaria risk in developing countries. *Nature Communications* 2022;**13**:2150.
387. Nigussie TZ, Zewotir T, Muluneh EK. Effects of climate variability and environmental factors on the spatiotemporal distribution of malaria incidence in the Amhara national regional state, Ethiopia. *Spatial and Spatio-temporal Epidemiology* 2022;**40**:100475.
388. Ototo EN, Ogutu JO, Githeko A *et al.* Forecasting the Potential Effects of Climate Change on Malaria in the Lake Victoria Basin Using Regionalized Climate Projections. *Acta parasitologica* 2022;**67**:1535–63.
389. Chemison A, Ramstein G, Tompkins AM *et al.* Impact of an accelerated melting of Greenland on malaria distribution over Africa. *Nature Communications* 2021;**12**:3971.
390. Rodo X, Martinez PP, Siraj A *et al.* Malaria trends in Ethiopian highlands track the 2000 'slowdown' in global warming. *Nature Communications* 2021;**12**:1555.
391. Lubinda J, Haque U, Bi Y *et al.* Climate change and the dynamics of age-related malaria incidence in Southern Africa. *Environmental Research* 2021;**197**:111017.
392. Liu Z, Wang S, Xiang J *et al.* Effect of temperature and its interactions with relative humidity and rainfall on malaria in a temperate city Suzhou, China. *Environmental science and pollution research international* 2021;**28**:16830–42.
393. Carrasco-Escobar G, Qquellon J, Villa D *et al.* Time-Varying Effects of Meteorological Variables on Malaria Epidemiology in the Context of Interrupted Control Efforts in the Amazon Rainforest, 2000-2017. *Frontiers in Medicine* 2021;**8**:721515.
394. Segun OE, Shohaimi S, Nallapan M *et al.* Statistical modelling of the effects ofweather factors on malaria occurrence in Abuja, Nigeria. *International Journal of Environmental Research and Public Health* 2020;**17**:3474.
395. Azevedo TS de, Lorenz C, Chiaravalloti-Neto F *et al.* Kerteszia cruzii and extra-Amazonian malaria in Brazil: Challenges due to climate change in the Atlantic Forest. *Infection, Genetics and Evolution* 2020;**85**:104456.
396. Smith MW, Willis T, Alfieri L *et al.* Incorporating hydrology into climate suitability models changes projections of malaria transmission in Africa. *Nature Communications* 2020;**11**:4353.
397. Trajer AJ. The changing risk patterns of Plasmodium vivax malaria in Greece due to climate change. *International Journal of Environmental Health Research* 2020;**32**:1–26.
398. Mopuri R, Kakarla SG, Mutheneni SR *et al.* Climate based malaria forecasting system for Andhra Pradesh, India. *Journal of Parasitic Diseases* 2020;**44**:497–510.
399. Chirombo J, Ceccato P, Lowe R *et al.* Childhood malaria case incidence in Malawi between 2004 and 2017: Spatio-temporal modelling of climate and non-climate factors. *Malaria Journal* 2020;**19**:5.
400. Laneri K, Cabella B, Prado PI *et al.* Climate drivers of malaria at its southern fringe in the Americas.

Plos One 2019;**14**:e0219249.
401. Tourre YM, Vignolles C, Viel C *et al.* Malaria in Burkina Faso (West Africa) during the twenty-first century. *Environmental Monitoring and Assessment* 2019;**191**:273.
402. M'Bra RK, Kone B, Soro DP *et al.* Impact of climate variability on the transmission risk of malaria in northern Cote d'Ivoire. *Plos One* 2018;**13**:0182304.
403. Hundessa S, Li S, Liu DL *et al.* Projecting environmental suitable areas for malaria transmission in China under climate change scenarios. *Environmental Research* 2018;**162**:203–10.
404. Ishengoma DS, Mmbando BP, Mandara CI *et al.* Trends of Plasmodium falciparum prevalence in two communities of Muheza district North-eastern Tanzania: Correlation between parasite prevalence, malaria interventions and rainfall in the context of re-emergence of malaria after two decades of progressive. *Malaria Journal* 2018;**17**:252.
405. Hundessa S, Williams G, Li S *et al.* Projecting potential spatial and temporal changes in the distribution of Plasmodium vivax and Plasmodium falciparum malaria in China with climate change. *Science of the Total Environment* 2018;**627**:1285–93.
406. Ssempiira J, Kissa J, Nambuusi B *et al.* Interactions between climatic changes and intervention effects on malaria spatio-temporal dynamics in Uganda. *Parasite Epidemiology and Control* 2018;**3**:e00070.
407. Zhai JX, Lu Q, Hu WB *et al.* Development of an empirical model to predict malaria outbreaks based on monthly case reports and climate variables in Hefei, China, 1990-2011. *Acta Tropica* 2018;**178**:148–54.
408. Dasgupta S. Burden of climate change on malaria mortality. *International journal of hygiene and environmental health* 2018;**221**:782–91.
409. Diouf I, Rodriguez-Fonseca B, Deme A *et al.* Comparison of malaria simulations driven by meteorological observations and reanalysis products in Senegal. *International Journal of Environmental Research and Public Health* 2017;**14**:1119.
410. Sheikhzadeh K, Haghdoost AA, Bahrampour A *et al.* Predicting malaria transmission risk in endemic areas of iran: A multilevel modeling using climate and socioeconomic indicators. *Iranian Red Crescent Medical Journal* 2017;**19**, DOI: https://dx.doi.org/10.5812/ircmj.45132.
411. Song Y, Ge Y, Wang J *et al.* Spatial distribution estimation of malaria in northern China and its scenarios in 2020, 2030, 2040 and 2050. *Malaria Journal* 2016;**15**:345.
412. Mohammadkhani M, Khanjani N, Bakhtiari B *et al.* The relation between climatic factors and malaria incidence in Kerman, South East of Iran. *Parasite Epidemiology and Control* 2016;**1**:205–10.
413. Ostovar A, Haghdoost AA, Rahimiforoushani A *et al.* Time series analysis of meteorological factors influencing Malaria in south eastern Iran. *Journal of Arthropod-Borne Diseases* 2016;**10**:222–37.
414. Kibret S, Lautze J, McCartney M *et al.* Malaria and large dams in sub-Saharan Africa: Future impacts in a changing climate. *Malaria Journal* 2016;**15**:448.
415. Tompkins AM, Larsen L, McCreesh N *et al.* To what extent does climate explain variations in reported malaria cases in early 20th century Uganda? *Geospatial Health* 2016;**11**:407.
416. Leedale J, Tompkins AM, Caminade C *et al.* Projecting malaria hazard from climate change in eastern Africa using large ensembles to estimate uncertainty. *Geospatial Health* 2016;**11**:393.
417. Tompkins AM, Caporaso L. Assessment of malaria transmission changes in Africa, due to the climate impact of land use change using Coupled Model Intercomparison Project Phase 5 earth system models. *Geospatial Health* 2016;**11**:380.
418. Ren Z, Wang D, Ma A *et al.* Predicting malaria vector distribution under climate change scenarios in China: Challenges for malaria elimination. *Scientific Reports* 2016;**6**:20604.
419. Siraj AS, Santos-Vega M, Bouma MJ *et al.* Altitudinal changes in malaria incidence in highlands of Ethiopia and Colombia. *Science (New York, NY)* 2014;**343**:1154–8.
420. Kwak J, Noh H, Kim S *et al.* Future climate data from RCP 4.5 and occurrence of malaria in Korea. *International Journal of Environmental Research and Public Health* 2014;**11**:10587–605.
421. Caminade C, Kovats S, Rocklov J *et al.* Impact of climate change on global malaria distribution. *Proceedings of the National Academy of Sciences of the United States of America* 2014;**111**:3286–91.
422. Lowe R, Chirombo J, Tompkins AM. Relative importance of climatic, geographic and socio-economic determinants of malaria in Malawi. *Malaria Journal* 2013;**12**:416.
423. Yamana TK, Eltahir EAB. Projected impacts of climate change on environmental suitability for malaria transmission in West Africa. *Environmental Health Perspectives* 2013;**121**:1179–86.
424. Ermert V, Fink AH, Morse AP *et al.* The impact of regional climate change on malaria risk due to

greenhouse forcing and land-use changes in tropical Africa. *Environmental Health Perspectives* 2012;**120**:77–84.
425. Chaves LF, Hashizume M, Satake A *et al.* Regime shifts and heterogeneous trends in malaria time series from Western Kenya Highlands. *Parasitology* 2012;**139**:14–25.
426. Yang GJ, Tanner M, Utzinger J *et al.* Malaria surveillance-response strategies in different transmission zones of the People's Republic of China: Preparing for climate change. *Malaria Journal* 2012;**11**:426.
427. Kim YM, Park JW, Cheong HK. Estimated effect of climatic variables on the transmission of plasmodium vivax malaria in the republic of Korea. *Environmental Health Perspectives* 2012;**120**:1314–9.
428. Delgado-Petrocelli L, Cordova K, Camardiel A *et al.* Analysis of the El Nino/La nina-Southern oscillation variability and malaria in the Estado Sucre, Venezuela. *Geospatial Health* 2012;**6**:S51–7.
429. Egbendewe-Mondzozo A, Musumba M, McCarl BA *et al.* Climate change and vector-borne diseases: An economic impact analysis of malaria in Africa. *International Journal of Environmental Research and Public Health* 2011;**8**:913–30.
430. Omumbo JA, Lyon B, Waweru SM *et al.* Raised temperatures over the Kericho tea estates: Revisiting the climate in the East African highlands malaria debate. *Malaria Journal* 2011;**10**:12.
431. Nkurunziza H, Gebhardt A, Pilz J. Geo-additive modelling of malaria in Burundi. *Malaria Journal* 2011;**10**:234.
432. Alonso D, Bouma MJ, Pascual M. Epidemic malaria and warmer temperatures in recent decades in an East African highland. *Proceedings of the Royal Society B: Biological Sciences* 2011;**278**:1661–9.
433. Stern DI, Gething PW, Kabaria CW *et al.* Temperature and malaria trends in highland East Africa. *Plos One* 2011;**6**:e24524.
434. Nkurunziza H, Gebhardt A, Pilz J. Bayesian modelling of the effect of climate on malaria in Burundi. *Malaria Journal* 2010;**9**:114.
435. Zhang Y, Bi P, Hiller JE. Meteorological variables and malaria in a Chinese temperate city: A twenty-year time-series data analysis. *Environment International* 2010;**36**:439–45.
436. Yang GJ, Gao Q, Zhou SS *et al.* Mapping and predicting malaria transmission in the People's Republic of China, using integrated biology-driven and statistical models. *Geospatial Health* 2010;**5**:11–22.
437. Artzy-Randrup Y, Alonso D, Pascual M. Transmission intensity and drug resistance in malaria population dynamics: Implications for climate change. *Plos One* 2010;**5**:e13588.
438. Gething PW, Smith DL, Patil AP *et al.* Climate change and the global malaria recession. *Nature* 2010;**465**:342–5.
439. Parham PE, Michael E. Modeling the effects of weather and climate change on malaria transmission. *Environmental Health Perspectives* 2010;**118**:620–6.
440. Mironova V, Shartova N, Beljaev A *et al.* Effects of Climate Change and Heterogeneity of Local Climates n the Development of Malaria Parasite (Plasmodium vivax) in Moscow Megacity Region. *Int J Environ Res Public Health* 2019;**16**, DOI: 10.3390/ijerph16050694.
441. Nigussie TZ, Zewotir TT, Muluneh EK. Seasonal and spatial variations of malaria transmissions in northwest Ethiopia: Evaluating climate and environmental effects using generalized additive model. *Heliyon* 2023;**9**:e15252.
442. Ogega OM, Alobo M. Impact of 1.5 (o)C and 2 (o)C global warming scenarios on malaria transmission in East Africa. *AAS Open Res* 2020;**3**:22.
443. Park JW, Cheong HK, Honda Y *et al.* Time trend of malaria in relation to climate variability in Papua New Guinea. *Environ Health Toxicol* 2016;**31**:e2016003.
444. Tegegne E, Alemu Gelaye K, Dessie A *et al.* Spatio-Temporal Variation of Malaria Incidence and Risk Factors in West Gojjam Zone, Northwest Ethiopia. *Environ Health Insights* 2022;**16**:11786302221095702.
445. Tian H, Li N, Li Y *et al.* Malaria elimination on Hainan Island despite climate change. *Commun Med (Lond)* 2022;**2**:12.
446. Dwivedi S, Chaturvedi S. Multifractal analysis of malaria cases in India in a global warming scenario. *Journal of Water and Climate Change* 2023;**14**:1466–81.
447. Chaturvedi S, Dwivedi S. Impact of El Niño–Southern Oscillation and Indian Ocean Dipole on malaria transmission over India in changing climate. *International Journal of Environmental Science and Technology* 2023;**21**:91–100.
448. Liu X, Song C, Ren Z *et al.* Predicting the Geographical Distribution of Malaria-Associated Anopheles dirus in the South-East Asia and Western Pacific Regions Under Climate Change Scenarios. *Frontiers in*

Environmental Science 2022;**10**, DOI: 10.3389/fenvs.2022.841966.
449. Diouf I, Adeola AM, Abiodun GJ *et al.* Impact of future climate change on malaria in West Africa. *Theoretical and Applied Climatology* 2022;**147**:853–65.
450. Parihar RS, Bal PK, Thapliyal A *et al.* Climate Change Projections and its Impacts on Potential Malaria Transmission Dynamics in Uttarakhand. *Journal of Communicable Diseases* 2022;**54**:47–53.
451. Asori M, Musah A, Gyasi RM. Bio-climatic impact on malaria prevalence in Ghana: A multi-scale spatial modeling. *African Geographical Review* 2022:1–22.
452. Kimuyu JS. Comparative spatial–temporal analysis and predictive modeling of climate change-induced malaria vectors' invasion in new hotspots in Kenya. *SN Applied Sciences* 2021;**3**, DOI: 10.1007/s42452-021-04722-1.
453. Endo N, Eltahir EAB. Increased risk of malaria transmission with warming temperature in the Ethiopian Highlands. *Environmental Research Letters* 2020;**15**, DOI: 10.1088/1748-9326/ab7520.
454. Mohammadkhani M, Khanjani N, Bakhtiari B *et al.* The Relation Between Climatic Factors and Malaria Incidence in Sistan and Baluchestan, Iran. *SAGE Open* 2019;**9**, DOI: 10.1177/2158244019864205.
455. Lyon B, Dinku T, Raman A *et al.* Temperature suitability for malaria climbing the Ethiopian Highlands. *Environmental Research Letters* 2017;**12**, DOI: 10.1088/1748-9326/aa64e6.
456. Bouma MJ, Siraj AS, Rodo X *et al.* El Niño-based malaria epidemic warning for Oromia, Ethiopia, from August 2016 to July 2017. *Tropical Medicine and International Health* 2016;**21**:1481–8.
457. Trájer A, Hammer T. Climate-based seasonality model of temperate malaria based on the epidemiological data of 1927–1934, Hungary. *Idojaras* 2016;**120**:331–51.
458. Taye G, Kaba M, Woyessa A *et al.* Modeling effect of climate variability on malaria in Ethiopia. *Ethiopian Journal of Health Development* 2015;**29**:183–96.
459. Paaijmans KP, Blanford JI, Crane RG *et al.* Downscaling reveals diverse effects of anthropogenic climate warming on the potential for local environments to support malaria transmission. *Climatic Change* 2014;**125**:479–88.
460. Ermert V, Fink AH, Paeth H. The potential effects of climate change on malaria transmission in Africa using bias-corrected regionalised climate projections and a simple malaria seasonality model. *Climatic Change* 2013;**120**:741–54.
461. Dhiman RC, Chavan L, Pant M *et al.* National and regional impacts of climate change on malaria by 2030. *Current Science* 2011;**101**:372–83.
462. Béguin A, Hales S, Rocklöv J *et al.* The opposing effects of climate change and socio-economic development on the global distribution of malaria. *Global Environmental Change* 2011;**21**:1209–14.
463. Nkurunziza H, Pilz J. Impact of increased temperature on malaria transmission in Burundi. *International Journal of Global Warming* 2011;**3**:77–87.
464. Ohta S, Kaga T. Possible effects of future climate changes on the maximum number of generations of anopheles in monsoon asia. In: Blanco J, Kheradmand H (eds.). *Climate Change.* Rijeka: IntechOpen, 2011.
465. Cheke RA, Post RJ, Boakye DA. Seasonal variations and other changes in the geographical distributions of different cytospecies of the Simulium damnosum complex (Diptera: Simuliidae) in Togo and Benin. *Acta Trop* 2023;**245**:106970.
466. Cheke RA, Basanez MG, Perry M *et al.* Potential effects of warmer worms and vectors on onchocerciasis transmission in West Africa. *Philos Trans R Soc Lond B Biol Sci* 2015;**370**, DOI: 10.1098/rstb.2013.0559.
467. Aenishaenslin C, Page D, Gagnier M *et al.* Prioritization of areas for early detection of southward movement of arctic fox rabies based on historical surveillance data in Quebec, Canada. *Epidemiology and Infection* 2020;**149**:e20.
468. Hayes MA, Piaggio AJ. Assessing the potential impacts of a changing climate on the distribution of a rabies virus vector. *Plos One* 2018;**13**:e0192887.
469. Huettmann F, Magnuson EE, Hueffer K. Ecological niche modeling of rabies in the changing Arctic of Alaska. *Acta Vet Scand* 2017;**59**:18.
470. Lachica ZPT, Peralta JM, Diamante EO *et al.* A cointegration analysis of rabies cases and weather components in Davao City, Philippines from 2006 to 2017. *Plos One* 2020;**15**:e0236278.
471. Lee DN, Papeş M, Van den Bussche RA. Present and potential future distribution of common vampire bats in the Americas and the associated risk to cattle. *Plos One* 2012;**7**:e42466.
472. Kim BI, Blanton JD, Gilbert A *et al.* A Conceptual Model for the Impact of Climate Change on Fox

Rabies in Alaska, 1980-2010. *Zoonoses and Public Health* 2013;**61**:72–80.
473. Zarza H, Martinez-Meyer E, Suzan G *et al.* Geographic distribution of desmodus rotundus in Mexico under current and future climate change scenarios: Implications for bovine paralytic rabies infection. *Veterinaria México OA* 2017;**4**, DOI: https://dx.doi.org/10.21753/vmoa.4.3.390.
474. Thapa S, Baral S, Hu Y *et al.* Will climate change impact distribution of bats in Nepal Himalayas? A case study of five species. *Global Ecology and Conservation* 2021;**26**:e01483–NA.
475. Palasio RGS, Casotti MO, Rodrigues TC *et al.* The current distribution pattern of Biomphalaria tenagophila and Biomphalaria straminea in the northern and southern regions of the coastal fluvial plain in the state of São Paulo. *Biota Neotrop (Online, Ed ingl)* 2015;**15**:1–6.
476. Gong Y, Tong Y, Jiang H *et al.* Three Gorges Dam: Potential differential drivers and trend in the spatio-temporal evolution of the change in snail density based on a Bayesian spatial-temporal model and 5-year longitudinal study. *Parasit Vectors* 2023;**16**:232.
477. Pedersen UB, Karagiannis-Voules DA, Midzi N *et al.* Comparison of the spatial patterns of schistosomiasis in Zimbabwe at two points in time, spaced twenty-nine years apart: Is climate variability of importance? *Geospat Health* 2017;**12**:505.
478. Yang Y, Huang SY, Pei FQ *et al.* Spatial distribution and habitat suitability of Biomphalaria straminea, intermediate host of Schistosoma mansoni, in Guangdong, China. *Infect Dis Poverty* 2018;**7**:109.
479. Gong Y-F, Hu X-K, Hao Y-W *et al.* Projecting the proliferation risk of Oncomelania hupensis in China driven by SSPs: A multi-scenario comparison and integrated modeling study. *Advances in Climate Change Research* 2022;**13**:258–65.
480. Kalinda C, Chimbari MJ, Mukaratirwa S. Effect of temperature on the Bulinus globosus - Schistosoma haematobium system. *Infect Dis Poverty* 2017;**6**:57.
481. Camargo EAF, Camargo JTF, Neves MF *et al.* Assessment of the impact of changes in temperature in Biomphalaria glabrata (Say, 1818) melanic and albino variants infected with Schistosoma mansoni (Sambon, 1907). *Braz J Biol* 2017;**77**:490–4.
482. Knight M, Elhelu O, Smith M *et al.* Susceptibility of Snails to Infection with Schistosomes is influenced by Temperature and Expression of Heat Shock Proteins. *Epidemiology (Sunnyvale)* 2015;**5**, DOI: 10.4172/2161-1165.1000189.
483. Nguyen KH, Boersch-Supan PH, Hartman RB *et al.* Interventions can shift the thermal optimum for parasitic disease transmission. *Proc Natl Acad Sci U S A* 2021;**118**, DOI: 10.1073/pnas.2017537118.
484. Nguyen KH, Gemmell BJ, Rohr JR. Effects of temperature and viscosity on miracidial and cercarial movement of Schistosoma mansoni: Ramifications for disease transmission. *Int J Parasitol* 2020;**50**:153–9.
485. Wang X, Juma S, Li W *et al.* Potential risk of colonization of Bulinus globosus in the mainland of China under climate change. *Infect Dis Poverty* 2022;**11**:52.
486. McCreesh N, Nikulin G, Booth M. Predicting the effects of climate change on Schistosoma mansoni transmission in eastern Africa. *Parasit Vectors* 2015;**8**:4.
487. Stensgaard AS, Utzinger J, Vounatsou P *et al.* Large-scale determinants of intestinal schistosomiasis and intermediate host snail distribution across Africa: Does climate matter? *Acta Trop* 2013;**128**:378–90.
488. Yang GJ, Bergquist R. Potential impact of climate change on schistosomiasis: A global assessment attempt. *Trop Med Infect Dis* 2018;**3**, DOI: 10.3390/tropicalmed3040117.
489. Gao F, Ward MP, Wang Y *et al.* Implications from assessing environmental effects on spatio-temporal pattern of schistosomiasis in the Yangtze Basin, China. *Geospat Health* 2018;**13**, DOI: 10.4081/gh.2018.730.
490. Kalinda C, Chimbari MJ, Grant WE *et al.* Simulation of population dynamics of Bulinus globosus: Effects of environmental temperature on production of Schistosoma haematobium cercariae. *PLoS Negl Trop Dis* 2018;**12**:e0006651.
491. McCreesh N, Booth M. The effect of simulating different intermediate host snail species on the link between water temperature and schistosomiasis risk. *Plos One* 2014;**9**:e87892.
492. Palasio RGS, Azevedo TS de, Tuan R *et al.* Modelling the present and future distribution of Biomphalaria species along the watershed of the Middle Paranapanema region, São Paulo, Brazil. *Acta Trop* 2021;**214**:105764.
493. Ponpetch K, Erko B, Bekana T *et al.* Environmental Drivers and Potential Distribution of Schistosoma mansoni Endemic Areas in Ethiopia. *Microorganisms* 2021;**9**, DOI: 10.3390/microorganisms9102144.
494. Xue Z, Gebremichael M, Ahmad R *et al.* Impact of temperature and precipitation on propagation of intestinal schistosomiasis in an irrigated region in Ethiopia: Suitability of satellite datasets. *Trop Med Int*

Health 2011;**16**:1104–11.
495. Yang Y, Cheng W, Wu X *et al.* Prediction of the potential global distribution for Biomphalaria straminea, an intermediate host for Schistosoma mansoni. *PLoS Negl Trop Dis* 2018;**12**:e0006548.
496. Zhu G, Fan J, Peterson AT. Schistosoma japonicum transmission risk maps at present and under climate change in mainland China. *PLoS Negl Trop Dis* 2017;**11**:e0006021.
497. Tabo Z, Kalinda C, Breuer L *et al.* Adapting Strategies for Effective Schistosomiasis Prevention: A Mathematical Modeling Approach. *Mathematics* 2023;**11**, DOI: 10.3390/math11122609.
498. Kequn LIU, Xingjian XU, Yuxia C *et al.* Analysis on influence of meteorological factors on Oncomelania density. *Chinese Journal of Epidemiology* 2015;**12**:1274–8.
499. Nwoko OE, Manyangadze T, Chimbari MJ. Predicted changes in habitat suitability for human schistosomiasis intermediate host snails for modelled future climatic conditions in KwaZulu-Natal, South Africa. *Frontiers in Environmental Science* 2023;**11**:NA–.
500. Phillips C, Lipman GS, Gugelmann H *et al.* Snakebites and climate change in California, 1997-2017. *Clin Toxicol (Phila)* 2019;**57**:168–74.
501. Chaves LF, Chuang TW, Sasa M *et al.* Snakebites are associated with poverty, weather fluctuations, and El Niño. *Sci Adv* 2015;**1**:e1500249.
502. Ediriweera DS, Diggle PJ, Kasturiratne A *et al.* Evaluating temporal patterns of snakebite in Sri Lanka: The potential for higher snakebite burdens with climate change. *Int J Epidemiol* 2018;**47**:2049–58.
503. Lawing AM, Polly PD. Pleistocene climate, phylogeny, and climate envelope models: An integrative approach to better understand species' response to climate change. *Plos One* 2011;**6**:e28554.
504. Yañez-Arenas C, Townsend Peterson A, Rodríguez-Medina K *et al.* Mapping current and future potential snakebite risk in the new world. *Climatic Change* 2016;**134**:697–711.
505. Zacarias D, Loyola R. Climate change impacts on the distribution of venomous snakes and snakebite risk in Mozambique. *Climatic Change* 2019:195–207.
506. Chowdhury MAW, Müller J, Varela S. Climate change and the increase of human population will threaten conservation of Asian cobras. *Sci Rep* 2021;**11**:18113.
507. Costa M, Fonseca CSD, Navoni JA *et al.* Snakebite accidents in Rio Grande do Norte state, Brazil: Epidemiology, health management and influence of the environmental scenario. *Trop Med Int Health* 2019;**24**:432–41.
508. Goldstein E, Erinjery JJ, Martin G *et al.* Climate change maladaptation for health: Agricultural practice against shifting seasonal rainfall affects snakebite risk for farmers in the tropics. *iScience* 2023;**26**:105946.
509. Yousefi M, Yousefkhani SH, Grünig M *et al.* Identifying high snakebite risk area under climate change for community education and antivenom distribution. *Sci Rep* 2023;**13**:8191.
510. Nori J, Carrasco PA, Leynaud GC. Venomous snakes and climate change: Ophidism as a dynamic problem. *Climatic Change* 2013;**122**:67–80.
511. Rocha S, Pinto RM, Floriano AP *et al.* Environmental analyses of the parasitic profile found in the sandy soil from the Santos municipality beaches, SP, Brazil. *Rev Inst Med Trop Sao Paulo* 2011;**53**:277–81.
